# Shining Fresh Light on Complex Photoredox Mechanisms through Isolation of Intermediate Radical Anions

**DOI:** 10.1021/acscatal.3c02515

**Published:** 2023-06-30

**Authors:** Samuel J. Horsewill, Gabriele Hierlmeier, Zahra Farasat, Joshua P. Barham, Daniel J. Scott

**Affiliations:** † Department of Chemistry, University of Bath, Claverton Down, Bath BA2 7AY, UK; ‡ Department of Chemistry, Princeton University, Princeton, New Jersey 08544, United States; § Professor Rashidi Laboratory of Organometallic Chemistry, Department of Chemistry, College of Sciences, Shiraz University, Shiraz, Fars 71467-13565, Iran; ∥ Institute of Organic Chemistry, University of Regensburg, Universitätsstr. 31, Regensburg, Bayern 93053, Germany

**Keywords:** conPET, e-PRC, photocatalysis, photochemistry, photoredox, radical ions

## Abstract

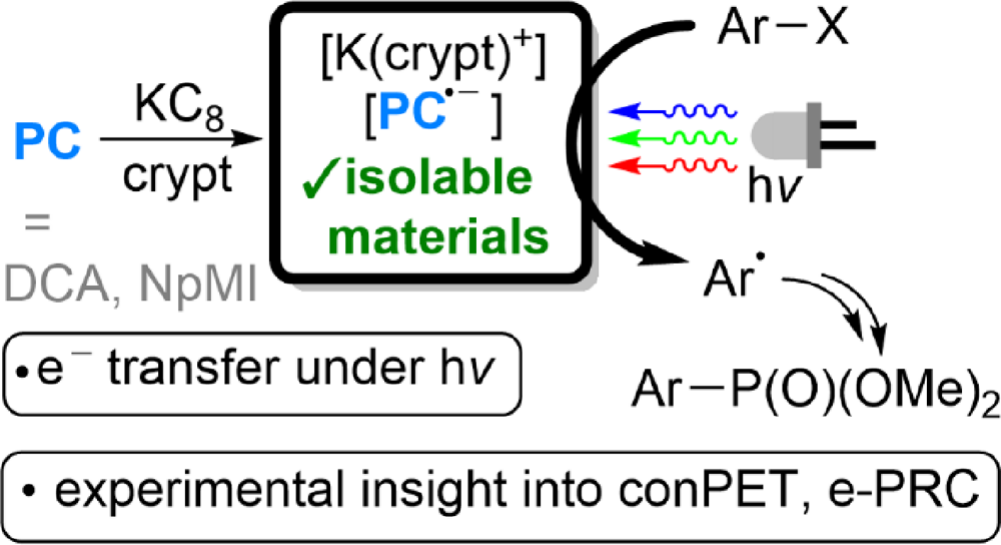

Photoredox catalysis (PRC) has gained enormous and wide-ranging interest in recent years but has also been subject to significant mechanistic uncertainty, even controversy. To provide a method by which the missing understanding can begin to be filled in, we demonstrate herein that it is possible to isolate as authentic materials the one-electron reduction products of representative PRC catalysts (PCs). Specifically, KC_8_ reduction of both 9,10-dicyanoanthracene and a naphthalene monoamide derivative in the presence of a cryptand provides convenient access to the corresponding [K(crypt)^+^][PC^·–^] salts as clean materials that can be fully characterized by techniques including EPR and XRD. Because PC^·–^ states are key intermediates in PRC reactions, such isolation allows for highly controlled study of these anions’ specific reactivity and hence their mechanistic roles. As a demonstration of this principle, we show that these salts can be used to conveniently interrogate the mechanisms of recent, high-profile “conPET” and “e-PRC” reactions, which are currently the subject of both significant interest and acute controversy. Using very simple experiments, we are able to provide striking insights into these reactions’ underlying mechanisms and to observe surprising levels of hidden complexity that would otherwise have been very challenging to identify and that emphasize the care and control that are needed when interrogating and interpreting PRC mechanisms. These studies provide a foundation for the study of a far broader range of questions around conPET, e-PRC, and other PRC reaction mechanisms in the future, using the same strategy of PC^·–^ isolation.

## Introduction

The rapid emergence, adoption, and expansion of photoredox catalysis (PRC) has been one of the more dramatic and significant developments in synthetic chemistry of the past decade.^1,2^ By using light (typically visible light) to selectively photoexcite a redox-active dye, PRC allows synthetic chemists to access versatile and reactive radical intermediates in a remarkably controllable manner. This has allowed practitioners to develop a myriad of new reactions, often involving the transformation of relatively ‘unactivated’ substrates, and PRC methods have rapidly become a mainstay of modern chemistry research.^3,4^

**Scheme 1:**
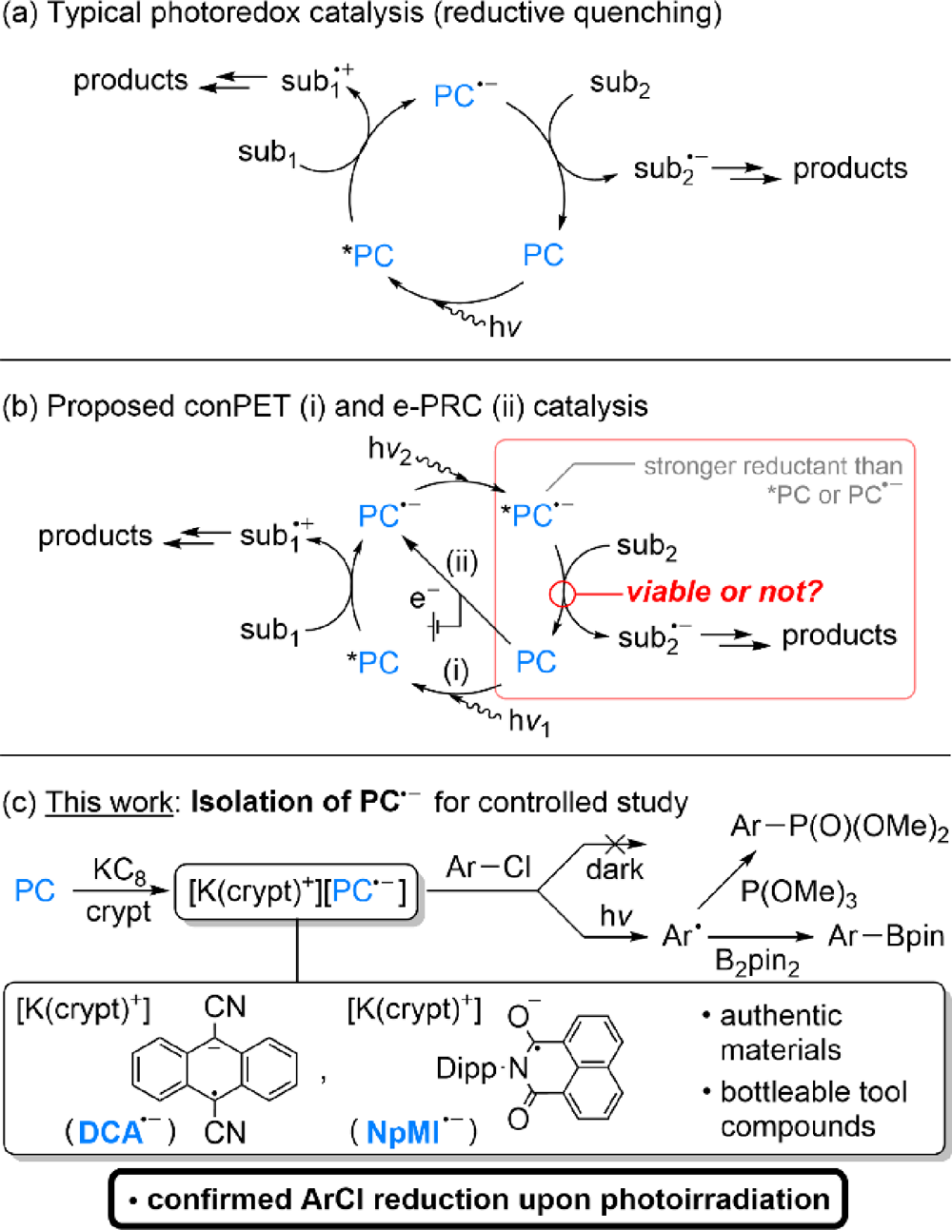
Generic Mechanisms for PRC Reactions (a) A generic mechanism for ‘standard’ PRC reactions proceeding *via* reductive quenching of photocatalyst excited state *PC (analogous oxidative quenching is also possible, forming PC^·+^ in place of PC^·–^); (b) generic mechanisms proposed for reductive conPET (i) and e-PRC (ii) reactions; and (c) isolation of key PC^·–^ intermediates allowing for direct experimental interrogation of these mechanisms, as described herein. In panels (a) and (b), sub_1_/sub_2_ may be separate reaction substrates or intermediates derived from one another.

At the heart of PRC lies a relatively simple mechanistic rationale, in which photoexcitation of a suitable photocatalyst dye (PC) leads to a markedly more oxidizing (or reducing) excited state (*PC), which can engage in single-electron transfer (SET) with a target substrate (Scheme 1a). The resulting radical PC^·–^ (or PC^·+^) can then engage in a second SET step to close the catalytic cycle, having in the process generated one or more reactive substrate-derived radicals, sub^·–^/sub^·+^, which can then engage in subsequent, reaction-specific elementary steps to afford the final reaction products.

Unfortunately, the “deceptive simplicity”^5^ of this mechanistic picture obscures the fact that in practice, PRC mechanisms are often highly complex and challenging to study, especially for steps that occur ‘downstream’ of initial *PC quenching or involve short (or ultrashort) excited state lifetimes. As a result, they are often poorly understood, and inspection of the recent literature reveals common, explicit concerns among practitioners about the negative impact of this limitation on current PRC research.^6^ On those occasions where specific PRC mechanisms have been studied in detail, the results have typically served to highlight unexpected levels of complexity in the reaction mechanisms in question.^5−7^

There is thus a clear need for new methods by which the mechanisms of PRC catalysis might conveniently be interrogated. One such solution would be the careful isolation of the key intermediates involved in PRC reactivity, to allow for study of their elementary reactivity under fully controlled conditions (a strategy that has been foundational to the understanding of many other homogeneous catalytic reactions). Notably, their catalytic versatility and outer-sphere reactivity mean that for any single PC, there typically exist many different reactions, all believed to proceed *via* exactly the same PC^·–^ (or PC^·+^) state. Isolation of just one such PC^·–^ would thus provide a tool compound to study many distinct PRC transformations. However, such a strategy has seldom been pursued until now, and its feasibility has thus remained unproven and uncertain.^4d,8^

An excellent example of the confusion that can surround PRC mechanisms is found in the related areas of “conPET” (consecutive photoinduced electron transfer) and “electrochemically-mediated PRC” (e-PRC).^9,10^ These are adaptations of the ‘standard’ PRC mechanism that have both attracted particularly intense interest in recent years as they allow the catalysts employed to generate extremely reducing (or oxidizing)^11^ potentials that exceed those normally accessible in visible light PRC (though there is no strict limit, potentials beyond *ca.* ±2 V *vs* SCE are commonly considered difficult to achieve for standard PRC).^12^ This allows for the facile activation of challenging, inert substrates, such as reduction of electron-neutral (or even electron-rich) aryl bromides and chlorides.^13^ To achieve this, reductive conPET mechanisms are proposed to proceed *via* initial formation of PC^·–^ from PC in the same manner as for ‘standard’ PRC (Scheme 1b, i). However, once formed, this reductant is then suggested to undergo a second photoexcitation, thus increasing its reducing power considerably. Substrate reduction then occurs *via* SET from this excited state, *PC^·–^. A very similar rationale is proposed in e-PRC reactions, although in this case the ground-state PC^·–^ is formed through direct, electrochemical reduction of ground-state PC (Scheme 1b, ii). Thus, while conPET and e-PRC differ in the number of photons required in their catalytic cycles, and often in their downstream redox chemistry,^9d^ the same excited state (*PC^·–^) is invoked as a crucial catalytic intermediate in both cases. However, the viability of the key electron transfer step from these intermediates has attracted significant controversy. A more detailed discussion of this point can be found below; however, in essence, it has been argued that the doublet excited states *PC^·–^ are simply too short-lived to engage in the proposed, presumably diffusion-limited, intermolecular SET.

Unfortunately, direct experimental interrogation of this step and of the photochemistry of relevant PC^·–^ in general has typically proven extremely challenging and relied on advanced and specialized spectroscopic techniques that are not easily accessible for most practitioners in the field (*cf.* bench-stable, closed-shell PCs, which can typically be studied by accessible, established techniques such as fluorescence quenching). These studies have in turn been reliant on photo- or electrochemical methods to generate the air-sensitive PC^·–^ intermediates *in situ*.^14^ These methods increase practical complexity, while also significantly complicating subsequent analysis and interpretation due to the inevitable presence of species other than PC^·–^ in the analyte mixture (e.g., electrolytes, stoichiometric reductant-derived byproducts, *etc*.), whose impact is often uncertain and must be rigorously accounted for. Formation of side products is also possible and can easily be overlooked, leading to erroneous conclusions (*vide infra*).

ConPET and e-PRC thus provide excellent illustrative examples of where the study of isolated PC^·–^ would be highly valuable. With this in mind, we report herein that relevant PC^·–^ salts containing non-interacting counter-cations can be isolated as authentic, ‘bottleable’ materials (Scheme 1c).^15^ This allows for direct study of their chemical and photochemical behavior, providing insights of clear relevance to their conPET and e-PRC reactivity. The results highlight significant complexities that lie hidden in these reactions and challenge arguments that have previously been made about their mechanisms.

## Results and Discussion

### Synthesis and Characterization of [K(crypt)^+^][DCA^·–^]

Although many different PC are available, for this initial study, it was decided to target reduction of the relatively simple organic photocatalysts **DCA** (9,10-dicyanoanthracene) and **NpMI** (a naphthalene monoimide derivative), which were chosen based on the prominent roles they have played in the development of conPET and e-PRC as well as the controversy and confusion that continue to surround it (*vide infra*).^16^ The reduction potentials for both should also be chemically accessible (*ca.* −1.0 V, −1.3 V *vs* SCE for **DCA**, **NpMI**, respectively).^9d,16b^

**Scheme 2:**
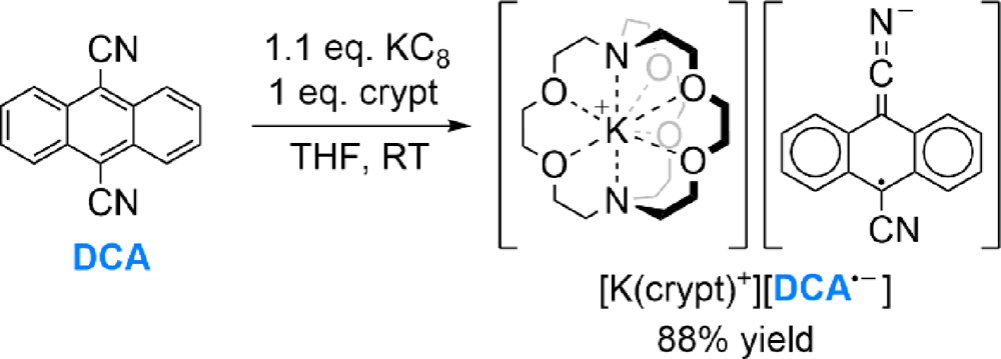
Synthesis of [K(crypt)^+^][DCA^·–^] Only one of several possible resonance forms of the **DCA**^·–^ anion is shown.

Thus, a suspension of **DCA** in THF was treated under an inert atmosphere with an equimolar quantity of 2,2,2-cryptand (crypt) and 1.1. equiv of KC_8_, which led to immediate formation of a deep purple solution. Filtration to remove excess KC_8_ and the graphite byproduct and removal of solvent *in vacuo* followed by washing with PhMe and hexane yielded a dark solid, which could be identified as the target product [K(crypt)^+^][**DCA^·–^**], in very good yield (88%; Scheme 2).^17^

**Figure 1:**
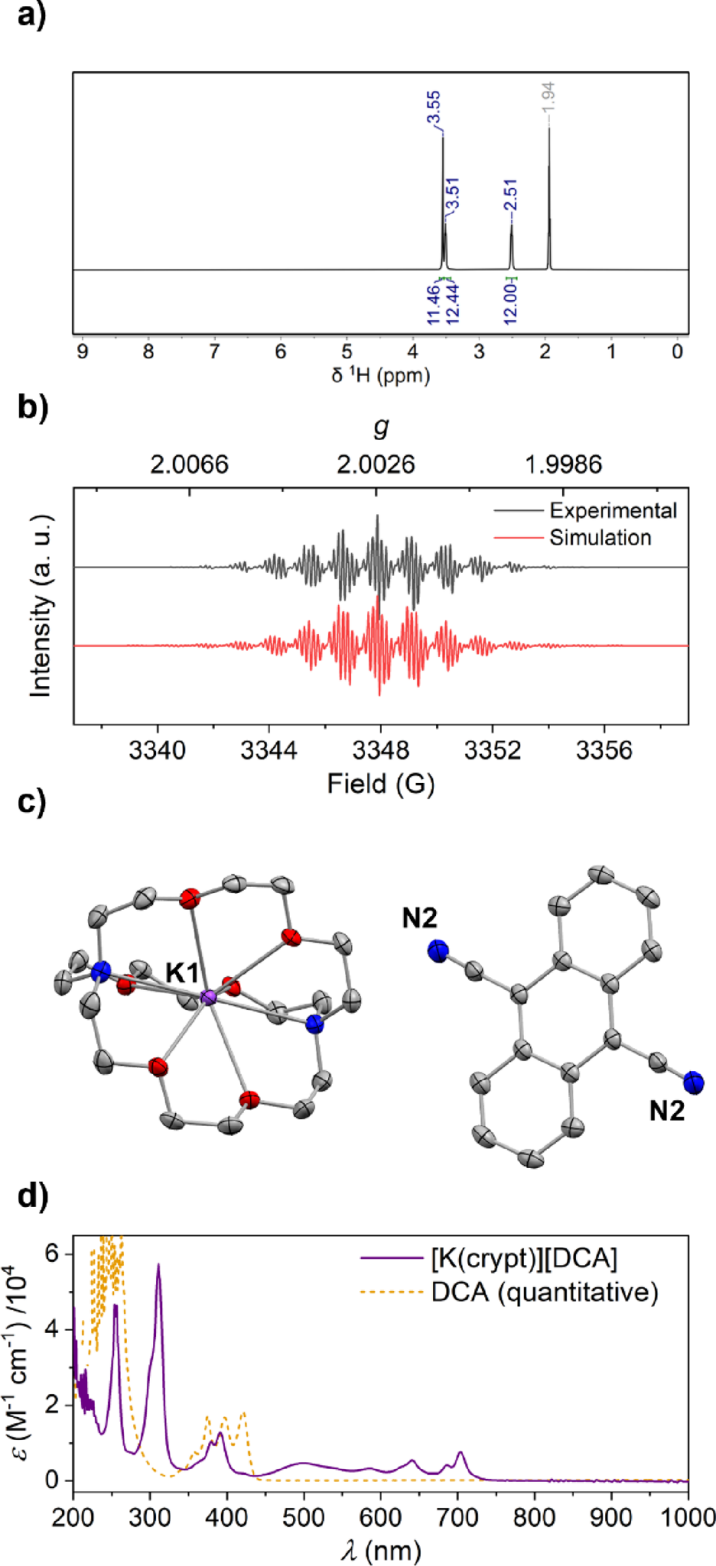
Spectroscopic and crystallographic characterization of [K(crypt)^+^][**DCA^·–^**] (additional details in the SI): (a) ^1^H NMR spectrum (CD_3_CN (δ *=* 1.94 ppm), RT); (b) X-band EPR spectrum (4:1 PhMe/THF, 109 μM, RT; simulated as *g* = 2.00256, 4 × *A*(^1^H) = 3.904 MHz, 4 × *A*(^1^H) = 2.969 MHz, 2 × *A*(^14^N) = 0.4307 MHz, 15% *A*(^13^C) = 20.631 MHz); (c) single-crystal XRD structure; thermal ellipsoids shown at 50%; H atoms omitted for clarity and only one of two inequivalent **DCA** moieties shown (asymmetric unit contains 1 × [K(crypt)^+^] and 2 × 0.5[**DCA**^·–^]); C atoms in gray, N in blue, O in red, K in purple; (d) UV–vis spectrum (MeCN, 544 μM, 1 mm path, RT).

The identity of the product was confirmed by a combination of spectroscopic and crystallographic analysis (*vide infra*) and, crucially, its compositional purity could be confirmed by elemental analysis. As expected, room-temperature NMR analysis of the product showed only the ^1^H and ^13^C resonances expected for the cryptand moiety, with no signals detected for the paramagnetic **DCA^·–^** anion in the ±500 ppm range (Figure 1a). The corresponding X-band EPR spectrum showed a clear signal at the frequency expected for an organic radical (*g* = 2.00256) and clear hyperfine coupling to two equivalent N atoms and two sets of four equivalent H atoms, fully in line with the expected delocalization of spin density across the entire **DCA** moiety (Figure 1b).

Further conclusive proof of the product structure was provided by XRD analysis of single crystals grown by slow diffusion of Et_2_O into a THF solution. The structure is depicted in Figure 1c and reveals two crystallographically independent half **DCA^·–^** moieties per asymmetric unit, whose charges are balanced by a single K(crypt)^+^ site (for full details, see Section 5.1 of the SI). Both **DCA^·–^** moieties are fully planar and show no close interactions with the K^+^ cation, which as expected is fully sequestered by the cryptand. Comparison of the **DCA^·–^** bond lengths with those reported for neutral **DCA** reveal a pattern of bond contractions and elongations consistent with the nodal structure of the SOMO,^18^ in line with previous predictions (Figure S60).^19^ The UV–vis spectrum of [K(crypt)][**DCA^·–^**] is shown in Figure 1d and is consistent with several previous *in situ* spectra assigned to **DCA^·–^**.^20^

### Synthesis and Characterization of [K(crypt)^+^][NpMI^·–^]

**Scheme 3:**
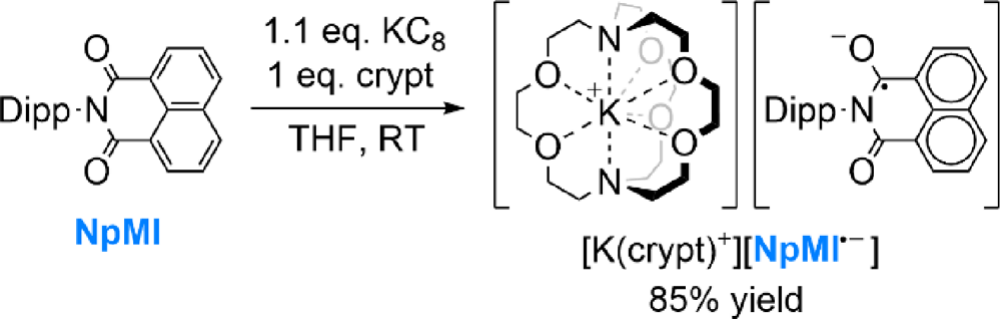
Synthesis of [K(crypt)^+^][NpMI^·–^] Only one of several possible resonance forms of the **NpMI**^·–^ anion is shown. Dipp = 2,6-di*iso*propylphenyl.

Replacing **DCA** with **NpMI** in the previous synthesis led to an essentially equivalent outcome. In this case, a deep green solid was isolated, again in excellent yield, which could be identified as the desired salt [K(crypt)^+^][**NpMI^·–^**] through a similar combination of spectroscopic, crystallographic, and elemental analysis (Scheme 3).

**Figure 2:**
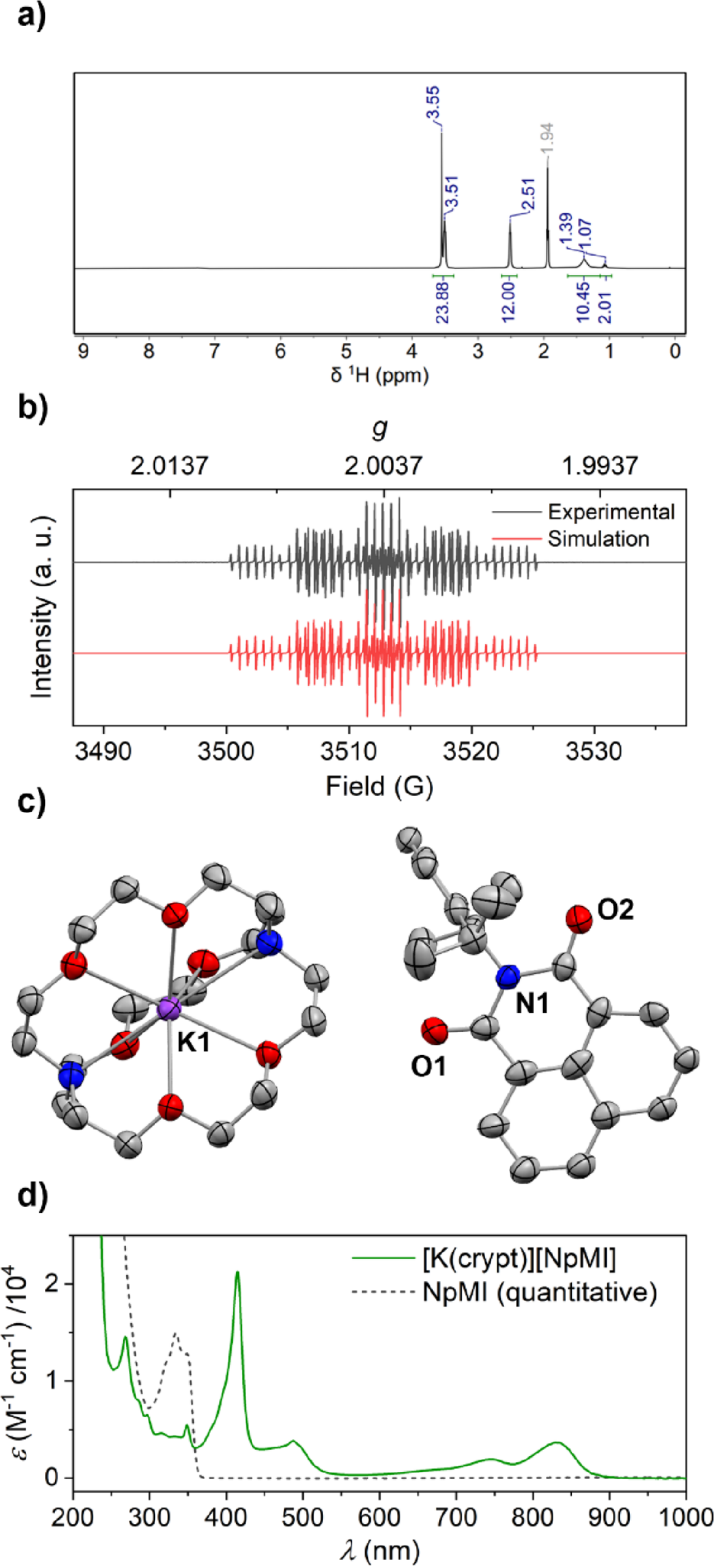
Spectroscopic and crystallographic characterization of [K(crypt)^+^][**NpMI^·–^**] (additional details in the SI): (a) ^1^H NMR spectrum (CD_3_CN (δ *=* 1.94 ppm), RT); (b) X-band EPR spectrum (THF, 199 μM, RT; simulated as *g* = 2.00371, 2 × *A*(^1^H) = 15.833 MHz, 2 × *A*(^1^H) = 13.357 MHz, 2 × *A*(^1^H) = 1.833 MHz, *A*(^14^N) = 3.760 MHz); (c) single-crystal XRD structure of [K(crypt)^+^][**NpMI^·–^**]·(THF); thermal ellipsoids shown at 50%; H atoms, THF molecule and disorder in **NpMI** moiety omitted for clarity; C atoms in gray, N in blue, O in red, K in purple; (d) UV–vis spectrum (MeCN, 504 μM, 1 mm path, RT).

As for [K(crypt)^+^][**DCA^·–^**], ^1^H and ^13^C NMR analysis of [K(crypt)^+^][**NpMI^·–^**] showed the expected resonances for the cryptand ligand (at 3.55, 3.51, 2.51 ppm). However, in this case, a pair of broad ^1^H features were also observable at 1.39 and 1.07 ppm, attributed to the *i*Pr moieties of the **NpMI^·–^** Dipp group (Figure 2a; Dipp = 2,6-di*iso*propylphenyl). This assignment is supported by their relative integral intensities and suggests localization of radical character on the electron-deficient naphthalene monoimide fragment rather than on the Dipp group, as expected. This interpretation is supported by the corresponding X-band EPR spectrum, which shows hyperfine coupling to one N atom and three sets of two equivalent H atoms (Figure 2b). This is consistent with previous EPR investigations of *in situ* electrogenerated **NpMI^·–^**, although these provided much less well-defined, pseudo-quintet signals.^16a^

The crystal structure of [K(crypt)^+^][**NpMI^·–^**] reveals a single K(crypt)^+^ cation and a single **NpMI^·–^** environment (Figure 2c). The naphthalene fragment of the latter is disordered, and so detailed bond lengths will not be discussed. However, the naphthalene monoimide moiety is clearly planar, with no evidence of significant, residual, out-of-plane electron density that might indicate C(sp^3^) sites (*cf.* [**NpMI**·H^–^]; ref (21)). The Dipp moiety is twisted almost perpendicular to this plane, in line with the lack of spin delocalization into this part of the anion.

The UV–vis spectrum of [K(crypt)^+^][**NpMI^·–^**] is shown in Figure 2d and confirms the spectroscopic signature of the radical anion, being consistent with previous *in situ* observations.^16a,21,22^

### Prior State of the Art Regarding conPET and e-PRC Mechanisms

For context, it should be noted that the mechanistic controversies surrounding synthetic applications of conPET trace back to the origins of the field. In 2014, König *et al*. reported reduction of aryl halides using a perylene diimide derivative as the proposed conPET catalyst,^23^ but the mechanism was challenged in 2017 by Cozzi *et al.* based on lifetime arguments (*vide supra*).^24^ In contrast, the mechanism has been supported by a more recent report from Zhang *et al.*, although the authors emphasized the need for very high substrate concentrations.^25^

Similarly, the initial independent reports of synthetic e-PRC in 2020 by Wickens *et al.* (using **NpMI**)^26^ and Lambert *et al.* (using **DCA**)^19^ have also attracted considerable comment (note that **DCA** has been used as a photocatalyst for several proposed conPET reactions, as well as e-PRC).^27^ In their original report, Lambert *et al.* pointed to a published excited state lifetime for *DCA^·–^ of 13.5 ns, which was expected to be long enough to allow for diffusive encounters with substrate molecules and subsequent SET (though highly concentration-dependent, it has been suggested as a rule of thumb that this usually requires lifetimes >1 ns).^28^ Unfortunately, reinspection of the prior literature revealed that this value, which was based on fluorescence quenching of *in situ* electrochemically generated solutions of **DCA^·–^**, had subsequently been found to be erroneous.^29^ In fact, **DCA^·–^** had been reported in a later study to be non-fluorescent, with the previously observed emission being attributed to minor formation of another species due to the presence of trace O_2_ during *in situ* electrolysis. The authors of the latter report even went so far as to state that “because the excited-state lifetimes of typical radical ions are short, they are poor candidates for use as photosensitizers”.^30^ This confusion neatly highlights the dangers of relying on *in situ* methods for PC^·–^ generation.

**Scheme 4:**
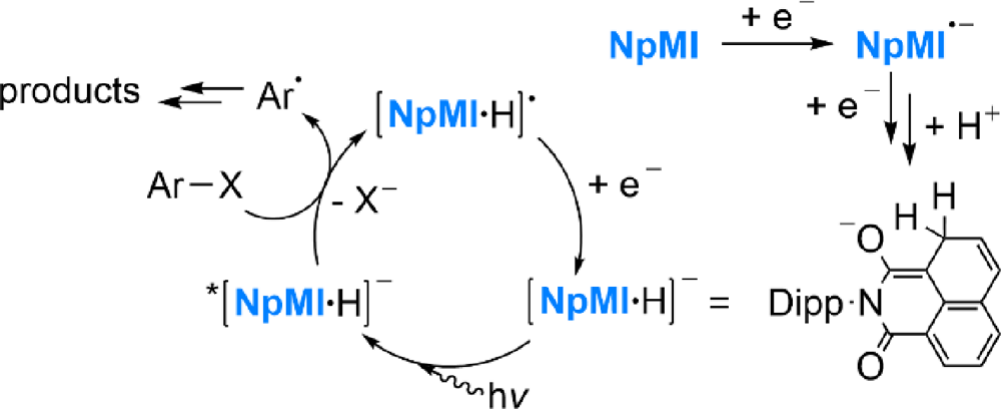
Alternative Mechanism for Photoreduction of Challenging Substrates Mediated by NpMI as (Pre-)catalyst Proposed by Nocera *et al*(21) Dipp = 2,6-di*iso*propylphenyl.

Subsequent attempts in the 1990s to clarify the lifetime of *DCA^·–^ also relied on *in situ* methods and gave diverging estimates.^31^ However, a very recent study by Vauthey *et al.* has indicated a value as low as a few picoseconds.^32^ Such short lifetimes are commonly observed for simple organic doublet anions,^33,34^ and with this in mind, Nocera *et al.* have recently argued that **NpMI**-catalyzed reactions proposed to proceed *via* e-PRC may instead be the result of *in situ* transformation of **NpMI**/**NpMI^·–^** into the Meisenheimer-type structure [**NpMI**·H^–^] (Scheme 4), which as a closed-shell species has a much longer excited state lifetime (20 ns) and is therefore suggested to be the true active catalyst.^21^ The authors imply that similar processes may account for other observations previously attributed to conPET/e-PRC. Others, however, have argued that intermolecular SET from *PC^·–^ could be feasible despite their short lifetimes under certain circumstances (for example due to static and non-stationary quenching regimes that are not diffusion-limited)^8,13g,32^ and such steps continue to be invoked in reports of new PRC reactions, albeit often accompanied by an acknowledgement of some mechanistic uncertainty.^35^

Clearly, the question of *[PC^·–^] involvement in conPET and e-PRC mechanisms is both highly complex and rather fraught, and a comprehensive resolution of the controversy is beyond the scope of a single manuscript (*vide infra*). Nevertheless, in order to highlight and emphasize the experimental opportunities made available by isolation of authentic [K(crypt)^+^][PC^·–^], we present below some initial experiments toward this goal that provide important new insights while also highlighting new directions for future work.

### Photoreactivity of Isolated [K(crypt)^+^][DCA^·–^]

To begin, solutions of [K(crypt)^+^][**DCA**^·–^] in MeCN were simply irradiated under various LED wavelengths in the absence of any substrate. After 16 h, no appreciable change was observed either by UV–vis or NMR spectroscopy at any of the wavelengths studied, suggesting that any subsequent photoreactivity cannot easily be attributed to photodecomposition products (see SI, Section 3.1.1).

**Figure 3:**
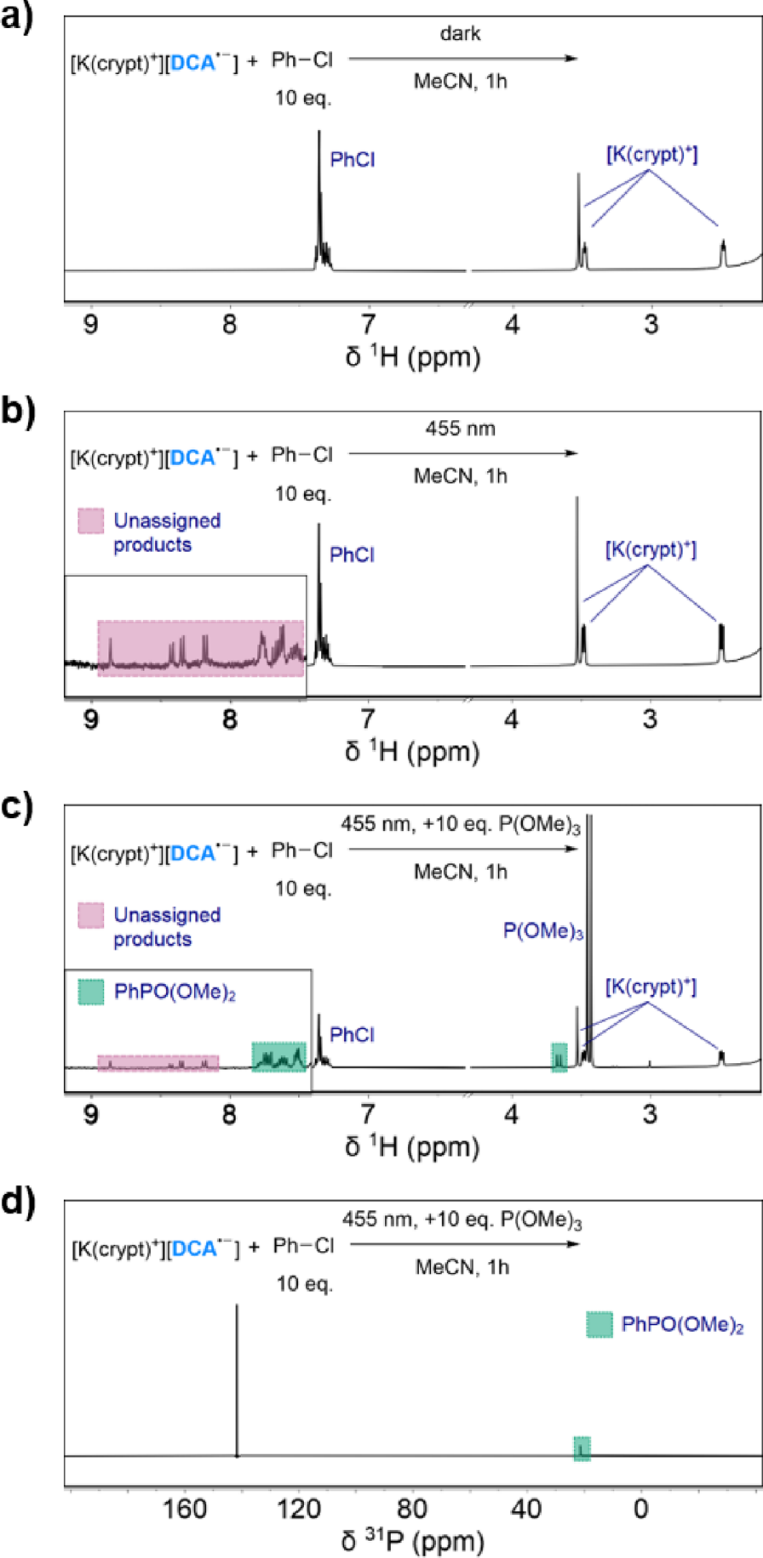
^1^H NMR spectra for the reaction of [K(crypt)^+^][**DCA**^·–^] with PhCl (10 equiv) in MeCN for 1 h (a) in the dark or (b) under 455 nm LED irradiation; and (c) ^1^H and (d) ^31^P{^1^H} NMR spectra for the latter reaction performed in the presence of P(OMe)_3_ (10 equiv). Certain ranges are inset and magnified for clarity. Additional spectra can be found in Section 3 of the SI.

Following this confirmation of its photostability, [K(crypt)^+^][**DCA**^·–^] was then combined with an excess of a simple yet challenging model substrate, PhCl, at concentrations representative of those that have been used in previous catalytic reactions (5 mM in [K(crypt)^+^][**DCA^·–^**], 50 mM in PhCl).^36^ As expected, no reaction was observed in the dark (Figure 3a), either by eye or by ^1^H NMR spectroscopy.

The reaction was then repeated under blue (455 nm) LED irradiation. Failure to observe reactivity in this experiment would unambiguously preclude the conPET and e-PRC mechanisms previously proposed for **DCA** at this wavelength. However, upon irradiation, clear reactivity was observed, with a loss of the characteristic purple color of **DCA^·–^** and the formation of new signals in the ^1^H NMR spectrum (Figure 3b). Moreover, formation of PhH (and trace Ph_2_) as downstream Ph^·^-derived products was clearly confirmed by GC–MS analysis (see SI, Section 3.4.1), consistent with reduction of PhCl to Ph^·^. However, ^1^H NMR analysis of the reaction also revealed a large number of other new resonances in the aromatic region, which is presumably attributable to attack of initially formed Ph^·^ radicals on the remaining **DCA^·–^** anions (which would be expected to outcompete homocoupling due to low Ph^·^ concentrations). Such reactions are known for similar cyanoarene motifs and typically involve displacement of cyanide (for further discussion, see Section 3.5.1 of the SI).^37^

To provide a neater outcome, the reaction between [K(crypt)^+^][**DCA^·–^**] and PhCl was repeated in the presence of a radical trap, with P(OMe)_3_ being chosen to provide a convenient ^31^P NMR handle. Simple alkyl phosphites are known to function as effective traps for aryl radicals generated during PRC (including proposed conPET), generating phosphonate esters.^27b,38^ In this case, a much cleaner outcome was observed, with a single set of major new signals in the ^1^H spectrum and only one significant new peak in the ^31^P{^1^H} spectrum, at 21.2 ppm (Figure 3c,d). These signals correspond to PhP(O)(OMe)_2_ (confirmed by spiking the mixture with authentic material), which is the expected final product of Ph^·^ radical trapping by P(OMe)_3_. Control reactions showed no significant reaction between [K(crypt)^+^][**DCA^·–^**] and P(OMe)_3_ in the absence of PhCl (either under irradiation or in the dark). These observations are therefore again fully consistent with net reduction of PhCl by **DCA^·–^** to generate Ph^·^ (Table 1). Quantitative ^31^P{^1^H} integration relative to a subsequently added internal standard showed 66% conversion to PhP(O)(OMe)_2_ relative to the amount of [K(crypt)^+^][**DCA^·–^**] added, after 1 h (Table 1, entry 1).^39^

**Table 1:**
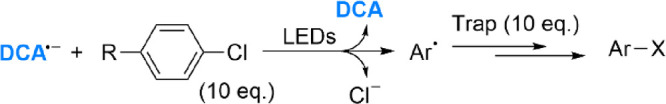
Conversion of ArCl to ArX *via* Reduction to Ar^·^ Using [K(crypt)^+^][DCA^·–^] and Subsequent Trapping^a^

entry	trap^b^	*t*/h	R	λ/nm	conv./%^c^
1	P(OMe)_3_	1	H	455	66
2	P(OMe)_3_	1	H	530	0
3	P(OMe)_3_	1	H	630	0
4	P(OMe)_3_	1	H	730	0
5	P(OMe)_3_	1	CO_2_Me	455	74
6	P(OMe)_3_	1	CO_2_Me	530	10
7	P(OMe)_3_	1	CO_2_Me	630	7
8	P(OMe)_3_	1	CO_2_Me	730	8
9	P(OMe)_3_	1	OMe	455	95^d^
10	P(OMe)_3_	1	OMe	530	0
11	P(OMe)_3_	1	OMe	630	0
12	P(OMe)_3_	1	OMe	730	0
13	P(OMe)_3_	16	H	455	174^e^
14	P(OMe)_3_	16	H	530	3
15	P(OMe)_3_	16	H	630	0
16	P(OMe)_3_	16	H	730	0
17	P(OMe)_3_	16	CO_2_Me	455	247^e^
18	P(OMe)_3_	16	CO_2_Me	530	82
19	P(OMe)_3_	16	CO_2_Me	630	77
20	P(OMe)_3_	16	CO_2_Me	730	37
21	P(OMe)_3_	16	OMe	455	141^d^^,^^e^
22	P(OMe)_3_	16	OMe	530	0
23	P(OMe)_3_	16	OMe	630	0
24	P(OMe)_3_	16	OMe	730	0
25	B_2_pin_2_	16	CO_2_Me	455	58
26	P(OEt)_3_	16	CO_2_Me	455	257

a Reaction between ArCl (10 equiv, 50 mM), trap (10 equiv, 50 mM), and [K(crypt)^+^][**DCA^·–^**] (1 equiv, 5 mM) in MeCN under LED irradiation (peak wavelength λ).

b For P(OMe)_3_, P(OEt)_3_, and B_2_pin_2_ as traps, X = P(O)(OMe)_2_, P(O)(OEt)_2_, and Bpin, respectively.

c Conversion to ArX measured by quantitative ^31^P{^1^H} (X = P(O)(OMe)_2_, P(O)(OEt)_2_), or ^1^H (X = Bpin) NMR spectroscopy relative to a subsequently added internal standard (Ph_3_PO or mesitylene), relative to the initial amount of [K(crypt)^+^][**DCA^·–^**] added.

d An additional, unidentified signal was also observed at δ(^31^P) = 17.3 ppm, integrating to 40% (entry 9), 51% (entry 21).

e Average of three experiments (see Section 3.4 of the SI).

The reaction between [K(crypt)^+^][**DCA^·–^**], PhCl, and P(OMe)_3_ was also investigated under irradiation at other wavelengths. While in their e-PRC report, Lambert *et al.* used blue LEDs,^19^ others have previously argued that green light rather than blue is essential for productive photoexcitation of **DCA^·–^**,^27a,27b^ while the Wenger group has recently used red light.^27c^ However, in our hands, neither green (530 nm) nor red (630 nm) LED irradiation yielded measurable product formation under otherwise equivalent conditions (Table 1, entries 2 and 3). Nevertheless, when PhCl was replaced with a more electron-deficient and hence easier to reduce substrate bearing a 4-CO_2_Me group, the reaction proceeded at both wavelengths (albeit considerably more slowly at both wavelengths than at 455 nm; Table 1, entries 5–7) and even at 730 nm, which is at the limit of the **DCA**^·–^ absorption window (Table 1, entry 8).

All three previously employed wavelengths are thus clearly competent for the aryl chloride reduction step, but with strong substrate dependence. Moreover, higher reactivity is consistently observed at 455 nm (Table 1, *cf.* entry 5 and entries 6–8), despite seemingly poor absorption by **DCA^·–^** around this wavelength (*cf.*Figure 1d, and *vide infra* for additional discussion). Indeed, under 455 nm LEDs, reduction could even be achieved for the more electron-rich, 4-OMe-substituted substrate 4-chloroanisole (Table 1, entries 9–12). No obvious correlation is apparent between the conversions observed at this wavelength and the electron richness/deficiency of these substrates (Table 1, *cf.* entries 1, 5, and 9).

As expected, increased reaction times allowed for significant improvements in substrate conversion (Table 1, entries 13–24). To our surprise, however, sufficiently extended irradiation times were consistently found to lead to conversions greater than 100% with respect to [K(crypt)^+^][**DCA^·–^**], particularly at 455 nm. This suggests that **DCA^·–^** is capable not only of mediating the reaction between ArCl and P(OMe)_3_ upon photoirradiation, but actually of catalyzing it, with modest turnover, even in the absence of an external reductant. Even higher turnovers could be observed by using lower [K(crypt)^+^][**DCA^·–^**] concentrations (for details, see Section 3.4.4 of the SI).

**Scheme 5:**
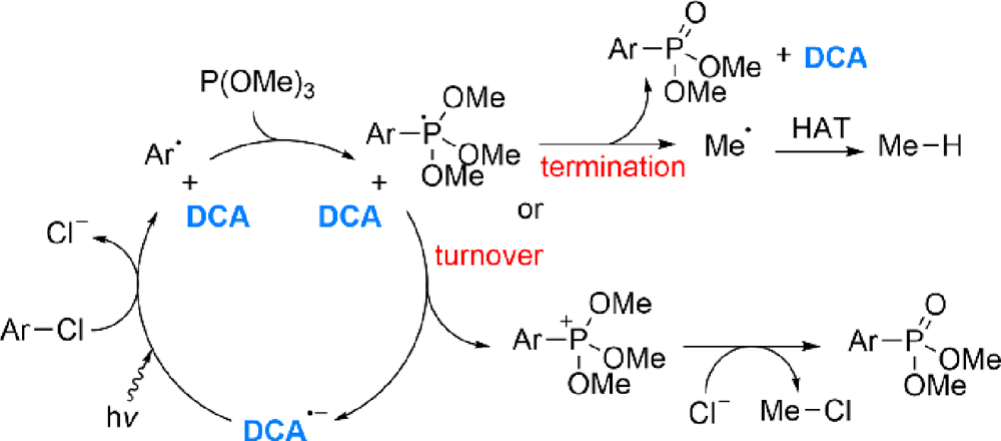
Proposed Catalytic Mechanism for the Reaction of ArCl and P(OMe)_3_, Mediated by [K(crypt)^+^][DCA^·–^]

These unexpected observations further emphasize the hidden complexity that can exist in even seemingly simple photoredox reactions and that would be difficult to identify in less controllable, *in situ* studies. They are tentatively attributed to the catalytic mechanism summarized in Scheme 5. Simple phosphites P(OR)_3_ are well known as effective traps for aryl radicals Ar^·^, which add to form P-centered phospharanyl radicals ArP(OR)_3_^·^. Transformation of these radicals into the final observed products ArP(O)(OR)_2_ requires formal loss of R^·^, and in previous PRC studies (including conPET), this has generally been proposed to occur directly *via* known β-fragmentation pathways, with the resulting R^·^ then abstracting a H atom from the reaction mixture to produce RH as the stoichiometric byproduct.^38,40^ However, it is known that the radical PhP(OMe)_3_^·^ is also a potent reductant and that intermolecular SET from PhP(OMe)_3_^·^ can be fast enough to compete with β-fragmentation.^41^ PhP(OMe)_3_^·^ is at least reducing enough to reduce the sulfonium cation TolSPh_2_^+^ (Tol = 4-tolyl) and, based on the potentials required to reduce similar sulfonium salts (e.g., *ca.* −1.5 V *vs* SCE for Ph–TT^+^; TT = thianthrene),^42^ should also be capable of reducing neutral **DCA** back to the radical anion **DCA^·–^** (*ca*. −1.0 V *vs* SCE),^16b^ hence providing turnover.^43^ Arbuzov-type loss of MeCl *via* attack of chloride on concomitantly-generated PhP(OMe)_3_^+^ would then furnish the phosphonate product.^44^

To provide qualitative support for this interpretation, the highest-turnover reaction (using MeO_2_CC_6_H_4_Cl and blue LEDs) was repeated using B_2_pin_2_ as an alternative radical trap instead of P(OMe)_3_. In this case, an analogous redox-neutral coupling pathway is not available and indeed only (sub)stoichiometric formation of the ArBpin product was observed, even with the extended reaction time (Table 1, entry 25). For thoroughness, an alternative, commonly used phosphite trap, P(OEt)_3_ was also tested under the same conditions and again led to greater than stoichiometric conversion (Table 1, entry 26).

### Photoreactivity of Isolated [K(crypt)^+^][NpMI^·–^]

**Figure 4:**
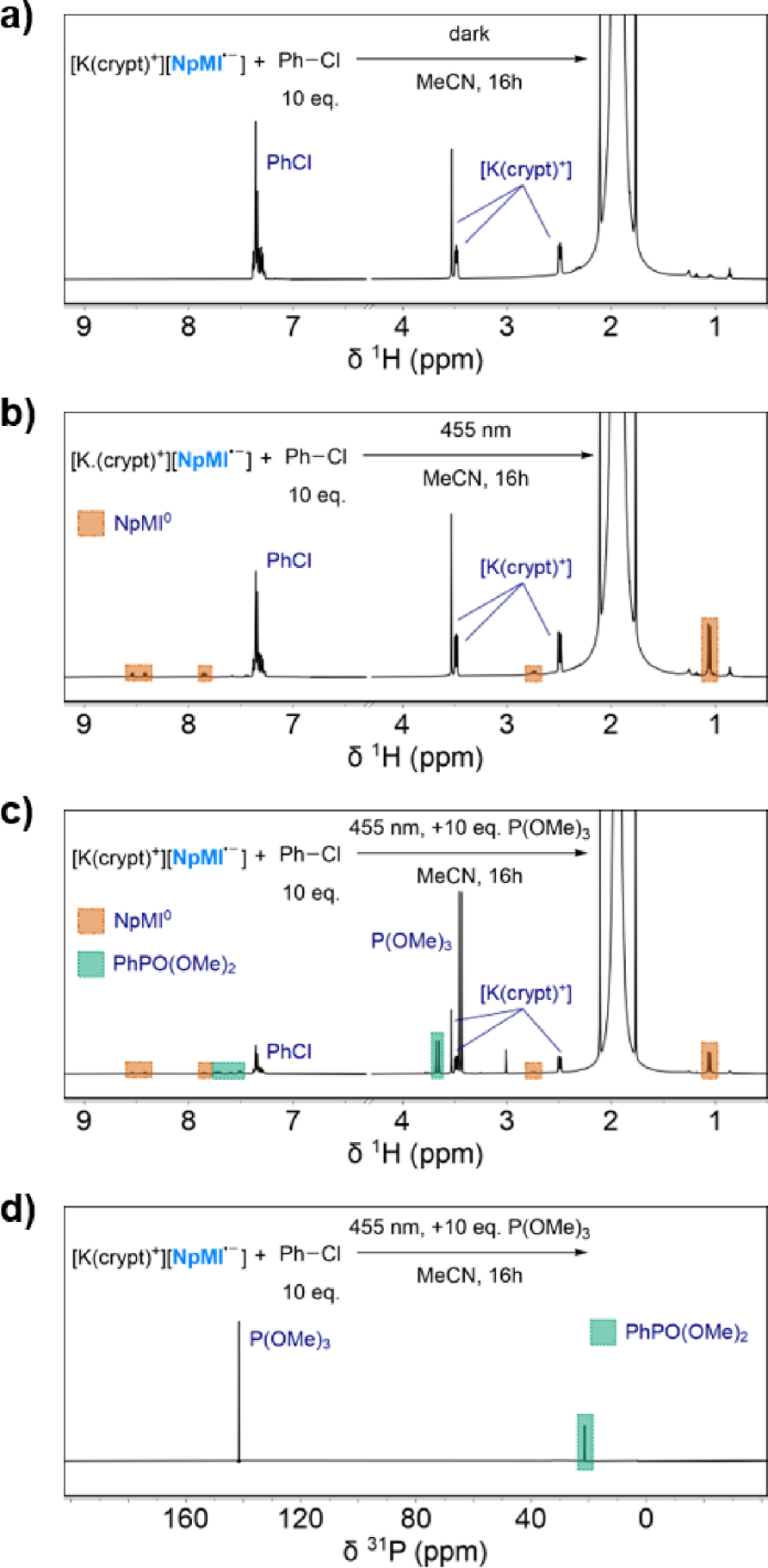
^1^H NMR spectra for the reaction of [K(crypt)^+^][**NpMI^·–^**] with PhCl (10 equiv) in MeCN for 16 h (a) in the dark or (b) under 455 nm LED irradiation; (c) ^1^H and (d) ^31^P{^1^H} NMR spectra for the latter reaction performed in the presence of P(OMe)_3_ (10 equiv). Additional spectra can be found in Section 3 of the SI.

Analogous studies using [K(crypt)^+^][**NpMI^·–^**] in place of [K(crypt)^+^][**DCA^·–^**] yielded mostly very similar results. Again, the salt was found to be stable in the presence of PhCl in the dark (Figure 4a), as well as photostable upon irradiation at 530, 630 or 730 nm for 16 h, either alone or in the presence of P(OMe)_3_. However, unlike [K(crypt)^+^][**DCA^·–^**], the **NpMI**^·–^ salt was found to decompose upon extended irradiation at 455 nm, which is the primary wavelength under which it has previously been proposed to engage in e-PRC reactivity (for full details and additional discussion, see Section 3.1.2 of the SI).^26^

Nevertheless, irradiation at this wavelength in the presence of PhCl led to clear reformation of neutral **NpMI**, which could be observed in the ^1^H NMR spectrum (Figure 4b; *cf.* with [K(crypt)^+^][**DCA^·–^**], *vide supra*). Similar results were also observed at 530 nm (see Section 3.5.2 of the SI). The fate of the Ph fragment could not be clearly ascertained from the same spectra (due to overlap with the much larger resonances for the remaining PhCl starting material), but GC–MS analysis again confirmed formation of PhH (see SI, Section 3.4.1). Analogous irradiation of [K(crypt)^+^][**NpMI^·–^**] in the presence of both PhCl and P(OMe)_3_ again led to formation of PhP(O)(OMe)_2_ (Figure 4c,d). As with [K(crypt)^+^][**DCA^·–^**], superstoichiometric amounts of product formation were achievable (Table 2).

**Table 2:**
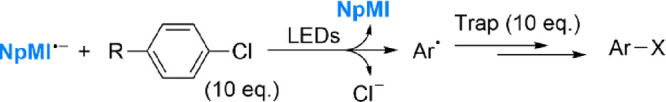
Conversion of ArCl to ArX *via* Reduction to Ar^·^ Using [K(crypt)^+^][NpMI^·–^] and Subsequent Trapping^a^

entry	trap^b^	*t*/h	R	λ/nm	conv./%^c^
1	P(OMe)_3_	16	H	455	157^d^
2	P(OMe)_3_	16	H	530	106
3	P(OMe)_3_	16	H	630	4
4	P(OMe)_3_	16	H	730	0
5	P(OMe)_3_	16	CO_2_Me	455	225^d^
6	P(OMe)_3_	16	CO_2_Me	530	155
7	P(OMe)_3_	16	CO_2_Me	630	159
8	P(OMe)_3_	16	CO_2_Me	730	173
9	P(OMe)_3_	16	OMe	455	195^d^^,^^e^
10	P(OMe)_3_	16	OMe	530	99
11	P(OMe)_3_	16	OMe	630	0
12	P(OMe)_3_	16	OMe	730	0
13	B_2_pin_2_	16	CO_2_Me	455	61
14	B_2_pin_2_	16	CO_2_Me	530	38
15	P(OEt)_3_	16	CO_2_Me	455	177
16	P(OEt)_3_	16	CO_2_Me	530	85

a Reaction between ArCl (10 equiv, 50 mM), trap (10 equiv, 50 mM), and [K(crypt)^+^][**NpMI^·–^**] (1 equiv, 5 mM) in MeCN under LED irradiation (peak wavelength λ).

b For P(OMe)_3_, P(OEt)_3_, and B_2_pin_2_ as traps, X = P(O)(OMe)_2_, P(O)(OEt)_2_, and Bpin, respectively.

c Conversion to ArX measured by quantitative ^31^P{^1^H} (X = P(O)(OMe)_2_, P(O)(OEt)_2_), or ^1^H (X = Bpin) NMR spectroscopy relative to a subsequently added internal standard (Ph_3_PO or mesitylene), relative to the initial amount of [K(crypt)^+^][**NpMI^·–^**] added.

d Average of three experiments (see Section 3.4 of the SI).

e An additional, unidentified signal was also observed at δ(^31^P) = 17.3 ppm, integrating to 16%.

Despite the broad similarities, clear differences in wavelength dependence were also observed between the **NpMI^·–^** and **DCA^·–^** salts. For example, while 530 nm irradiation did not induce significant reduction of PhCl when using **DCA^·–^**, clear conversion was observed at this wavelength using **NpMI^·–^**, which is qualitatively consistent with the increased reducing power expected of the latter (Table 2, entry 2).^9d^ Conversely, no more than a trace reaction was observed at 630 nm (Table 2, entry 3). This contrasts with the generally similar reactivity observed at 530 and 630 nm using **DCA^·–^** and may be due to relatively weak absorption by **NpMI^·–^** around this wavelength. Like with **DCA^·–^**, however, blue light was found to consistently give the highest conversions (e.g., Table 2, entries 5–8). Again, comparative reactions were performed using B_2_pin_2_ (Table 2, entries 13 and 14) and P(OEt)_3_ (Table 2, entries 15 and 16) as alternative radical traps, with the former only achieving stoichiometric conversions.

## Discussion

The results of the above photoreactivity experiments are striking and – while the primary goal of this report is not to advocate for one specific reaction mechanism over another – it has certainly not escaped our notice that they are almost entirely consistent with the originally proposed conPET and e-PRC mechanisms and rather harder to rationalize *via* alternative mechanisms that do not involve SET from *PC^·–^. Indeed, these results present a clear challenge to the idea that such mechanisms must be inherently and fundamentally infeasible, despite recent arguments to this effect.

Nevertheless, we would also emphasize the hidden levels of inherent reaction complexity uncovered by these same experiments, which must also be considered during their interpretation. For example, the observed instability of both **DCA**^·–^ and **NpMI**^·–^ under certain catalytically relevant conditions (presence of Ph^·^, blue LEDs, respectively) has obvious potential mechanistic relevance and means that even if the originally proposed conPET and e-PRC mechanisms are viable in principle, a holistic understanding of these reactions will have to consider the potential (photo)reactivity of these decomposition products as well. Nor would the viability of conPET and e-PRC mechanisms automatically preclude other pathways, such as Nocera’s proposed Meisenheimer mechanism,^21^ from being kinetically competitive in certain reactions.

Similarly, although almost all of the observations made can very easily be rationalized using the original conPET/e-PRC mechanistic model, there are exceptions, such as the unexpected wavelength dependence noted above. For example, the almost ‘binary’ loss of reactivity toward PhCl for **DCA**^·–^ at wavelengths other than 455 nm (see entries 1–3 in Table 1) contrasts with the relatively poor absorption by **DCA**^·–^ at this wavelength. Nor can these initial results yet account for how SET is able to occur, despite the reportedly short *PC^·–^ lifetimes.

These remaining issues can potentially be reconciled through the involvement of higher-order excited states^45^ and/or transient pre-assembly of PC^·–^ and the substrate (though no such preassembly has yet been detected by steady-state spectroscopy: see Section 3.2 of the SI).^16b,46,47^ However, until this is confirmed, alternative mechanisms cannot quite be precluded, and a more detailed, critical discussion of several such mechanisms can be found in Section 4 of the SI.

Filling in these final puzzle pieces will require more detailed studies of the transient absorption behavior, preassembly, and decomposition of PC^·–^, which are beyond the scope of this initial manuscript. Fortunately though, these are all studies that will also be significantly aided and simplified by the accessibility of authentic [K(crypt)^+^][PC^·–^] as isolated materials (and efforts in these directions are already underway). On a broader note, the results reported herein provide a clear proof-of-principle for the viability of isolating PRC-relevant PC^·–^ states of commonly employed PCs. This has the potential to be a much more general strategy for the study of PRC mechanisms and only requires equipment that is widely accessible in synthetic laboratories. However, it has seldom been pursued until now.^4d,8,48^ It has recently been noted of PRC mechanisms that “reaction intermediates formed following the initial PET step [from PC] are seldom monitored and [...] there still remains much to discover about the supposedly ‘dark’ reactions of photocatalytic cycles”.^7e^ As illustrated herein, isolated PC^·–^ represent ideal tools for the study of these missing steps. Thus, while we have focused so far on the investigation of conPET and e-PRC mechanisms, **DCA** in particular has also been employed as a photocatalyst for a much larger variety of ‘standard’ PRC transformations, all of which are proposed to proceed *via***DCA^·–^** as a key intermediate.^49^ Isolation of [K(crypt)^+^][**DCA^·–^**] thus provides a tool compound that can be used to study these reactions as well.

## Conclusions

For the first time, it has been possible to isolate radical anion salts corresponding to the one-electron reduced forms of photocatalysts that have been employed in mechanistically controversial conPET and e-PRC reactions. These can be prepared as authentic materials on a preparative scale and in good yields and their isolation allows for detailed characterization, including by techniques that have not previously been applicable such as XRD. Their availability as ‘bottleable’ compounds has allowed for convenient yet precise and controlled study of their (photo)reactivity, using relatively simple experiments. This in turn has allowed significant new insights into conPET and e-PRC reactions to rapidly be uncovered, including observations of unexpected turnover, counterintuitive wavelength dependence, and previously unreported catalyst (photo)decomposition pathways. These results provide strong, qualitative support for proposed conPET and e-PRC mechanisms, while also highlighting areas where future work is needed to reconcile certain experimental observations.

## Supporting Information Available

Full experimental details for synthesis and characterization of [K(crypt)^+^][**DCA**^·–^] and [K(crypt)^+^][**DCA**^·–^] including copies of NMR, EPR, and UV–vis spectra and XRD data; full experimental details for all photoreactivity studies including LED specifications and quantification of products; additional discussion of alternative photochemical mechanisms (PDF)
CIF file of [K(crypt)^+^][**DCA**^·–^] (CIF)
CIF file of [K(crypt)^+^][**NpMI**^·–^]·(THF) (CIF)

## Supplementary Material

cs3c02515_si_001.pdf

cs3c02515_si_002.cif

cs3c02515_si_003.cif
