# Supplementary material for: Shining Fresh Light on Complex Photoredox Mechanisms through Isolation of Intermediate Radical Anions
Source: ACS Catal. 2023 Jun 30;13(14):9392–403. doi: 10.1021/acscatal.3c02515 (PMC10367049; doi:10.1021/acscatal.3c02515)
Supplement: Supplementary file 1 — cs3c02515_si_001.pdf [file cs3c02515_si_001.pdf]

Supporting Information

Shining Fresh Light on Complex Photoredox Mechanisms Through Isolation of Intermediate Radical Anions

Samuel J. Horsewill,[a] Gabriele Hierlmeier,[b] Zahra Farasat,[c] Joshua P. Barham,[d] Daniel J. Scott*[a]

[a] Department of Chemistry, University of Bath, Claverton Down, Bath, BA2 7AY, United Kingdom

[b] Department of Chemistry, Princeton University, Princeton, New Jersey, 08544, United States of America

[c] Professor Rashidi Laboratory of Organometallic Chemistry, Department of Chemistry, College of Sciences, Shiraz University, Shiraz, 71467-13565, Iran

[d] Institute of Organic Chemistry, University of Regensburg, Universitätsstr. 31, 93053 Regensburg, Germany

*E-mail: ds2630@bath.ac.uk

Contents

1. General experimental methods 3
   1.1. Description of LED apparatus 4
   1.2. Calibration for ^{31}P NMR integration 6
2. Synthesis and characterisation of $[K(crypt)^+][PC^{•−}]$ 7
   2.1. Synthesis and characterization of $[K(crypt)^+][DCA^{•−}]$ 7
   2.2. Synthesis and characterization of $[K(crypt)^+][NpMI^{•−}]$ 10
3. Photoreactivity studies 13
   3.1. Control experiments ($[K(crypt)^+][PC^{•−}]$ photostability) 13
      3.1.1. Photostability of $[K(crypt)^+][DCA^{•−}]$ 14
      3.1.2. Photostability of $[K(crypt)^+][NpMI^{•−}]$ 17
   3.2. UV-vis spectroscopy of $[K(crypt)^+][PC^{•−}]$ in the presence of substrate 22
   3.3. Reactivity of $[K(crypt)^+][PC^{•−}]$ in the dark 24
   3.4. Reactivity of $[K(crypt)^+][PC^{•−}]$ under LED irradiation 25
      3.4.1. GC-MS analysis of reactions of $[K(crypt)^+][PC^{•−}]$ with PhCl 26
      3.4.2. Reactivity of $[K(crypt)^+][DCA^{•−}]$ under LED irradiation 27
      3.4.3. Reactivity of $[K(crypt)^+][NpMI^{•−}]$ under LED irradiation 32
      3.4.4. Photoreactivity at lower $[K(crypt)^+][PC^{•−}]$ concentration 36
   3.5 Quantification of remaining PC 37
      3.5.1 Quantification of remaining DCA^0 37
      3.5.2 Quantification of remaining $NpMI^0$ 39
4. Supplementary mechanistic discussion 45
   4.1. Possible formation of $[NpMI•H^-]$ or other closed-shell species prior to substrate reduction 45
   4.2. Possible formation of other closed-shell species after initial substrate reduction 46
   4.3. Possible oxidation of $PC^{•−}$ by O_2 47
   4.4. Possible substrate/$PC^{•−}$ pre-assembly 49
5. X-ray crystallographic details 50
   5.1. XRD data for $[K(crypt)^+][DCA^{•−}]$ 50
   5.2. XRD data for $[K(crypt)^+][NpMI^{•−}]•(THF)$ 55
6. References for supporting information 61

1. General experimental methods

Unless stated otherwise, all reactions, manipulations and spectroscopic acquisitions were performed under an N_2 or Ar atmosphere (< 0.1 ppm O_2, H_2O) through use of MBraun Labmaster gloveboxes and standard Schlenk line techniques. All glassware was oven-dried (>160 °C) overnight prior to use.

MeCN, PhMe, Et_2O and hexane were purified using an MBraun SPS-800 system, degassed, and stored over molecular sieves (3 Å). THF was pre-dried over activated molecular sieves (3 Å) in a Winchester, then dried in a sodium/benzophenone solvent still, before being degassed and stored over molecular sieves (3 Å). d_8-THF was dried by refluxing over K, then degassed, distilled and stored over molecular sieves (3 Å). CD_3CN was dried by refluxing over CaH_2, degassed, distilled, and stored over molecular sieves (3 Å). NpMI[1] and KC_8[2] were prepared in accordance with the literature. All other materials were purchased from major suppliers. Unless noted otherwise, solids were dried under vacuum and liquids were degassed and dried over molecular sieves (3 Å) unless already supplied under inert atmosphere.

Unless noted otherwise, NMR spectra were recorded at room temperature on Bruker AVIII HD 400 nanobay or Bruker Avance 400 (400 MHz) spectrometers. Chemical shifts, δ, are reported in parts per million (ppm); ^{1}H NMR and ^{13}C NMR shifts are reported relative to $SiMe_4$ and were referenced internally to residual solvent peaks, while ^{31}P NMR shifts were referenced externally to 85 % H_3PO_4 (aq.) and ^{11}B NMR shifts were referenced externally to 15% $BF_3.OEt_2$ in $CDCl_3$. The abbreviations s, d, t, q, m are used to indicate singlets, doublets, triplets, quartets and multiplets, respectively. Except where indicated otherwise, integrals for ^{31}P{^{1}H} and ^{31}P spectra are provided for the purposes of qualitative comparison only and should not be considered quantitatively accurate. Quantitative ^{31}P{^{1}H} NMR spectra were acquired using an inverse gated proton decoupled experiment, and the validity of integration was confirmed using solutions of known concentration (see section 1.2).

EPR spectra were recorded at room temperature on a Bruker EMXmicro X-band continuous wave EPR spectrometer. UV-vis spectra were recorded at room temperature on a Horiba Duetta combination Fluorescence and Absorbance spectrometer or on an Ocean Optics assembly with an Ocean Optics DH2000 light source and Ocean Insight Flame miniature spectrometer. Gas chromatography was performed on an Agilent 8890 GC system with an integrated Agilent 5977B mass spectrometer, with the assistance of Dr Kathryn Proctor at the University of Bath.[3] Elemental analysis was performed by Orla McCullough at London Metropolitan University, with each measurement performed in duplicate and provided alongside a separately measured standard (acetanilide).

1.1. Description of LED apparatus

Photochemical reactions were performed using the experimental set-up shown in Figure S1. This consists of individual LEDs mounted in series to a heatsink plate, and the six-LED array is powered by a 28 W power supply. On top of the heatsink is placed a hollow block through which cooling water can be circulated. Cut into this block are openings that allow reaction vessels to be individually placed above each LED. The entire apparatus can be placed on top of a stirrer plate if required, and for safety must be covered by an opaque box screen before the LEDs can be activated.

Suitable reaction vessels include flat-bottomed Schlenk tubes. However, for this study, to ensure the most rigorous possible inert atmosphere, J. Youngs inert atmosphere NMR tubes were placed into suitably sized vials, the latter of which were wrapped in cylinders of Al foil (to maximise LED light capture) and filled with deionised water (for thermal contact with the cooling block). The narrowness of these vessels was also intended to aid with light penetration.

The LEDs employed for this study were:

Osram OSLON SSL 120, Deep Blue (455 nm)
Osram OSLON SSL 120, Green (530 nm)
Cree High Power, Red (630 nm)
Luminus SST-10-FR-B130, Far Red (730 nm)

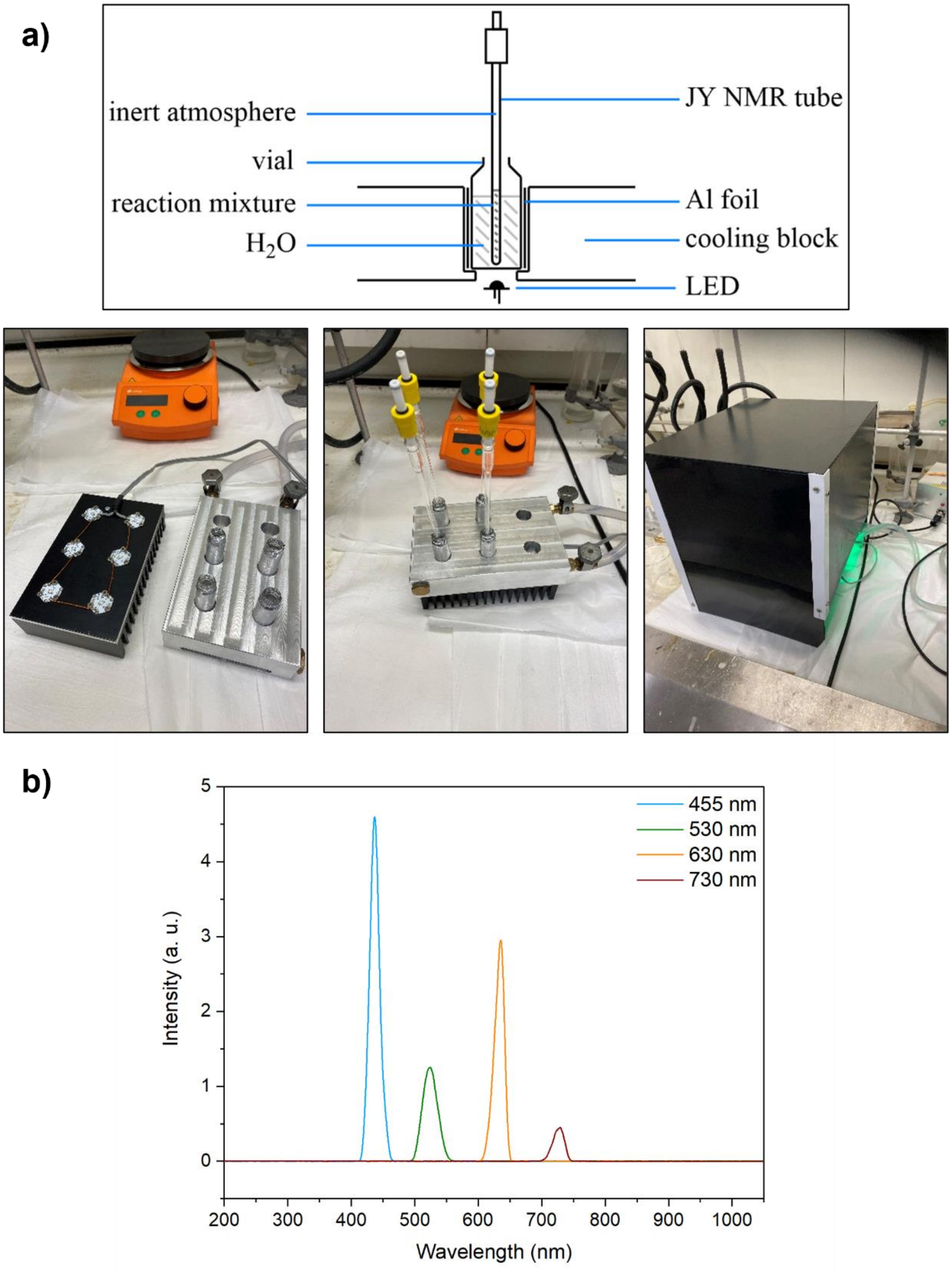

Figure S1. (a) Illustration and photographs of the apparatus used for photochemical experiments. Similar apparatus has been described previously by the Wolf group.[4] Illustration not to scale. (b) Output spectra of the LEDs used in this study, as recorded by holding each LED at a fixed distance of 73 cm from the fibre-optic input of an Ocean Insight Flame miniature spectrometer (3 ms exposure time).

1.2. Calibration for ^{31}P NMR integration

Three J. Youngs NMR tubes were each charged with 1:1 mixtures of $PhPO(OMe)_2$ and Ph_3PO, both at 5 mM in MeCN (the concentrations expected to be found in the reaction mixtures described below). The ^{31}P NMR spectra of the three solutions were then recorded using inverse-gated proton decoupled experiments with an extended, 1 s delay between scans, and the ratios of the two resonances were determined by integration. The ratio in each case was found to be in satisfactory agreement with the expected 1:1, within the expected error for such measurements (Ph_3PO:$PhPO(OMe)_2$ = 1:0.94, 1:0.95, 1:0.95).

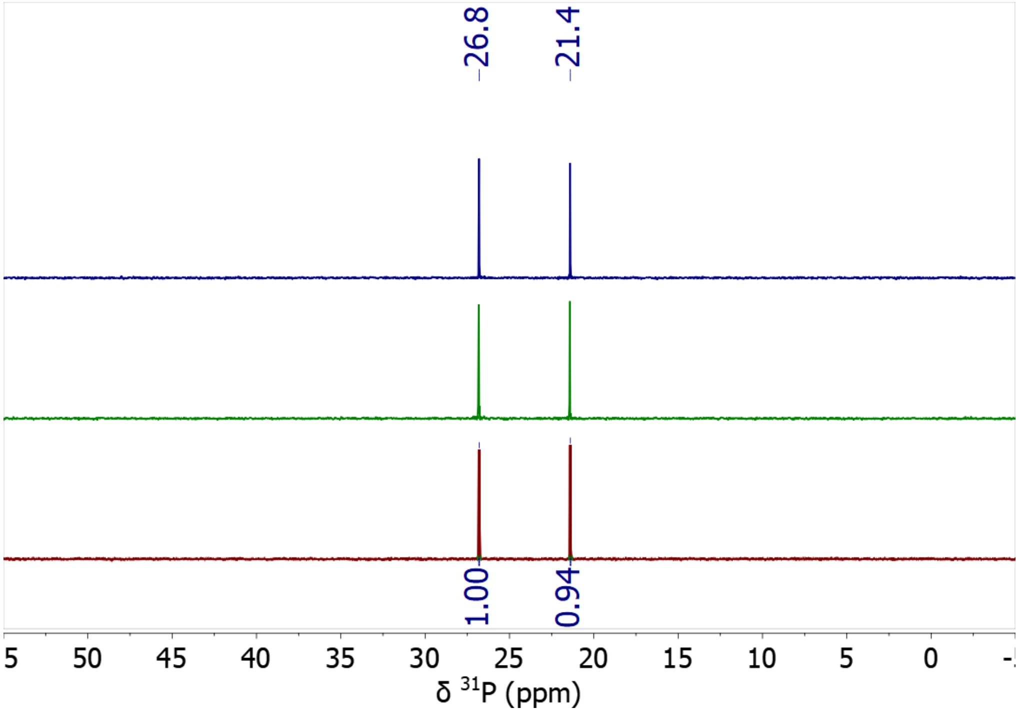

Figure S2. Quantitative measurement of the ^{31}P NMR spectrum in triplicate of a 1:1 mixture of Ph_3PO (26.8 ppm) and $PhPO(OMe)_2$ (21.4 ppm) in MeCN solution.

2. Synthesis and characterisation of [K(crypt)+][PC•−]

2.1. Synthesis and characterization of [K(crypt)+][DCA•−]

A dry mixture of **DCA** (343 mg, 1.50 mmol), KC_8 (231 mg, 1.71 mmol) and 2,2,2-cryptand (564 mg, 1.50 mmol) was added to a Schlenk tube. While stirring, THF (50 mL) was added, and a colour change to deep purple was immediately observed. The suspension was stirred for 1.5 h, then filtered *via* filter cannula. The dark residual solids were extracted with THF (2x 15 mL) until the extracts were pale, and the combined filtrates were again filtered *via* filter cannula to remove any final traces of graphite. The THF was removed from the filtrate *in vacuo* to leave a dark solid. This was washed with toluene (3 x 15 mL) and hexane (3 x 15 mL), then dried *in vacuo* to give [K(crypt)+][**DCA**•−] as a dark purple solid (849 mg, 1.32 mmol, 88%).

Anal. Calcd. (%) $C_{34}H_{44}KN_4O_6$: C, 63.43; H, 6.89; N, 8.70. Found: C, 63.08-63.22; H, 6.94-6.95; N, 8.46-8.46.

^{1}H NMR (CD_3CN): δ = 3.55 (12H, s), 3.51 (12H, t, $^3J(^1H-^1H)$ = 4.2 Hz), 2.51 ppm (12H, t, $^3J(^1H-^1H)$ = 4.3 Hz).

$^{13}C\{^1H\}$ NMR (CD_3CN): δ = 71.9 (s), 69.1 (s), 55.2 ppm (s).

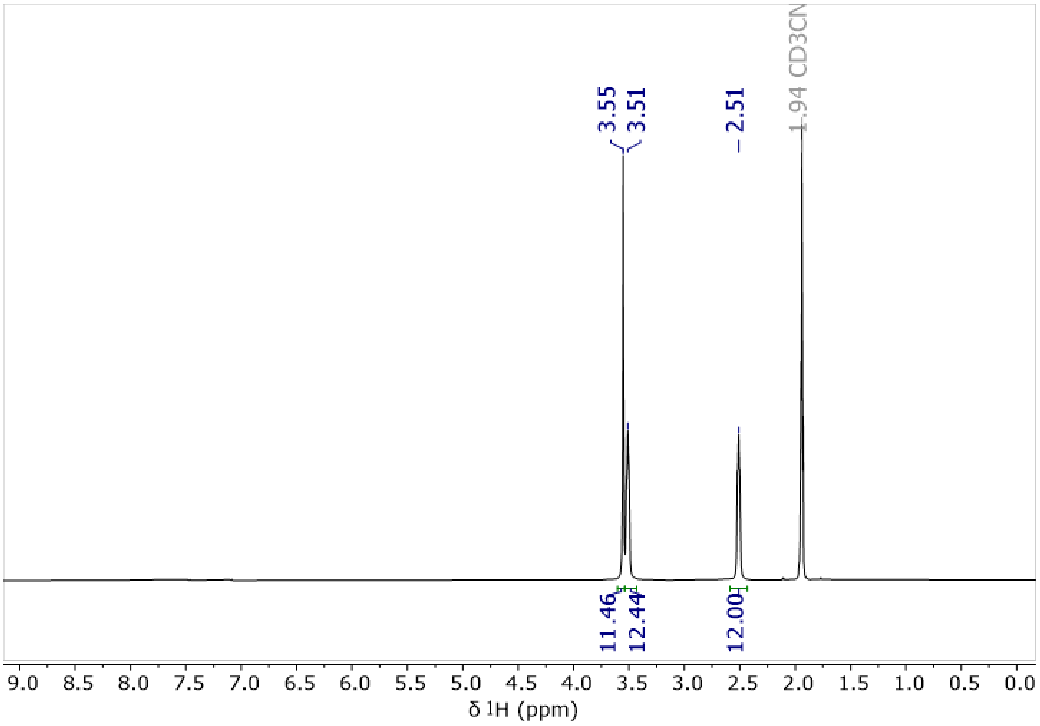

Figure S3. ^{1}H NMR spectrum of [K(crypt)+][**DCA**•−] in CD_3CN.

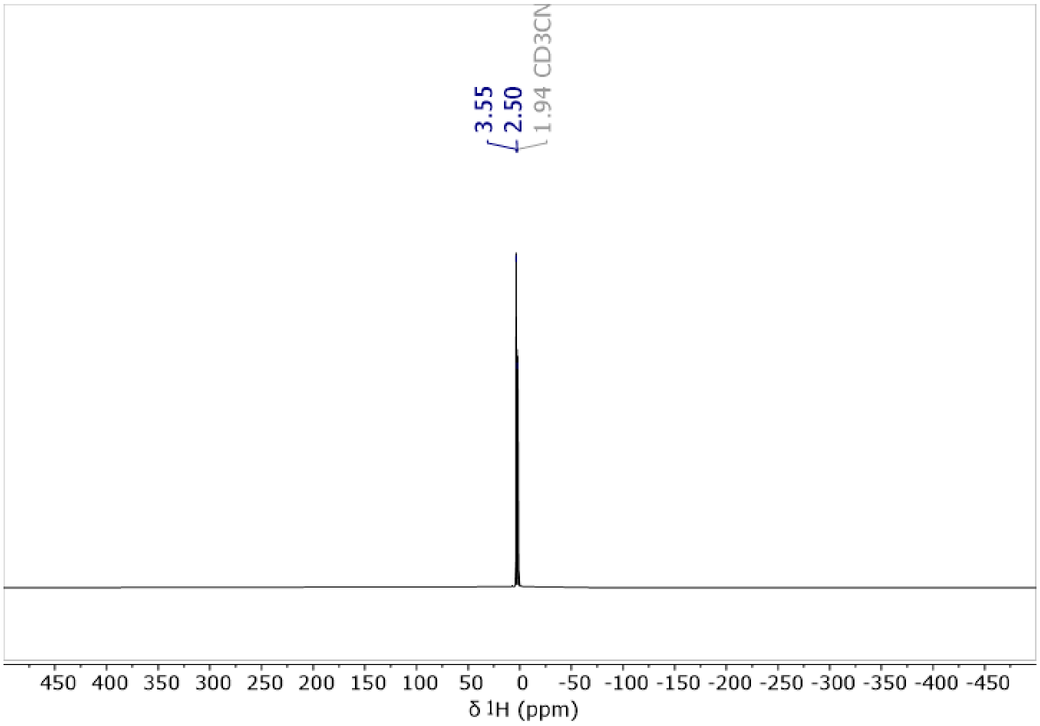

Figure S4. Broader-window ${}^{1}H$ NMR spectrum of [K(crypt)$^{+}$][**DCA**$^{\bullet-}$] in CD_3CN.

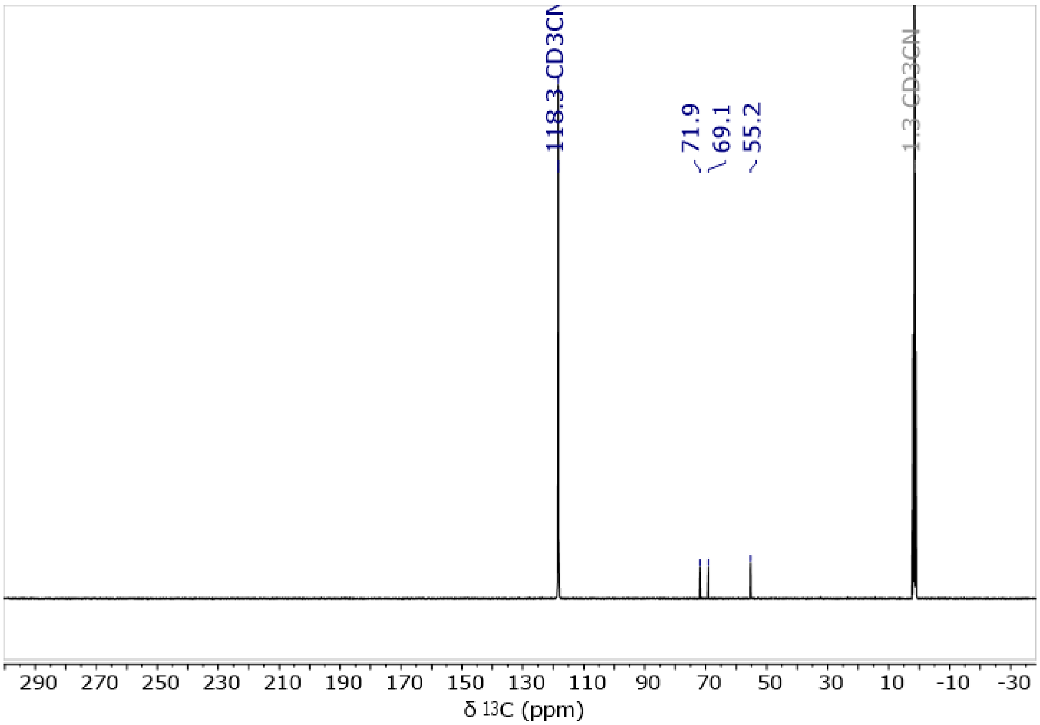

Figure S5. ${}^{13}C\{{}^{1}H\}$ NMR spectrum of [K(crypt)$^{+}$][**DCA**$^{\bullet-}$] in CD_3CN.

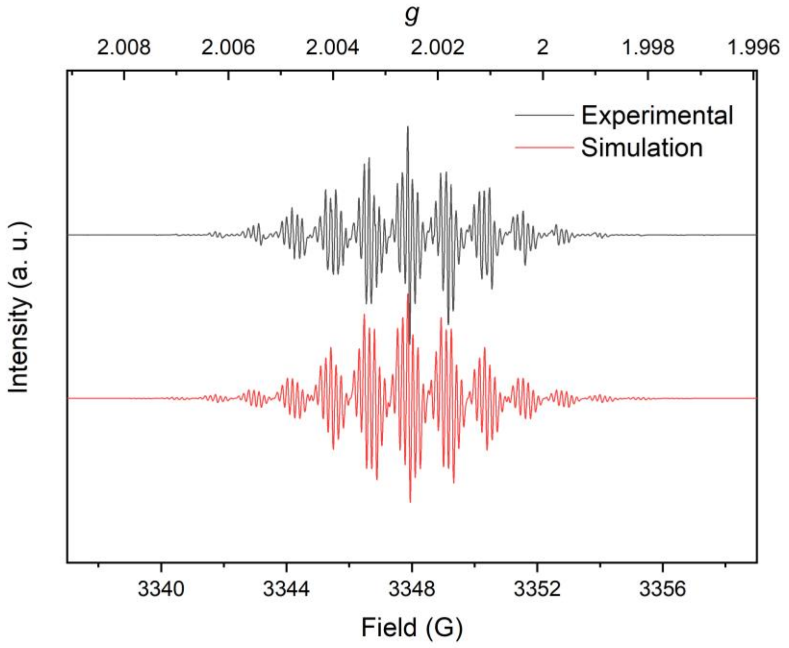

Figure S6. X-band EPR spectrum of [K(crypt)$^+$][**DCA$^{\bullet-}$**] in 4:1 PhMe/THF (109 μM). Microwave frequency 9.3836 MHz, power 0.080 mW. Simulated as two components with g = 2.00256, 4 x A(^{1}H) = 3.904, 4 x A(^{1}H) = 2.969, 2 x A(^{14}N) = 0.4307 MHz. 15%: A(^{13}C) = 20.631 MHz.

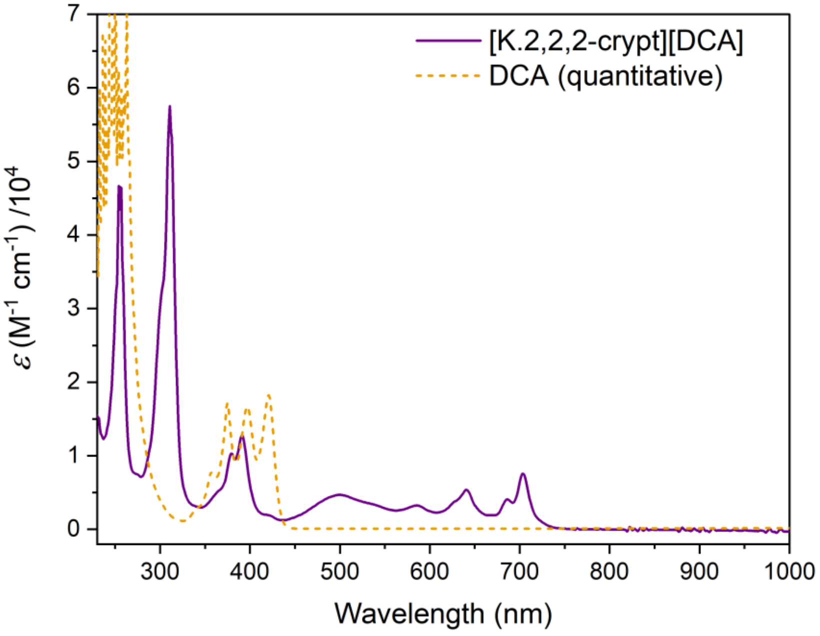

Figure S7. Electronic absorption spectrum of [K(crypt)$^+$][**DCA$^{\bullet-}$**] in MeCN (544 μM, 1 mm path). Neutral **DCA** also shown for ease of comparison.

2.2. Synthesis and characterization of [K(crypt)+][NpMI•−]

To a Schlenk tube were added **NpMI** (345 mg, 0.965 mmol), KC_8 (143 mg, 1.058 mmol) and 2,2,2-cryptand (353 mg, 0.938 mmol). While stirring, THF (30 mL) was added. A colour change from white and brown solids to a dark green suspension was immediately observed. The mixture was stirred for 1 h, then the suspension was filtered *via* filter cannula to obtain a dark green solution, leaving behind dark solids. These solids were extracted with THF (3 x 15 mL) until the extracts were pale, and the filtrates were combined. The solution was filtered once more and the THF was removed from the solution *in vacuo* to leave a dark green solid, which was washed with toluene (3 x 10 mL) and hexane (3 x 10 mL) and dried *in vacuo* to furnish [K(crypt)+][**NpMI•−**] as a green powder (633 mg, 0.819 mmol, 85% yield).

Anal. Calcd. (%) $C_{42}H_{59}KN_3O_8$: C, 65.26; H, 7.69; N, 5.44. Found: C, 65.56-65.77; H, 7.73-7.77; N, 5.22-5.26.

^{1}H NMR (CD_3CN): δ = 3.55 (12H, s), 3.51 (12H, t, ^3J(^{1}H-^{1}H) = 4.1 Hz), 2.51 (12H, t, ^3J(^{1}H-^{1}H) = 4.1 Hz), 1.39 (12H, br s), 1.07 ppm (2H, m).

^{13}C{^{1}H} NMR (CD_3CN): δ = 71.3 (s), 68.5 (s), 54.7 ppm (s).

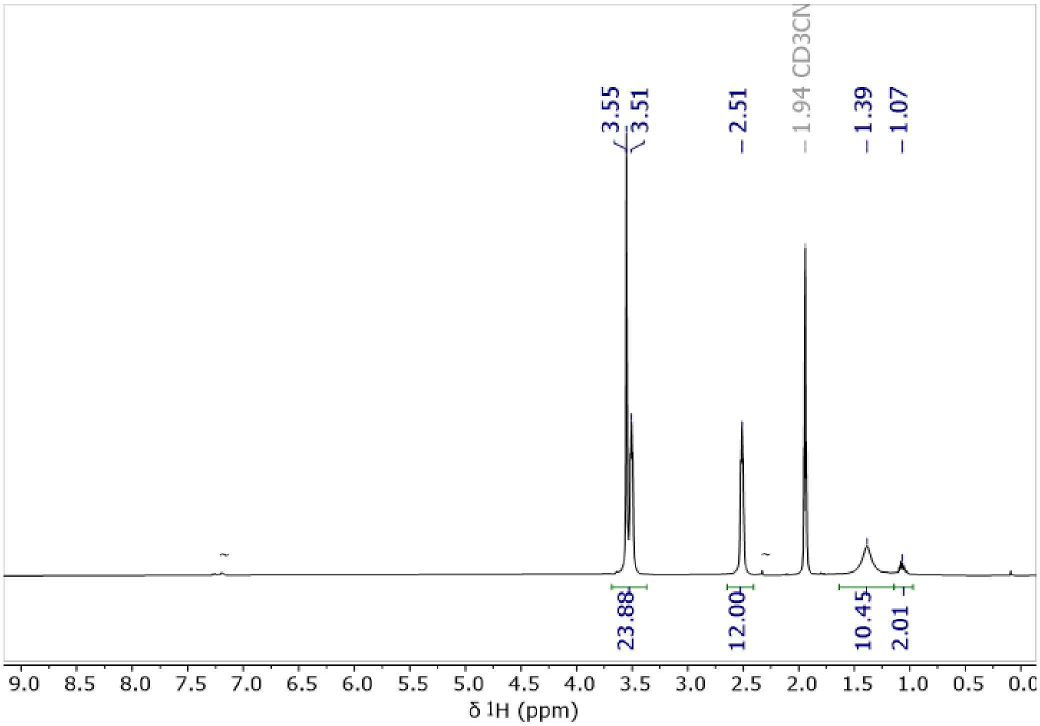

Figure S8. ^{1}H NMR spectrum of [K(crypt)+][**NpMI•−**] in CD_3CN. Resonances labelled with '~' are assigned to trace residual toluene.

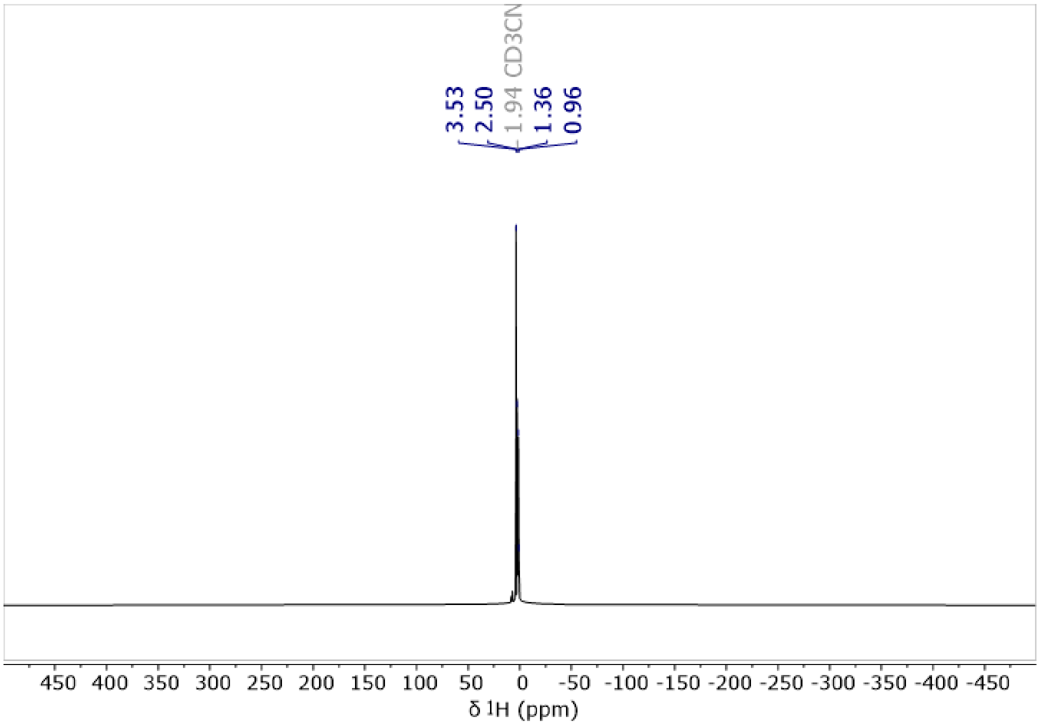

Figure S9. Broader-window ^{1}H NMR spectrum of [K(crypt)$^{+}$][**NpMI$^{\bullet-}$**] in CD_3CN.

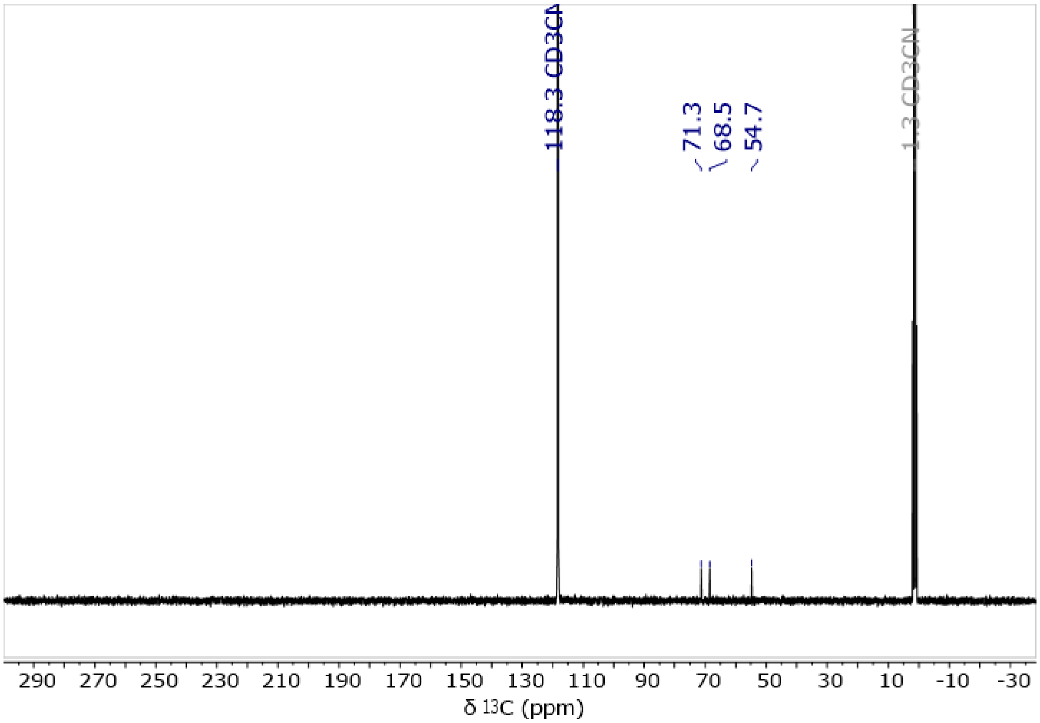

Figure S10. $^{13}C\{^{1}H\}$ NMR spectrum of [K(crypt)$^{+}$][**NpMI$^{\bullet-}$**] in CD_3CN.

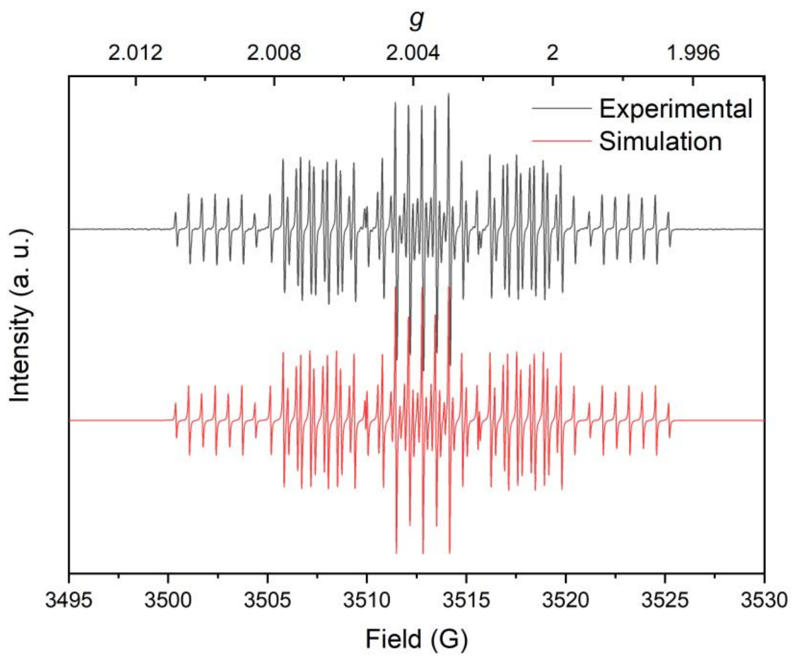

Figure S11. X-band EPR spectrum of [K(crypt)$^+$][**NpMI**$^{\bullet -}$] in THF (199 μM). Microwave frequency 9.8516 MHz, power 0.10 mW. Simulated as g = 2.00371, 2 x $A(^1H)$ = 15.833, 2 x $A(^1H)$ = 13.357, 2 x $A(^1H)$ = 1.833, 1 x $A(^{14}N)$ = 3.760 MHz.

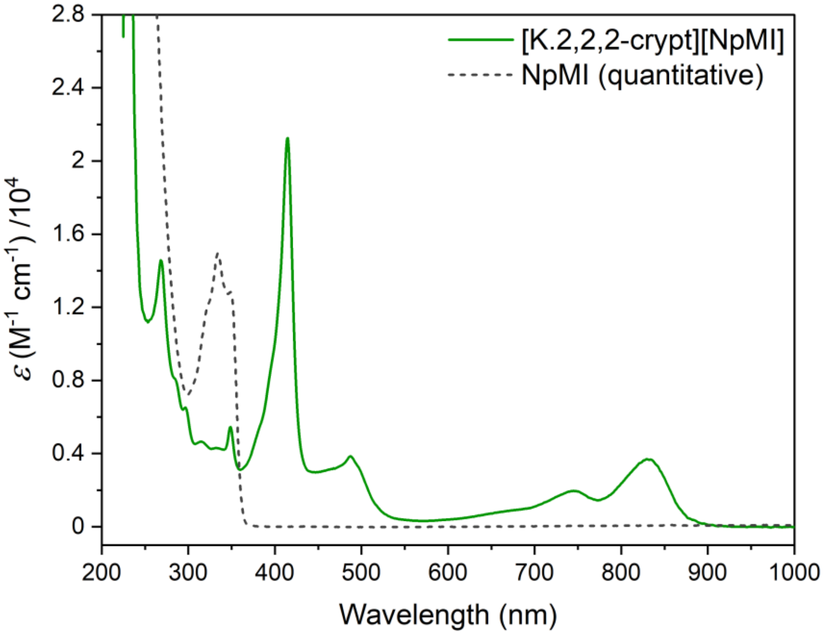

Figure S12. Electronic absorption spectrum of [K(crypt)$^+$][**NpMI**$^{\bullet -}$] in MeCN (504 μM, 1mm path). Neutral **NpMI** also shown for ease of comparison.

3. Photoreactivity studies

Unless otherwise noted, every reaction was performed in a J Youngs tap NMR tube in MeCN at 5 mM $PC^{\bullet-}$, 50 mM Ar-Cl and 50 mM radical trap ($P(OMe)_3$, $P(OEt)_3$ or B_2pin_2) concentrations as appropriate. After the reaction was complete, for quantification 1 eq. of either Ph_3PO (for $P(OR)_3$-trapped reactions, $\delta(^{31}P)$ = 26.7 (1P) ppm) or mesitylene (for B_2pin_2-trapped reactions, $\delta(^{1}H)$ = 6.77 (3H), 2.21 (9H) ppm) was added with respect to the original $PC^{\bullet-}$ concentration. Integrals were calculated in MestReNova using a linear correction.

3.1. Control experiments ([K(crypt)$^+$][PC$^{\bullet-}$] photostability)

Solutions of [K(crypt)$^+$][PC$^{\bullet-}$] were irradiated under a range of different wavelengths, both with and without the presence of $P(OMe)_3$, to confirm their photostability under our reaction conditions. No major reaction products were identified by NMR spectroscopy except for [K(crypt)$^+$][**NpMI**$^{\bullet-}$] solutions after irradiation by light of 455 nm, where a colour change from green to pink/red was also noted. The UV-vis absorption spectra of [K(crypt)$^+$][PC$^{\bullet-}$] were also recorded following irradiation by light of each wavelength for 16 h. No significant change from the starting material was observed for any $PC^{\bullet-}$/wavelength combination, except for [K(crypt)$^+$][**NpMI**$^{\bullet-}$] with 455 nm irradiation.

3.1.1. Photostability of [K(crypt)+][DCA•−]

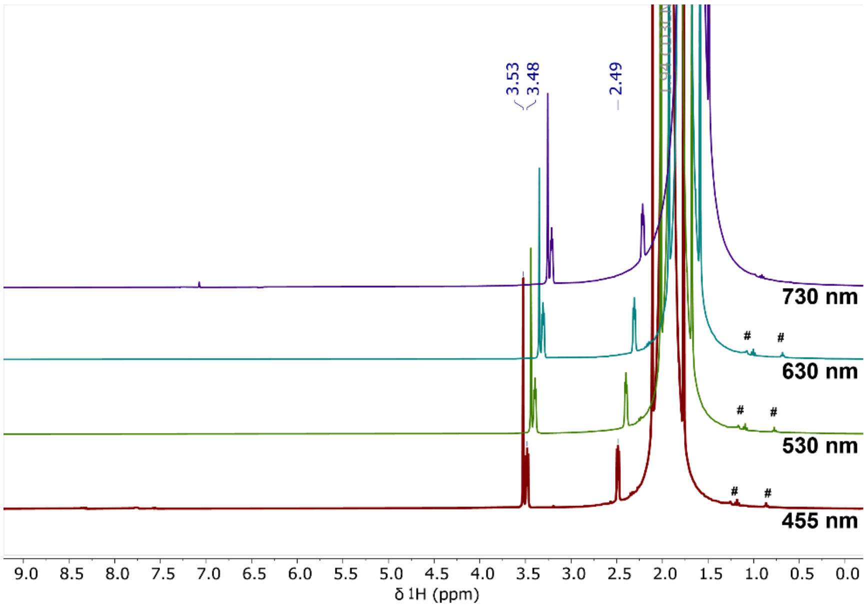

Figure S13. ^{1}H NMR spectra of [K(crypt)+][**DCA**•−] after irradiation at four different wavelengths for 16h. Spectra are offset by 0.11 ppm for clarity. The labelled chemical shifts correspond to [K(crypt)+]. Adventitious hexane is labelled with #.

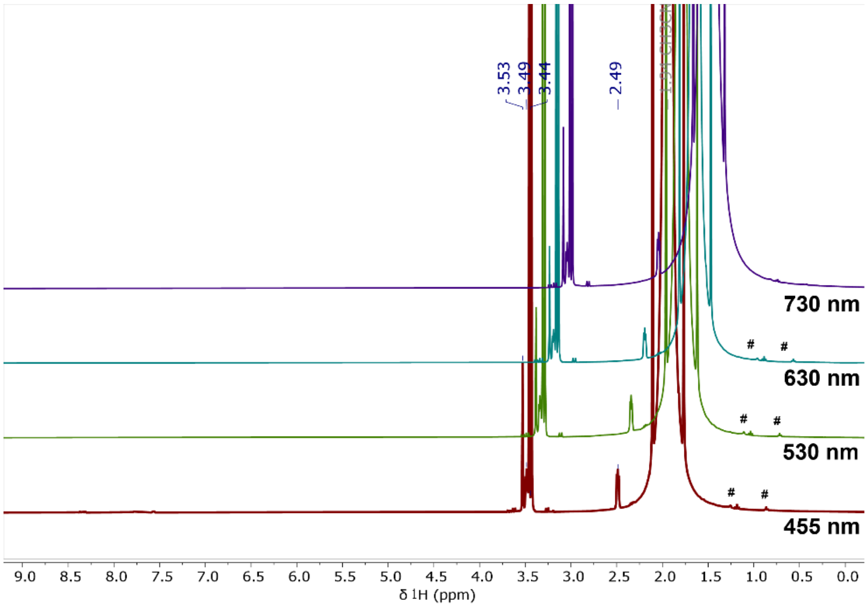

Figure S14. ^{1}H NMR spectra of mixtures of [K(crypt)+][**DCA**•−] and $P(OMe)_3$, following irradiation at four different wavelengths for 16h. The spectra are each offset by 0.16 ppm for clarity. The labelled chemical shifts correspond to $P(OMe)_3$ (3.44 ppm) and [K(crypt)+]. Adventitious hexane is labelled with #.

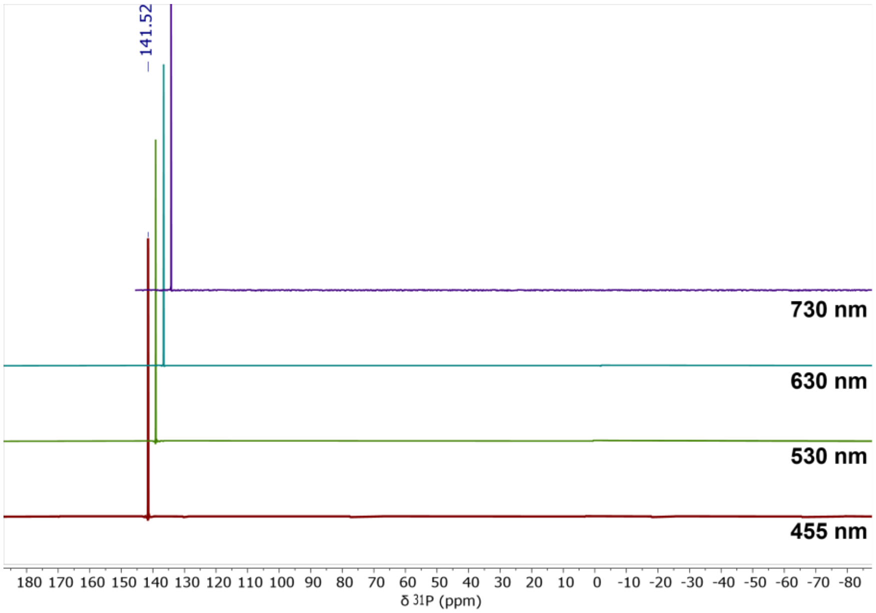

Figure S15. $^{31}P\{^1H\}$ NMR spectra of mixtures of [K(crypt)$^+$][**DCA**$^{\bullet-}$] and $P(OMe)_3$, following irradiation at four different wavelengths for 16h. The spectra are each offset by 1.3 ppm for clarity. The sole resonance observed corresponds to $P(OMe)_3$.

For completeness, the photostability of [K(crypt)$^+$][**DCA**$^{\bullet-}$] in the presence of B_2pin_2 under 455 nm irradiation was also established by NMR spectroscopy, which revealed no changes after 16 h.

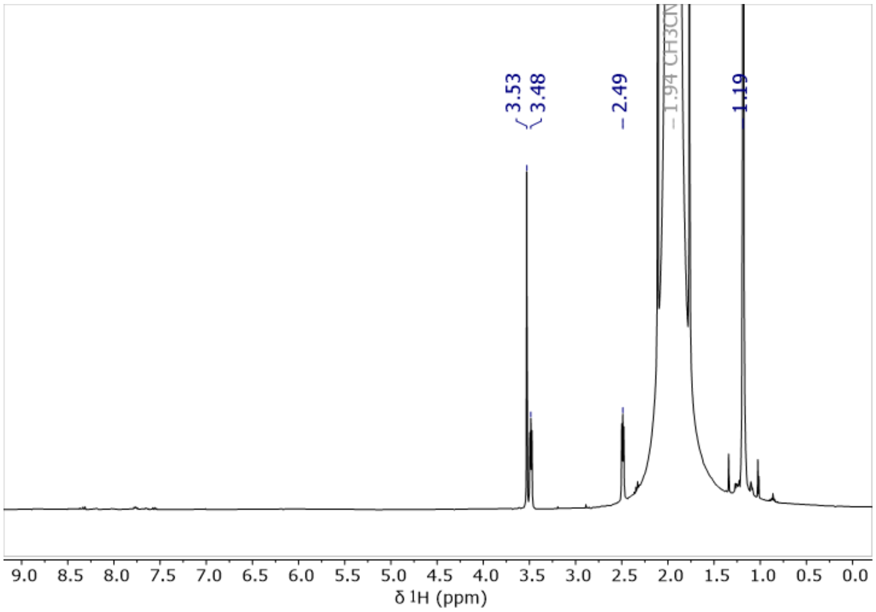

Figure S16. ^1H NMR spectrum of a mixture of [K(crypt)$^+$][**DCA**$^{\bullet-}$] and B_2pin_2, following irradiation at 455 nm for 16h. The labelled chemical shifts correspond to B_2pin_2 (1.19 ppm) and [K(crypt)$^+$].

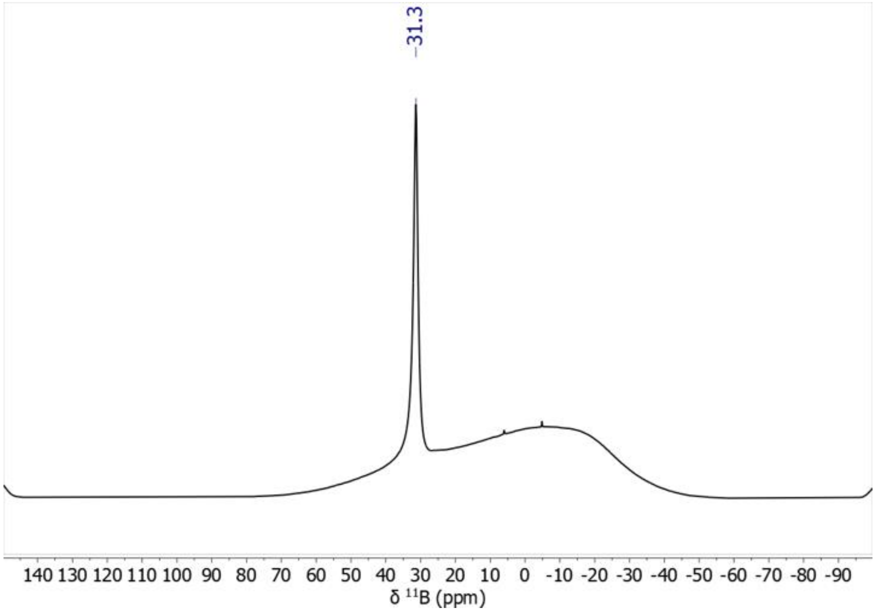

Figure S17. $^{11}B\{^{1}H\}$ NMR spectrum of a mixture of [K(crypt)$^{+}$][**DCA$^{\bullet-}$**] and B_2pin_2, following irradiation at 455 nm for 16h. The labelled resonance corresponds to B_2pin_2.

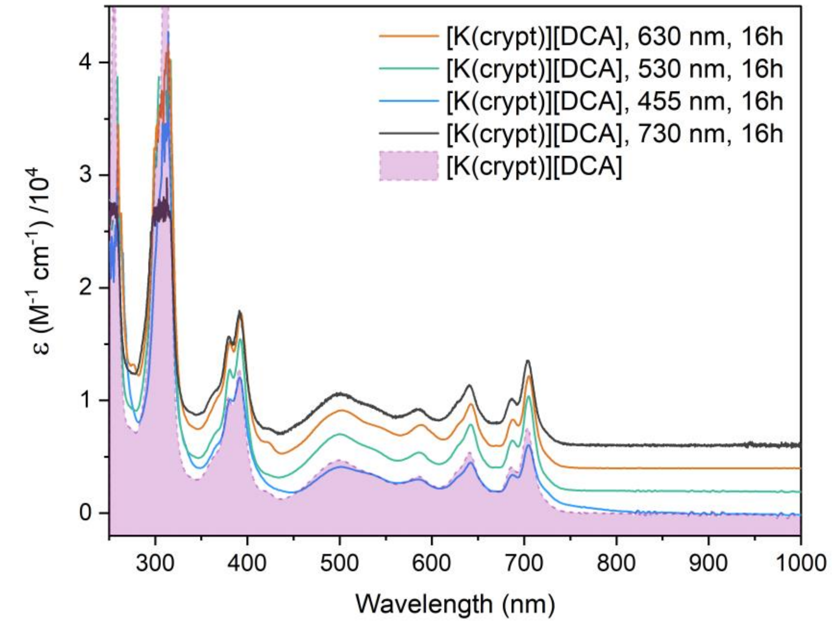

Figure S18. UV-vis absorption spectra of [K(crypt)$^{+}$][**DCA$^{\bullet-}$**], following irradiation by light of various wavelengths for 16 h. The ε data is offset vertically by 2 x10^3 (530 nm), 4 x10^3 (630 nm) or 6 x10^3 M^{-1} cm^{-1} (730 nm) for clarity. Irradiation was performed at 5 mM concentration in MeCN, and the resulting solutions were then diluted to 1 mM concentration in the same solvent for recording in a 1 mm path length cuvette. The spectrum of [K(crypt)$^{+}$][**DCA$^{\bullet-}$**] is included for comparison.

3.1.2. Photostability of [K(crypt)+][NpMI•−]

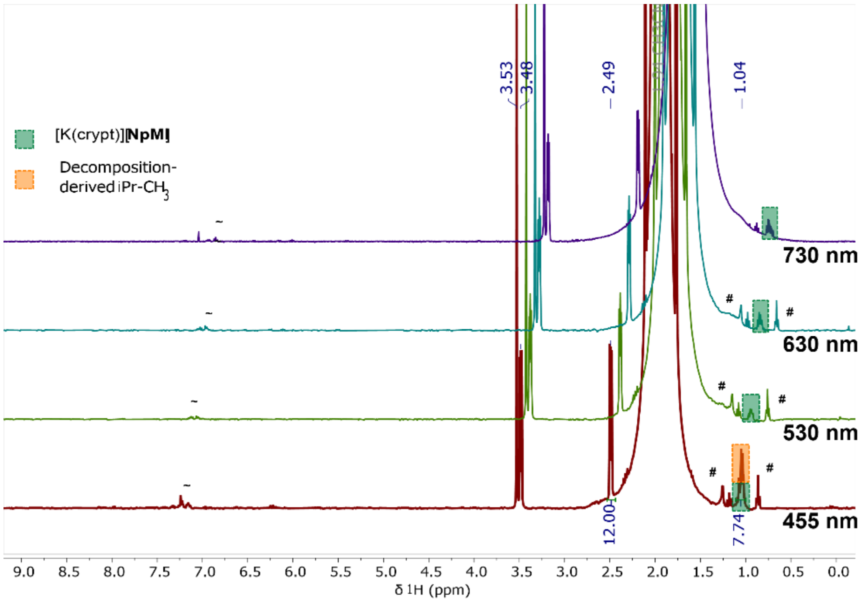

Figure S19. ^{1}H NMR spectra of [K(crypt)+][**NpMI•−**] after irradiation at four different wavelengths for 16 h. The spectra are each offset by 0.11 ppm for clarity. Labelled resonances are assigned to [K(crypt)+]. Resonances indicated with ~ are assigned to small quantities of toluene, and those indicated with # to hexane, adventitiously present in the reaction solvent. Indicated in orange is a resonance attributed to photoinduced decomposition of [**NpMI•−**].

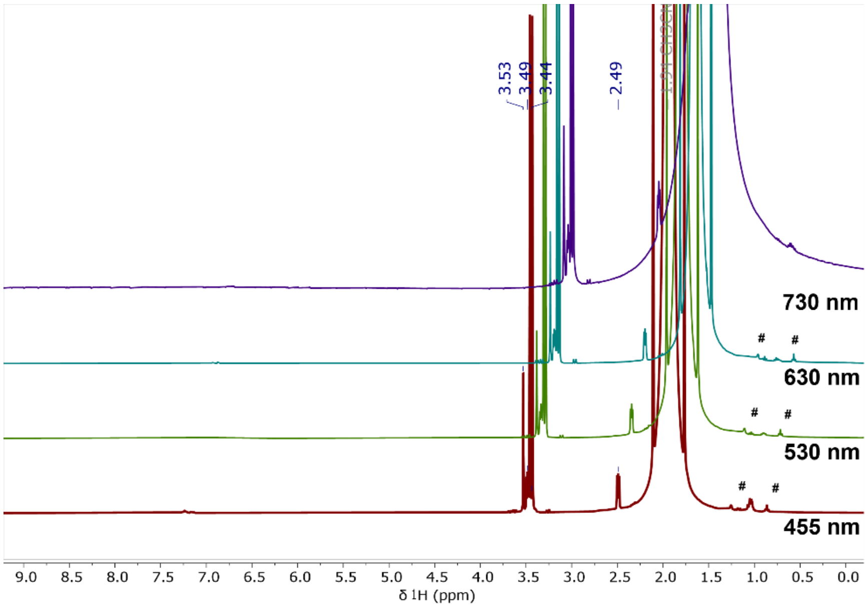

Figure S20. ^{1}H NMR spectra of mixtures of [K(crypt)$^{+}$][**NpMI$^{\bullet-}$**] and $P(OMe)_3$, following irradiation at four different wavelengths for 16h. The spectra are each offset by 0.15 ppm for clarity. The labelled chemical shifts correspond to $P(OMe)_3$ (3.44 ppm) and [K(crypt)$^{+}$]. Adventitious hexane is labelled with #.

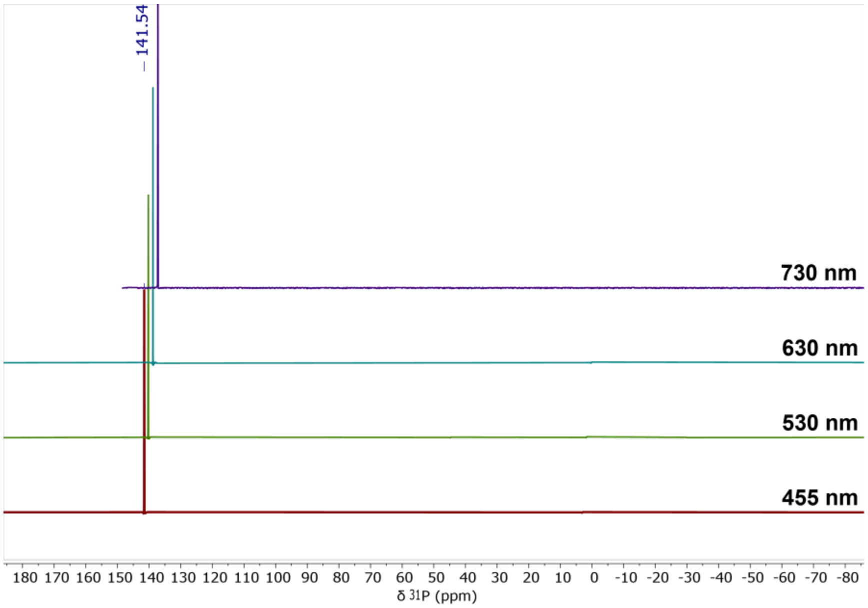

Figure S21. $^{31}P\{^{1}H\}$ NMR spectra of mixtures of [K(crypt)$^{+}$][**NpMI$^{\bullet-}$**] and $P(OMe)_3$, following irradiation at four different wavelengths for 16h. The spectra are each offset by 1.3 ppm for clarity. The sole resonance observed corresponds to $P(OMe)_3$.

For completeness, the photostability of [K(crypt)$^+$][**NpMI**$^{•-}$] in the presence of B_2pin_2 under 455 and 530 nm irradiation was also established by NMR spectroscopy, which revealed no changes after 16 h.

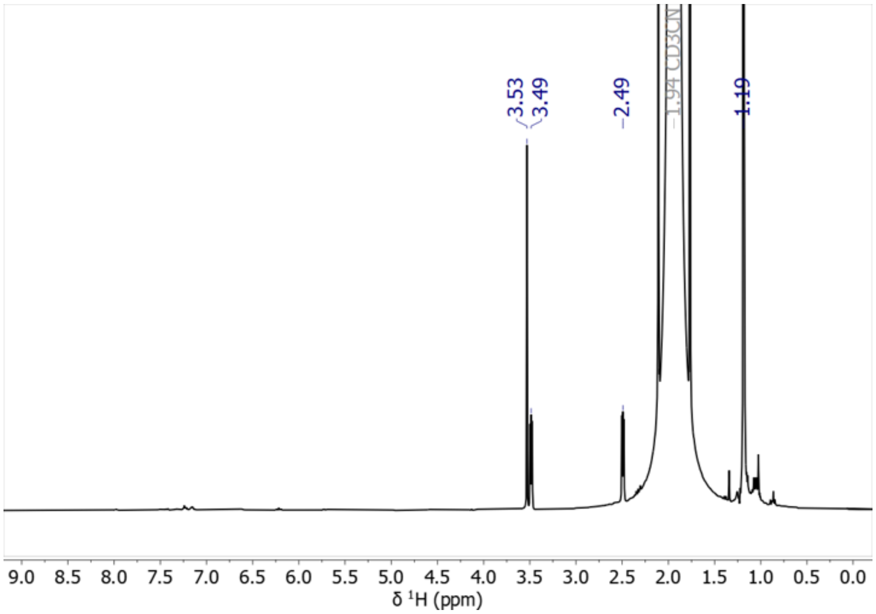

Figure S22. ^{1}H NMR spectrum of a mixture of [K(crypt)$^+$][**NpMI**$^{•-}$] and B_2pin_2, following irradiation at 455 nm for 16h. The labelled chemical shifts correspond to B_2pin_2 (1.19 ppm) and [K(crypt)$^+$].

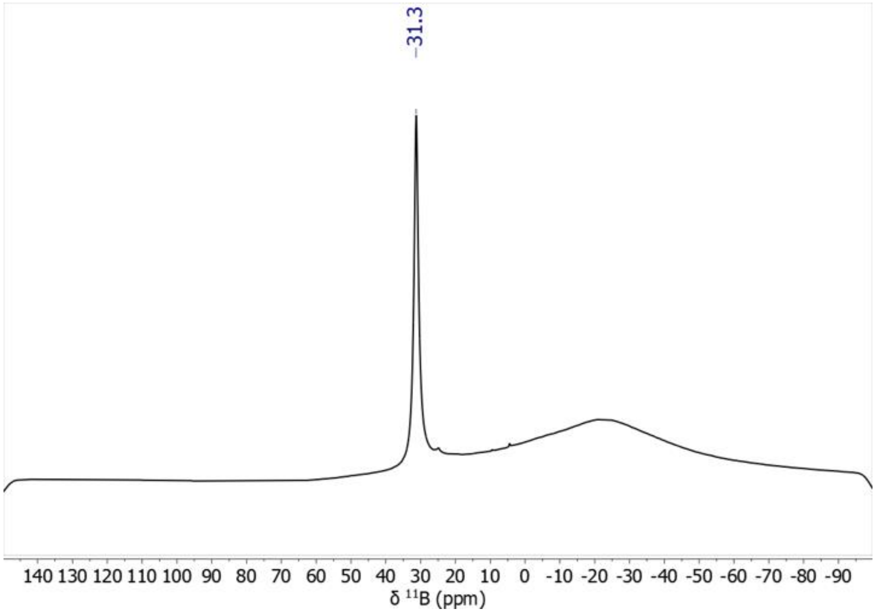

Figure S23. $^{11}B\{^1H\}$ NMR spectrum of a mixture of [K(crypt)$^+$][**NpMI**$^{•-}$] and B_2pin_2, following irradiation at 455 nm for 16h. The labelled resonance corresponds to B_2pin_2.

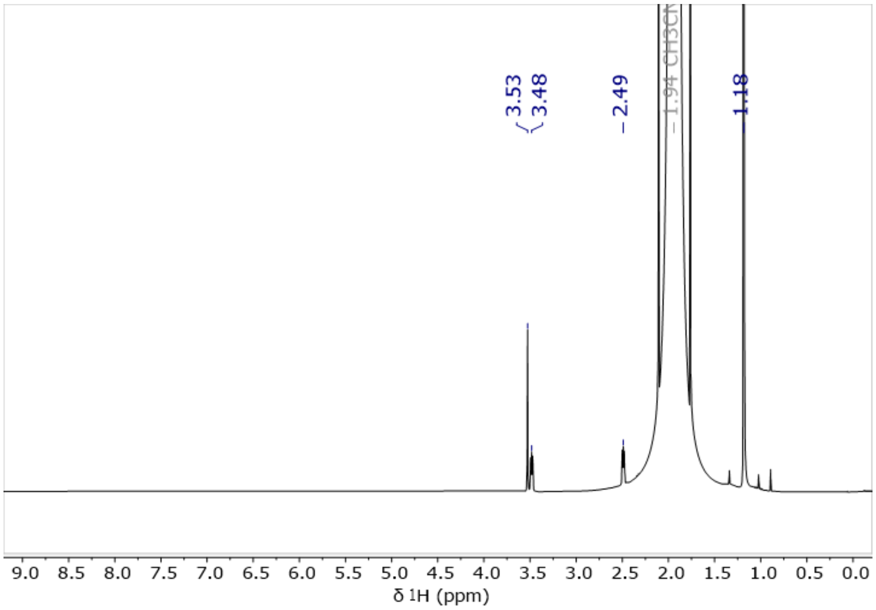

Figure S24. ^{1}H NMR spectrum of a mixture of [K(crypt)$^{+}$][**NpMI**$^{\bullet-}$] and B_2pin_2, following irradiation at 530 nm for 16h. The labelled resonances correspond to B_2pin_2 (1.18 ppm) and [K(crypt)$^{+}$].

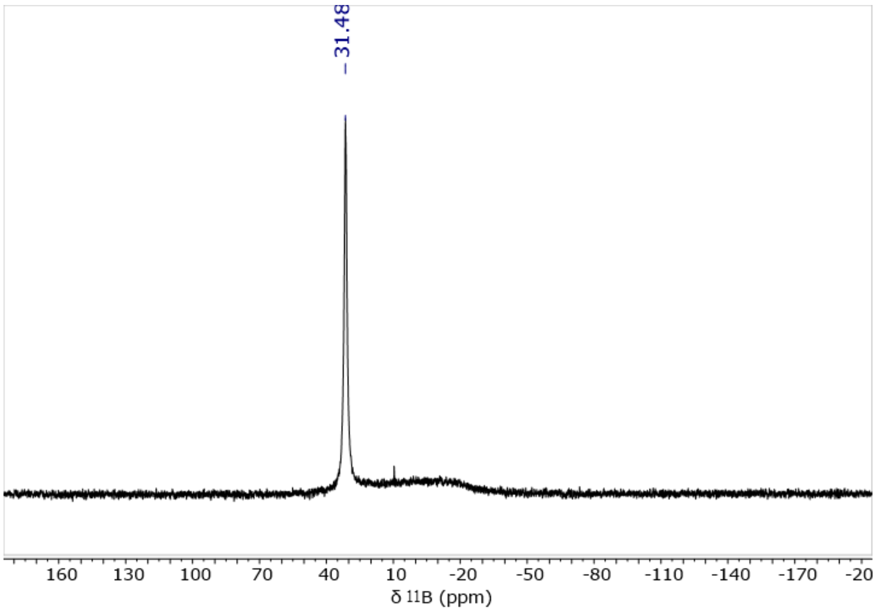

Figure S25. ^{11}B NMR spectrum of a mixture of [K(crypt)$^{+}$][**NpMI**$^{\bullet-}$] and B_2pin_2, following irradiation at 530 nm for 16h. The labelled resonance corresponds to B_2pin_2.

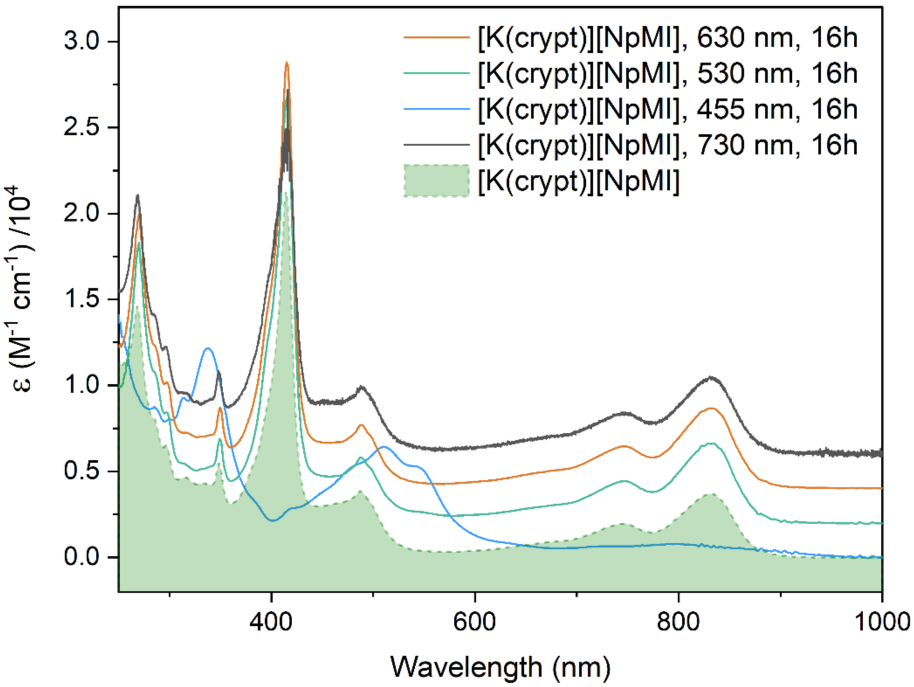

Figure S26. UV-vis absorption spectra of [K(crypt)+][**NpMI•−**], following irradiation by light of various wavelengths for 16 h. The *ε* data is offset vertically by 2 x10^3 (530 nm), 4 x10^3 (630 nm) or 6 x10^3 M^{-1} cm^{-1} (730 nm) for clarity. Irradiation was performed at 5 mM concentration in MeCN, then diluted to 1 mM concentration for recording in a 1 mm path length cuvette. The spectrum of [K(crypt)+][**NpMI•−**] is included for comparison.

Unlike all of the other cases investigated, where the lack of spectroscopic changes indicates [K(crypt)+][PC•−] to be photostable, UV-vis and ^{1}H NMR analysis both indicate that [K(crypt)+][**NpMI•−**] undergoes photodecomposition under 455 nm LED irradiation. The change in the UV-vis spectrum is particularly obvious, showing a completely different spectroscopic signature. As an aside, we note a qualitative similarity to the UV-vis spectrum assigned to [**NpMI**•H−] by Nocera *et al.*).[5] However, no resonances attributable to [**NpMI**•H−] were observed by ^{1}H NMR spectroscopy (in particular, the characteristic resonances around 4.1-4.2 ppm; Figure S19), suggesting that this is not in fact the origin of these absorbances. Instead, we speculate that they may be due to other, electronically similar (but currently unidentified) species such as other Meisenheimer-type structures, [**NpMI**•X−].

This observed photodecomposition raises the possibility that, at 455 nm specifically, the observed photoreactivity of [K(crypt)+][**NpMI•−**] towards Ar-Cl may in fact be mediated by its photodegradation products, rather than direct electron transfer from ***NpMI•−** to Ar-Cl.

At present this possibility cannot be rigorously excluded. However, we note that irradiation of [K(crypt)$^+$][**NpMI**$^{•-}$] and Ar-Cl, both in the presence and absence of a radical trap, yields qualitatively identical results at 455 nm as at 530 nm, including regeneration of neutral **NpMI** (see Figures S51 and S52 in section 3.5.2, below, and *c.f.* Figure 3b-d). This would seem to suggest a commonality of mechanism, and hence that **NpMI**$^{•-}$ photodecomposition – which is not observed at 530 nm – need not be mechanistically relevant (see also section 4.1, below).

3.2. UV-vis spectroscopy of [K(crypt)$^+$][PC$^{•-}$] in the presence of substrate

To determine whether the absorbance spectra of the photocatalyst radical anions changed in the presence of substrate (as might be expected with precomplexation or with quenching of the radical anion to the ground state), the absorbance spectra of [K(crypt)$^+$][PC$^{•-}$] were recorded in the presence of each of the three aryl chloride substrates used in this study, as shown in Figures S27 and S28. The concentration of the aryl chlorides was identical to that later used for reactivity studies (50 mM). No significant differences in the absorption spectra were observed when compared with [K(crypt)$^+$][PC$^{•-}$] alone.

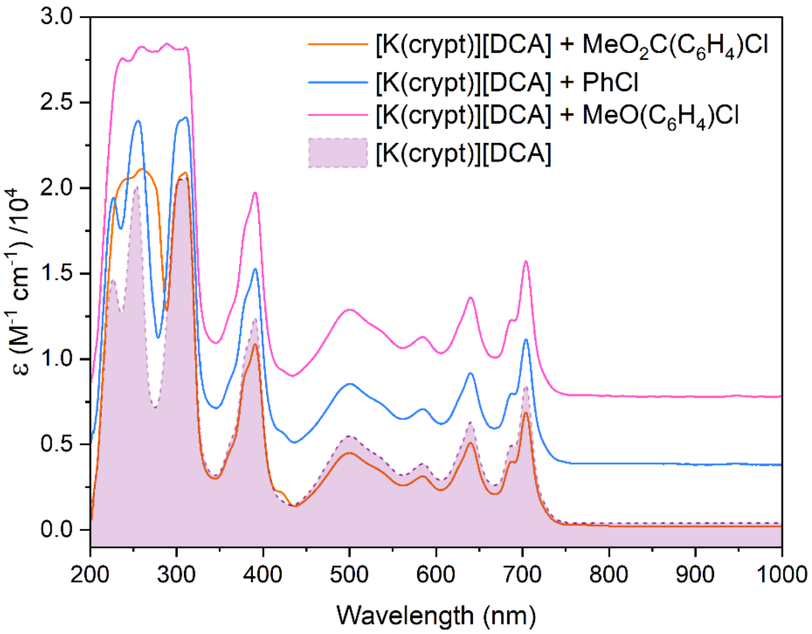

Figure S27. UV-visible absorption spectra of mixtures of [K(crypt)$^+$][**DCA**$^{•-}$] (1 mM) with ArCl substrates (50 mM), shown in comparison to the absorption spectrum of [K(crypt)$^+$][**DCA**$^{•-}$], all collected using a 1 mm path length cuvette. For clarity, spectra are offset by 4x10^3 M^{-1} cm^{-1} (PhCl) or 8x10^3 M^{-1} cm^{-1} (MeO(C_6H_4)Cl).

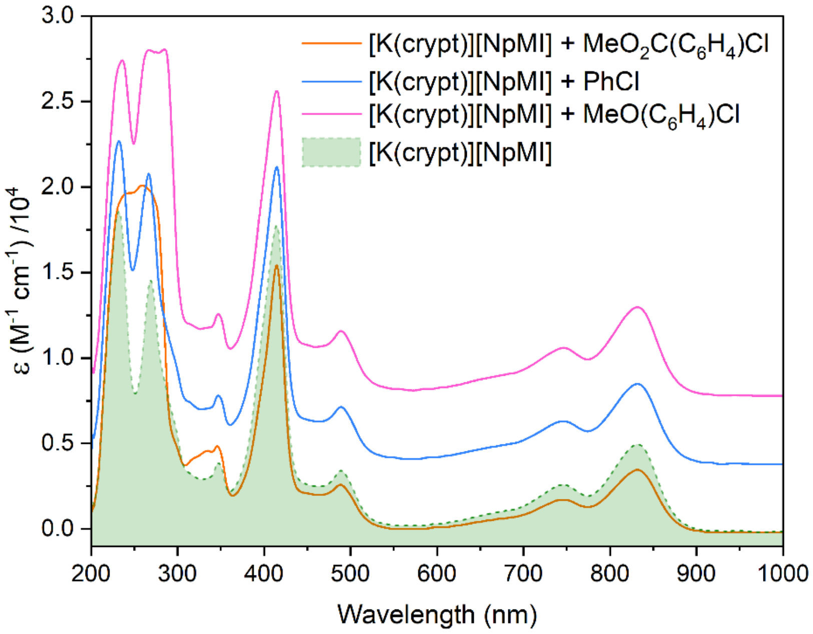

Figure S28. UV-visible absorption spectra of mixtures of [K(crypt)$^+$][**NpMI$^{\bullet-}$**] (1 mM) with ArCl substrates (50 mM), shown in comparison to the absorption spectrum of [K(crypt)$^+$][**NpMI$^{\bullet-}$**], all collected using a 1 mm path length cuvette. For clarity, spectra are offset by 4x10^3 M^{-1} cm^{-1} (PhCl) or 8x10^3 M^{-1} cm^{-1} (MeO(C_6H_4)Cl).

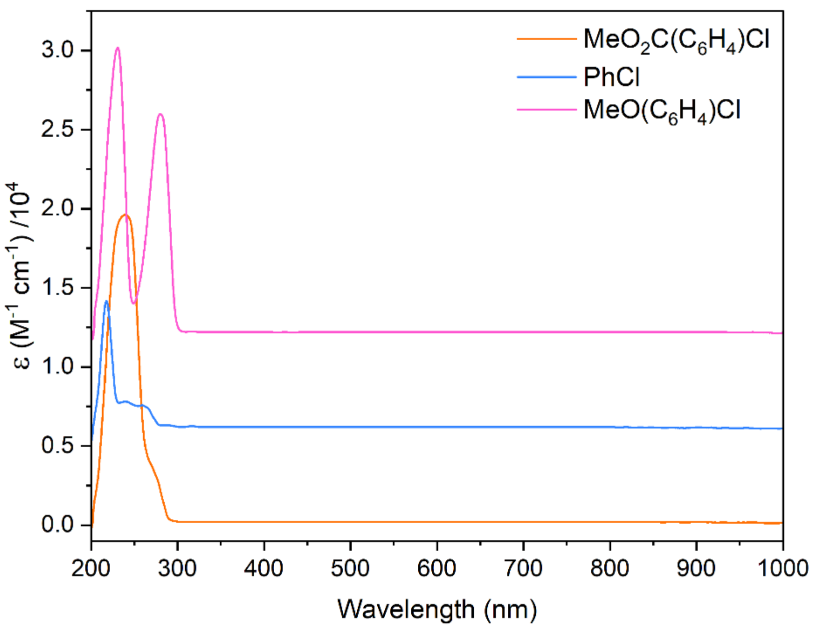

Figure S29. UV-visible absorption spectra of ArCl substrates used in this study, recorded at 10 mM concentration in a 1 mm path length cuvette. For clarity, the spectra are offset by 6x10^3 M^{-1} cm^{-1} (PhCl) or 1.2x10^4 M^{-1} cm^{-1} (MeO(C_6H_4)Cl).

3.3. Reactivity of [K(crypt)+][PC•−] in the dark

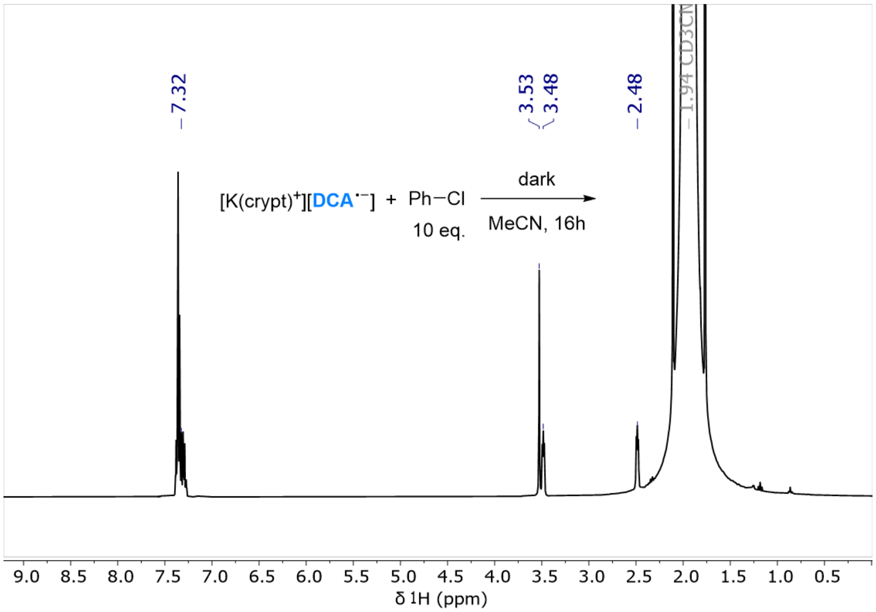

Figure S30. ^{1}H NMR spectrum of the reaction of [K(crypt)$^{+}$][**DCA$^{•-}$**] with PhCl, kept in the dark for 16h. Assignments: [K(crypt)$^{+}$], δ = 3.53, 3.48, 2.48 ppm; PhCl, δ = 7.25-7.41 (m) ppm.

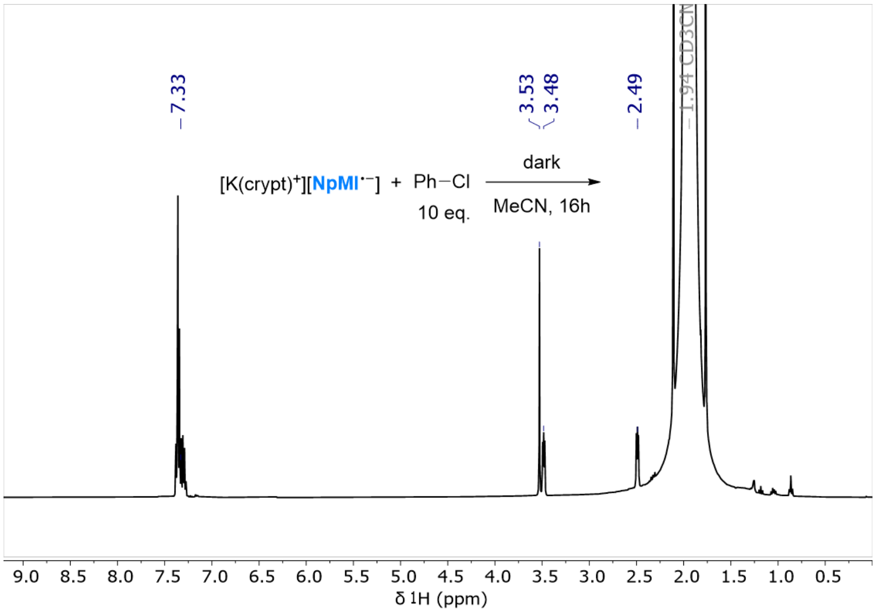

Figure S31. ^{1}H NMR spectrum of the reaction of [K(crypt)$^{+}$][**NpMI$^{•-}$**] with PhCl, kept in the dark for 16h. Assignments: [K(crypt)$^{+}$], δ = 3.53, 3.48, 2.49 ppm; PhCl, δ = 7.25-7.41 (m) ppm.

3.4. Reactivity of [K(crypt)$^+$][PC$^{\bullet-}$] under LED irradiation

A representative sample of ^{1}H and ^{31}P{^{1}H} NMR spectra are provided along with their assignments in this section. The product concentration in B_2pin_2-trapped reactions was too low to obtain useful ^{11}B NMR spectra, as product resonances were obscured by remaining B_2pin_2 and the borosilicate background.

Product assignments are collated in the table below, and were validated by spiking with authentic, purchased material ($PhPO(OMe)_2$, $MeO_2C(C_6H_4)Bpin$) or material prepared by independent synthesis ($MeO(C_6H_4)PO(OMe)_2$).[6] More detailed assignments are given for each spectrum.

Collected ^{1}H and ^{31}P NMR resonances assigned to the products of the reduction and radical trapping reactions are detailed in Table S1.

Table S1. Selected ^{1}H and ^{31}P NMR resonances assigned to radical trapping reaction products. Aromatic ^{1}H signals are not listed due to typically overlapping with other resonances, and hence not being used for analysis.

Product	δ ^{1}H (/ppm)	δ ^{31}P (/ppm)
$PhPO(OMe)_2$	3.66 (–$PO(OMe)_2$, 6H)	21.4
$MeO_2C(C_6H_4)PO(OMe)_2$	3.88 (–CO_2Me, 3H), 3.69 (–$PO(OMe)_2$, 6H)	19.6
$MeO(C_6H_4)PO(OMe)_2$	3.84 (–OMe, 3H), 3.63 (–$PO(OMe)_2$, 6H)	22.2
$PhPO(OEt)_2$	4.02 (–CH_2, 4H), 1,24 (–CH_3, 6H)	18.5
$MeO_2C(C_6H_4)Bpin$	3.85 (–CO_2Me, 3H), 1.31 (–Bpin, 12H)	*n/a*

To confirm that the >100% conversions reported in Tables 1 and 2 of the main manuscript when using $P(OMe)_3$ as a radical trap were reproducible, these experiments were generally carried out in triplicate. The individual conversions measured were:

187%, 170%, 164% (Table 1, entry 13)
238%, 257%, 247% (Table 1, entry 17)
158%, 129%, 136% (Table 1, entry 21)
178%, 143%, 150% (Table 2, entry 1)
235%, 236%, 203% (Table 2, entry 5)
217%, 180%, 188% (Table 2, entry 9)

3.4.1. GC-MS analysis of reactions of [K(crypt)+][PC•−] with PhCl

To determine the products of the reactions between [K(crypt)+][PC•−] and PhCl in reactions without radical traps, the product mixtures were subjected to tandem GC-MS analysis. Following irradiation of [K(crypt)+][PC•−] and PhCl in the standard concentrations for 16 h, a portion of the reaction mixture was diluted by a factor of 100 in HPLC-grade acetonitrile and submitted for a single-ion GC-MS measurement experiment. The GC was run on a 0.25 mm diameter x 30 m, silica 0.25 µm film column with a ramp in temperature from 35 °C to 250 °C over the course of 8 minutes. The MS was run in negative mode with an electron ionization method. Peaks corresponding to the supposed products of the reduction of PhCl, benzene (the product of H-atom abstraction by a phenyl radical) and biphenyl (the product of homocoupling of phenyl radicals), were identified. The peaks were integrated and compared against calibration curves for benzene and biphenyl (Figure S32). The concentrations of biphenyl were significantly lower than the lowest concentrations used for the calibration curve, and close to the signal-to-noise detection limit. We note some overlap between benzene (m/z = 78, 52, 51) and propanenitrile present in the solvent (m/z = 52, 51), which was eliminated by integrating m/z = 78 only. The calculated conversions of benzene and biphenyl are compiled in Table S2.

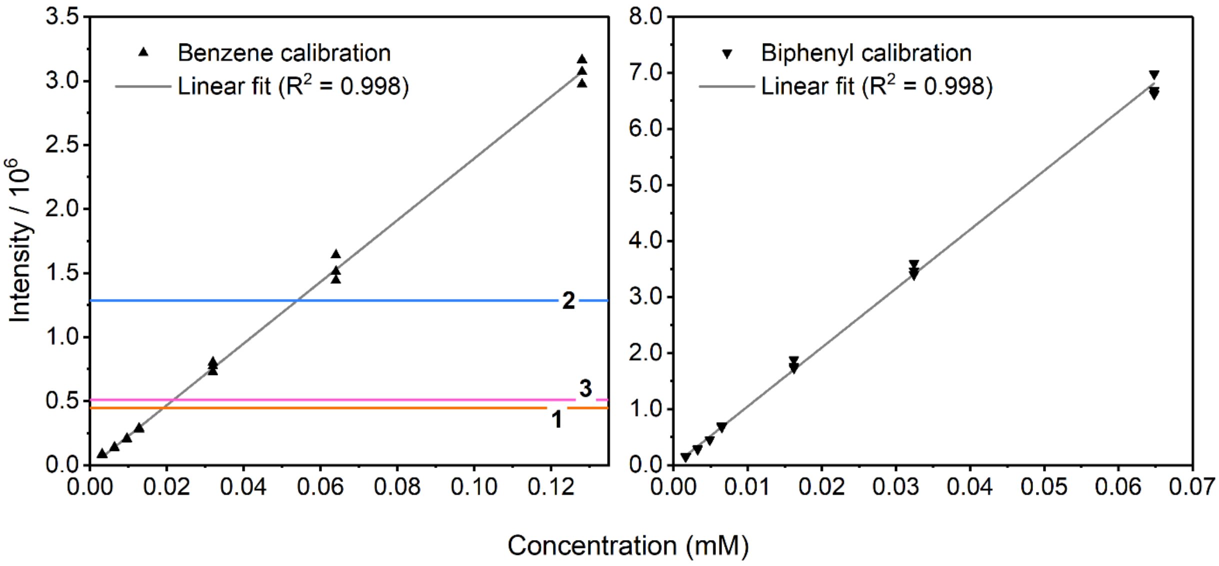

Figure S32. GC calibration curves as determined for benzene and biphenyl. Linear fits: Benzene, $2.409\times10^7 c - 1.370\times10^5$; Biphenyl, $1.052\times10^8 c - 4.199\times10^3$. Calculated benzene concentrations for the three reactions are shown as horizontal lines labelled **1** ([K(crypt)+][**DCA**•−], 455 nm), **2** ([K(crypt)+][**NpMI**•−], 455 nm), and **3** ([K(crypt)+][**NpMI**•−], 530 nm).

Table S2. Calculated concentrations and conversions of chlorobenzene to benzene and biphenyl by [K(crypt)$^+$][PC$^{\bullet-}$] as determined by GC.

**[K(crypt)$^+$][PC$^{\bullet-}$]**	**λ /nm**	**Benzene conc. /μM[a]**	**Conv. To benzene/%**	**Biphenyl conc. /μM[b]**	**Conv. to biphenyl /%**
[K(crypt)$^+$][**DCA$^{\bullet-}$**]	455	19	38	0.10	0.2
[K(crypt)$^+$][**NpMI$^{\bullet-}$**]	455	54	108[c]	0.099	0.2
[K(crypt)$^+$][**NpMI$^{\bullet-}$**]	530	22	44	0.058	0.1

[a]Limit of linear detection for benzene is 6.4 μM. [b]Detected biphenyl concentrations were lower than the lowest concentration biphenyl standard used (1.6 μM). [c]Error in conversion is expected to be greater than 10% due to multiple dilutions of small volumes.

3.4.2. Reactivity of [K(crypt)$^+$][DCA$^{\bullet-}$] under LED irradiation

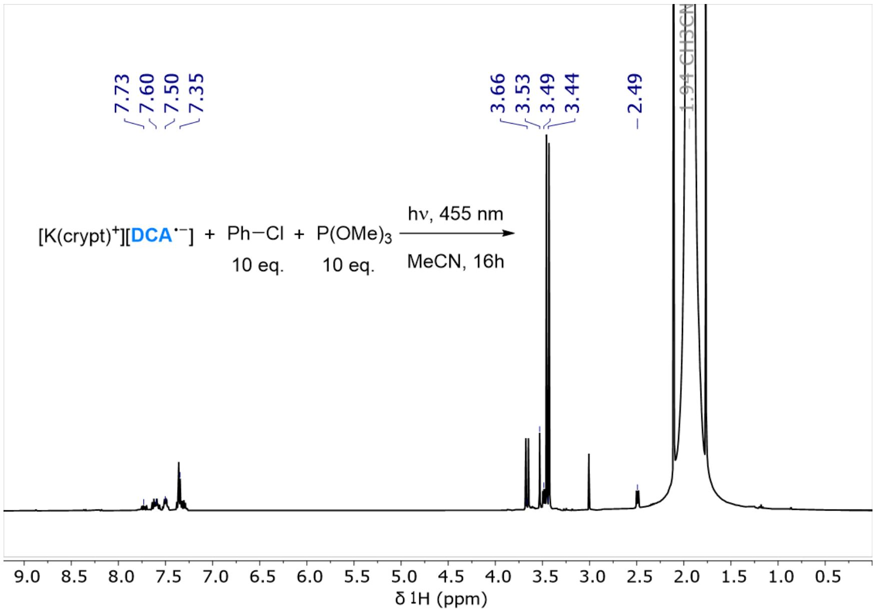

Figure S33. ^{1}H NMR spectrum of the reaction of [K(crypt)$^+$][**DCA$^{\bullet-}$**] with PhCl in the presence of $P(OMe)_3$, under 455 nm irradiation for 16 h. Assignments: [K(crypt)$^+$], δ = 3.53, 3.49, 2.49 ppm; PhCl, δ = 7.25-7.41 ppm; $P(OMe)_3$, δ = 3.44 ppm; Ph_3PO, δ = 7.60, 7.50 ppm; $PhPO(OMe)_2$, δ = 3.66 ppm. Addition of authentic $PhPO(OMe)_2$ was used to validate the product assignment.

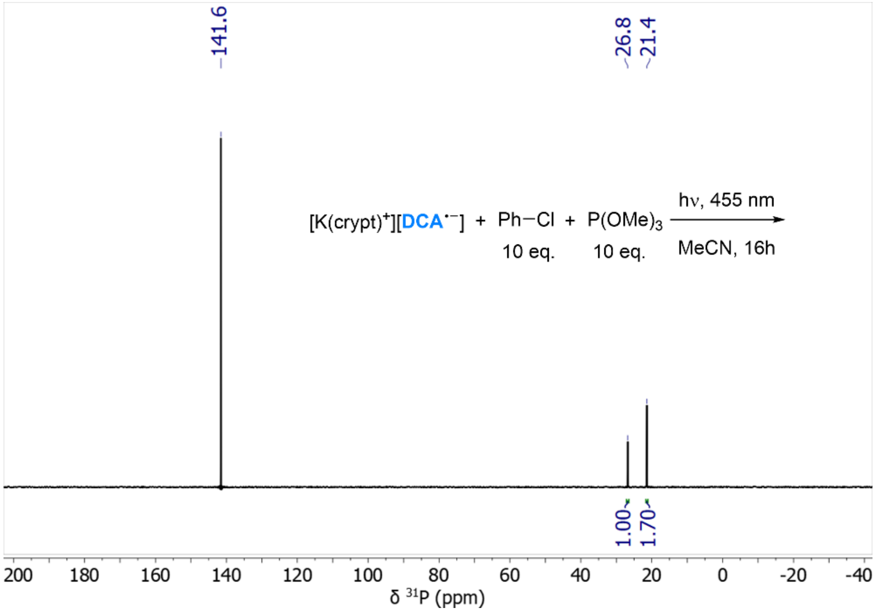

Figure S34. ${}^{31}P\{{}^{1}H\}$ NMR spectrum of the reaction of K(crypt)+][**DCA•−**] with PhCl in the presence of $P(OMe)_3$, under 455 nm irradiation for 16 h. Assignments: $P(OMe)_3$, δ = 141.6 ppm; Ph_3PO, δ = 26.8 ppm; $PhPO(OMe)_2$, δ = 21.4 ppm. Addition of authentic $PhPO(OMe)_2$ was used to validate the product assignment.

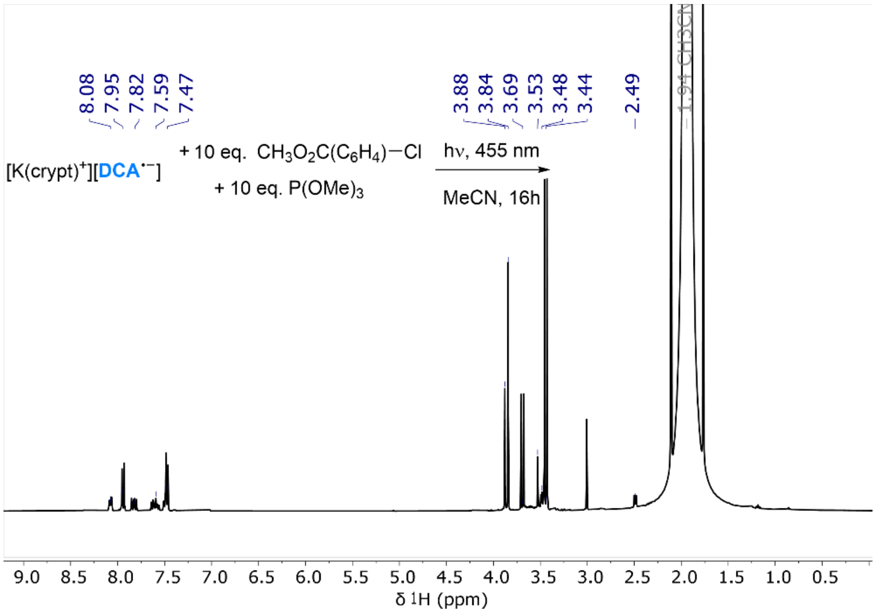

Figure S35. ${}^{1}H$ NMR spectrum of the reaction of [K(crypt)+][**DCA•−**] with $MeO_2C(C_6H_4)Cl$ in the presence of $P(OMe)_3$, under 455 nm irradiation for 16 h. Assignments: [K(crypt)+], δ = 3.53, 3.48, 2.49 ppm; $MeO_2C(C_6H_4)Cl$, δ = 7.95, 7.47, 3.84 ppm; $P(OMe)_3$, δ = 3.44 ppm; Ph_3PO, δ = 7.59 ppm; $MeO_2C(C_6H_4)PO(OMe)_2$, δ = 8.08, 7.82, 3.88, 3.69 ppm.

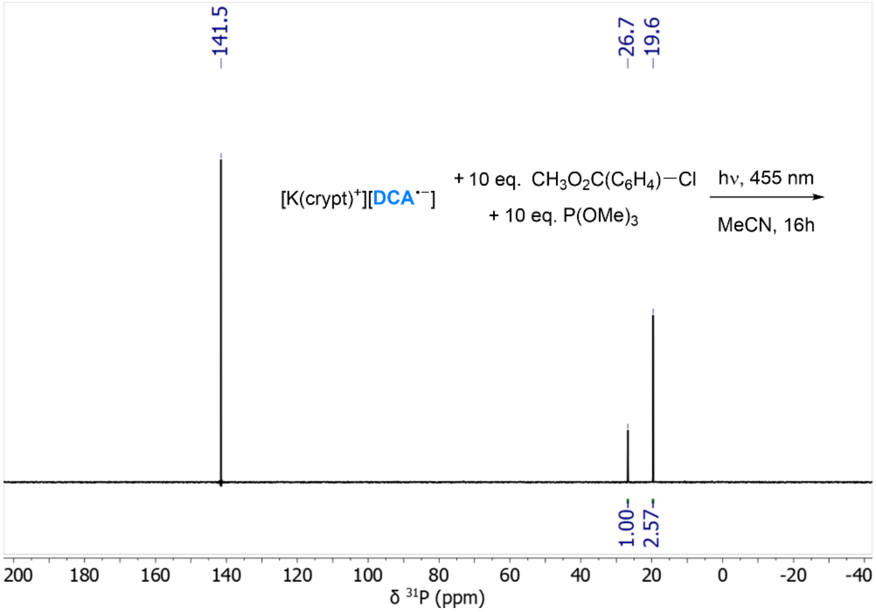

Figure S36. $^{31}P\{^{1}H\}$ NMR spectrum of the reaction of $[K(crypt)^+][\mathbf{DCA}^{\bullet-}]$ with $MeO_2C(C_6H_4)Cl$ in the presence of $P(OMe)_3$, under 455 nm irradiation for 16 h. Assignments: $P(OMe)_3$, δ = 141.5 ppm; Ph_3PO, δ = 26.7 ppm; $MeO_2C(C_6H_4)PO(OMe)_2$, δ = 19.6 ppm.

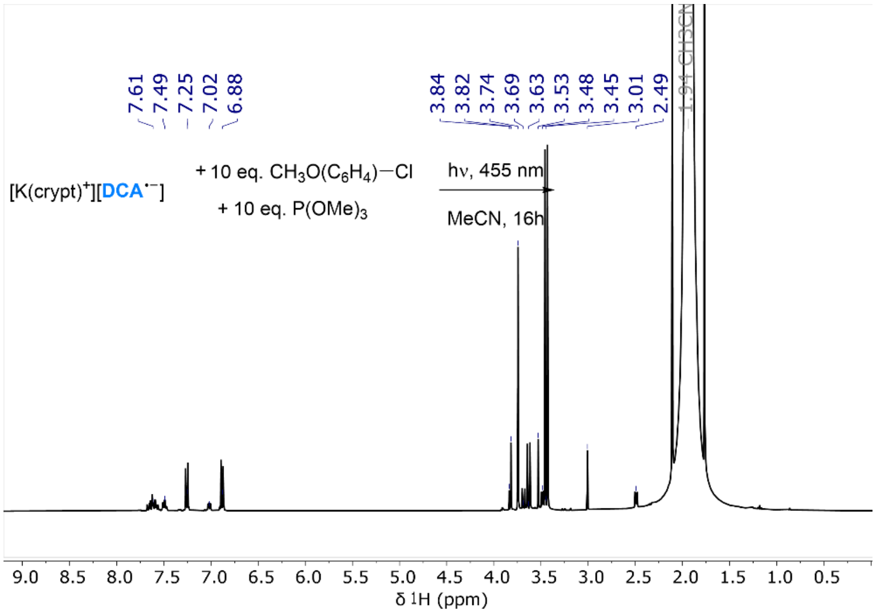

Figure S37. ^{1}H NMR spectrum of the reaction of $[K(crypt)^+][\mathbf{DCA}^{\bullet-}]$ with $MeO(C_6H_4)Cl$ in the presence of $P(OMe)_3$, under 455 nm irradiation for 16 h. Assignments: $[K(crypt)^+]$, δ = 3.53, 3.48, 2.49 ppm; $MeO(C_6H_4)Cl$, δ = 7.25, 6.88, 3.74 ppm; $P(OMe)_3$, δ = 3.45 ppm; Ph_3PO, δ = 7.61, 7.49 ppm; $MeO(C_6H_4)PO(OMe)_2$, δ = 7.02, 3.82, 3.63 ppm; 2nd product, δ = 3.84, 3.69 ppm. Addition of authentic $MeO(C_6H_4)PO(OMe)_2$ was used to validate the product assignment.

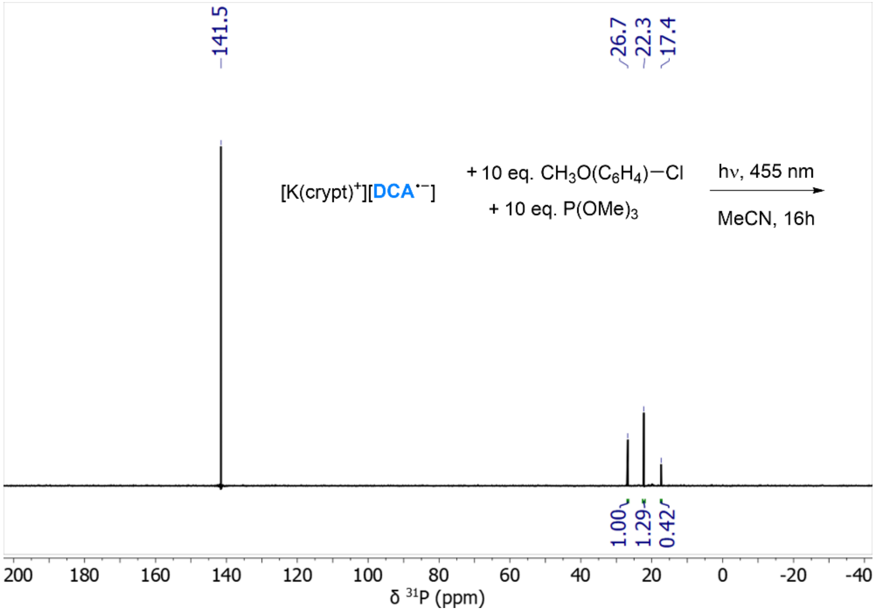

Figure S38. ${}^{31}P\{{}^{1}H\}$ NMR spectrum of the reaction of [K(crypt)$^+$][**DCA$^{\bullet-}$**] with MeO(C_6H_4)Cl in the presence of $P(OMe)_3$, under 455 nm irradiation for 16 h. Assignments: $P(OMe)_3$, δ = 141.5 ppm; Ph_3PO, δ = 26.7 ppm; MeO(C_6H_4)PO(OMe)$_2$, δ = 22.3 ppm; 2nd unidentified product, δ = 17.4 ppm. Addition of authentic MeO(C_6H_4)PO(OMe)$_2$ was used to validate the product assignment.

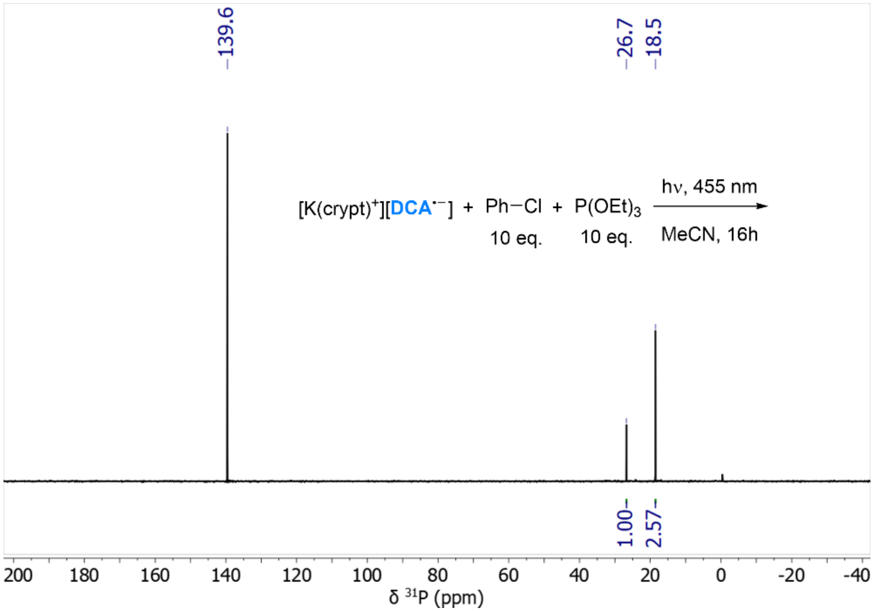

Figure S39. ${}^{31}P\{{}^{1}H\}$ NMR spectrum of the products of the reaction of [K(crypt)$^+$][**DCA$^{\bullet-}$**] with PhCl in the presence of $P(OEt)_3$, under 455 nm irradiation for 16h. Assignments: $P(OEt)_3$, δ = 139.6 ppm; Ph_3PO, δ = 26.7 ppm; PhPO(OEt)$_2$, δ = 18.5 ppm.

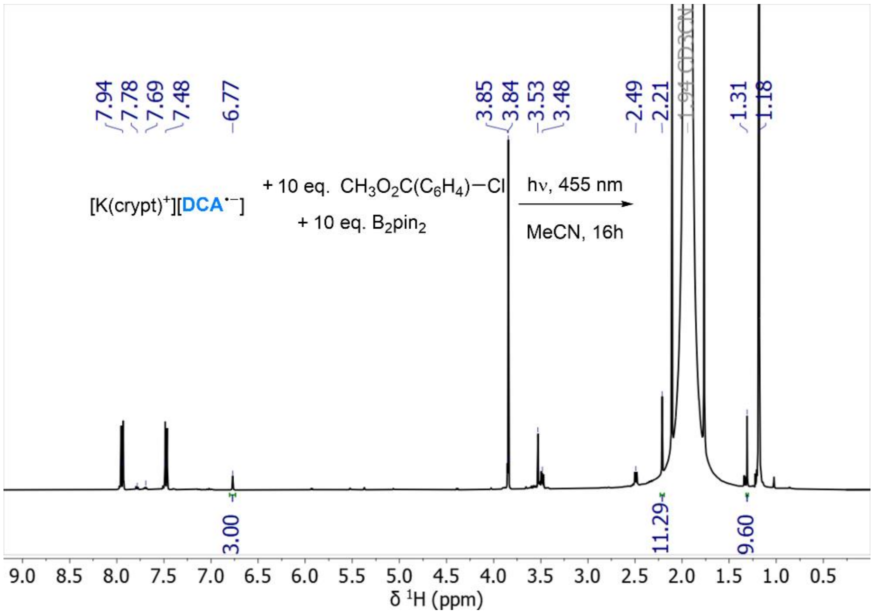

Figure S40. ^{1}H NMR spectrum of the products of the reaction of $[K(crypt)^+]$[**DCA**$^{\bullet-}$] with $MeO_2C(C_6H_4)Cl$ in the presence of B_2pin_2, under 455 nm irradiation for 16h. Assignments: $[K(crypt)^+]$, δ = 3.53, 3.48, 2.49 ppm; $MeO_2C(C_6H_4)Cl$, δ = 7.94, 7.48, 3.84 ppm; B_2pin_2, δ = 1.18 ppm; Mesitylene, δ = 6.77, 2.21 ppm; $MeO_2C(C_6H_4)Bpin$, δ = 3.85, 1.31 (–Bpin, 12H) ppm. Addition of authentic $MeO_2C(C_6H_4)Bpin$ was used to validate the product assignment.

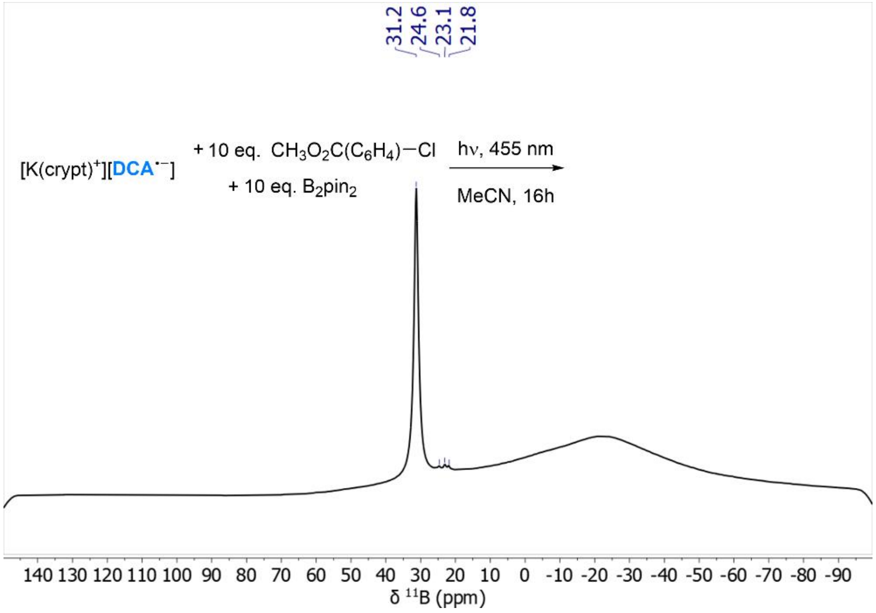

Figure S41. $^{11}B\{^{1}H\}$ NMR spectrum of the products of the reaction of $[K(crypt)^+]$[**DCA**$^{\bullet-}$] with $MeO_2C(C_6H_4)Cl$ in the presence of B_2pin_2, under 455 nm irradiation for 16h. Addition of authentic $MeO_2C(C_6H_4)Bpin$ was used to validate the product assignment. However, the product resonance overlaps with the B_2pin_2 starting material, limiting the usefulness of ^{11}B NMR as a diagnostic tool.

3.4.3. Reactivity of [K(crypt)+][NpMI•−] under LED irradiation

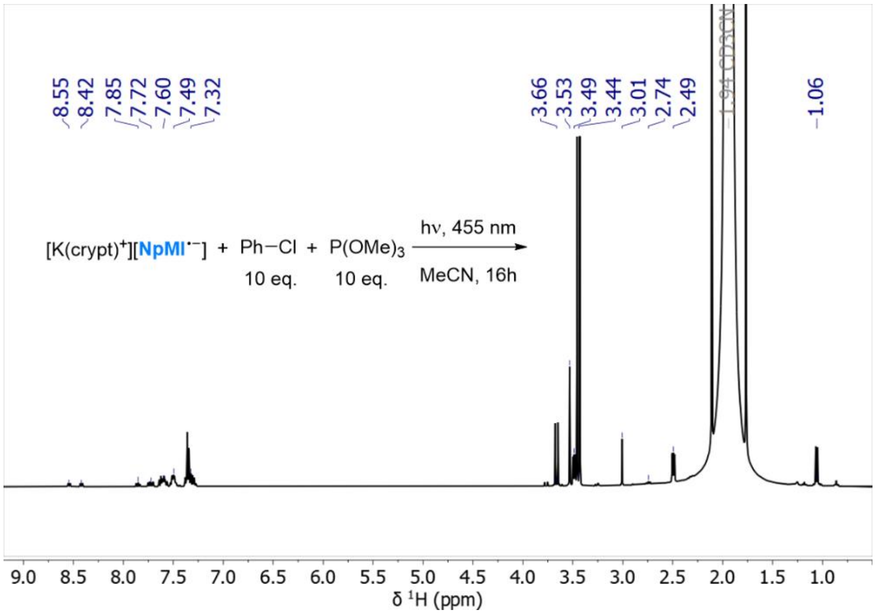

Figure S42. ^{1}H NMR spectrum of the products of the reaction of [K(crypt)+][**NpMI•−**] with PhCl in the presence of $P(OMe)_3$, under 455 nm irradiation for 16h. Assignments: **NpMI**, δ = 8.55, 8.42, 7.85, 2.74, 1.06 ppm; [K(crypt)+], δ = 3.53, 3.49, 2.49 ppm; PhCl, δ = 7.32 (m) ppm; $P(OMe)_3$, δ = 3.44 ppm; Ph_3PO, δ = 7.60, 7.49 ppm; $PhPO(OMe)_2$, δ = 3.66 ppm.

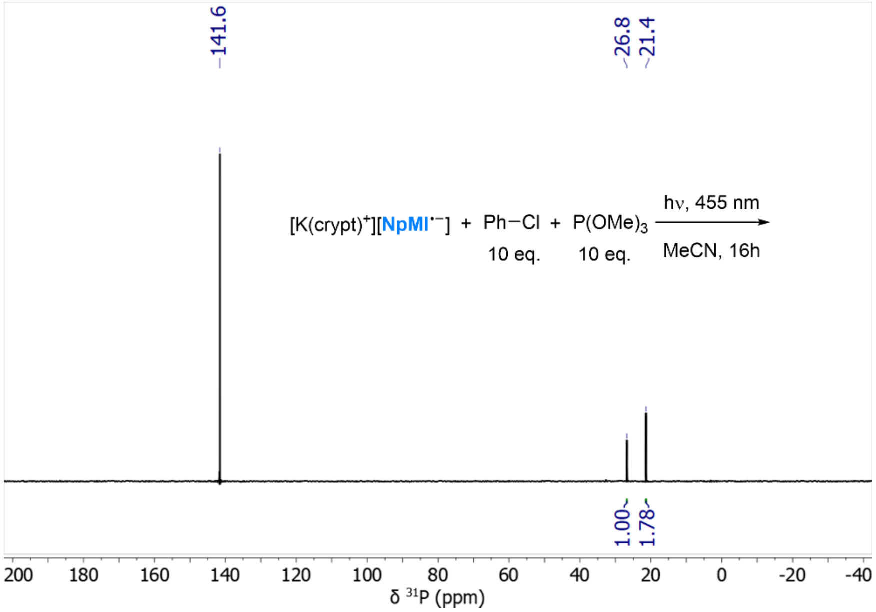

Figure S43. $^{31}P\{^{1}H\}$ NMR spectrum of the products of the reaction of [K(crypt)+][**NpMI•−**] with PhCl in the presence of $P(OMe)_3$, under 455 nm irradiation for 16h. Assignments: $P(OMe)_3$, δ = 141.6 ppm; Ph_3PO, δ = 26.8 ppm; $PhPO(OMe)_2$, δ = 21.4 ppm.

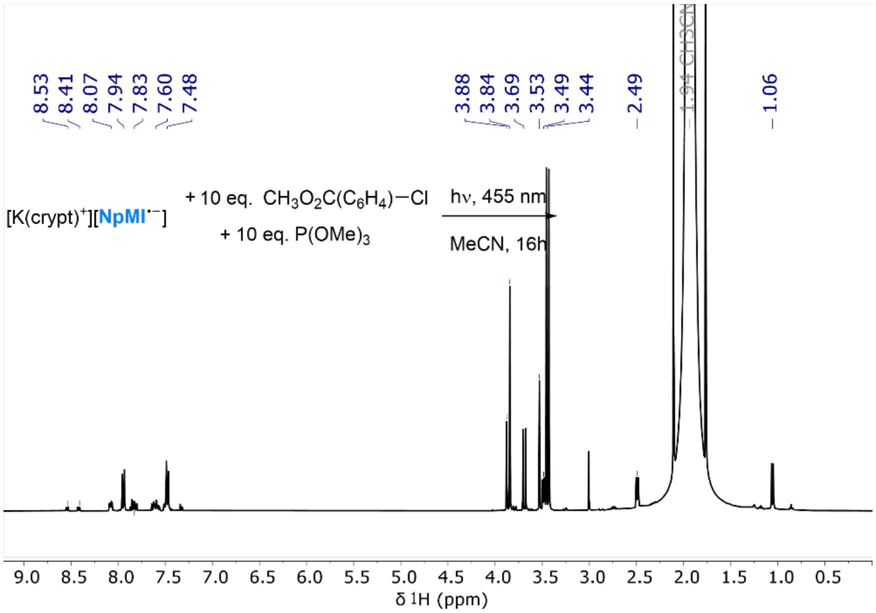

Figure S44. ^{1}H NMR spectrum of the products of the reaction of [K(crypt)$^+$][**NpMI•−**] with $MeO_2C(C_6H_4)Cl$ in the presence of $P(OMe)_3$, under 455 nm irradiation for 16h. Assignments: **NpMI**, δ = 8.53, 8.41, 7.83, 1.06 ppm; [K(crypt)$^+$], δ = 3.53, 3.49, 2.49 ppm; $MeO_2C(C_6H_4)Cl$, δ = 7.94, 7.48, 3.84 ppm; $P(OMe)_3$, δ = 3.44 ppm; Ph_3PO, δ = 7.60 ppm; $MeO_2C(C_6H_4)PO(OMe)_2$, δ = 3.88, 3.66 ppm.

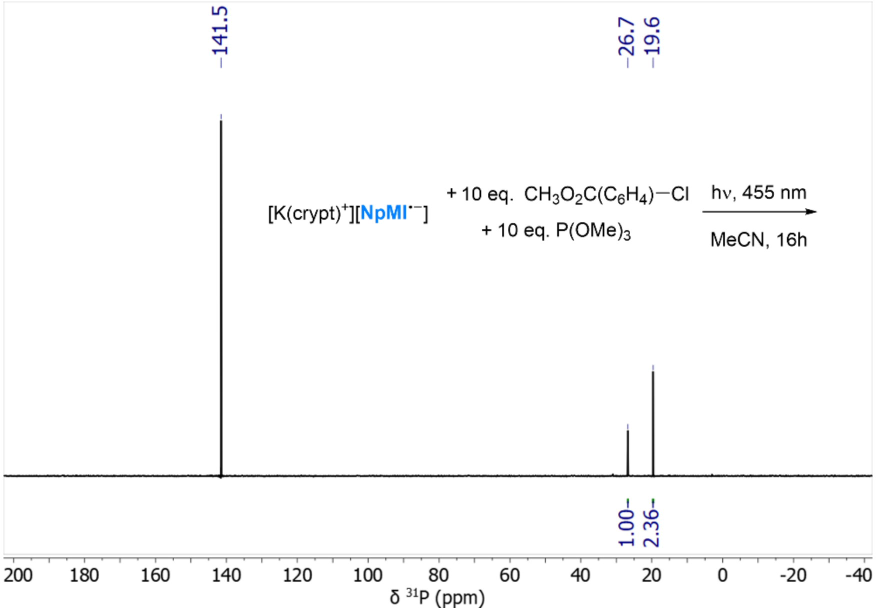

Figure S45. $^{31}P\{^1H\}$ NMR spectrum of the products of the reaction of [K(crypt)$^+$][**NpMI•−**] with $MeO_2C(C_6H_4)Cl$ in the presence of $P(OMe)_3$, under 455 nm irradiation for 16h. Assignments: $P(OMe)_3$, δ = 141.5 ppm; Ph_3PO, δ = 26.7 ppm; $MeO_2C(C_6H_4)PO(OMe)_2$, δ = 19.6 ppm.

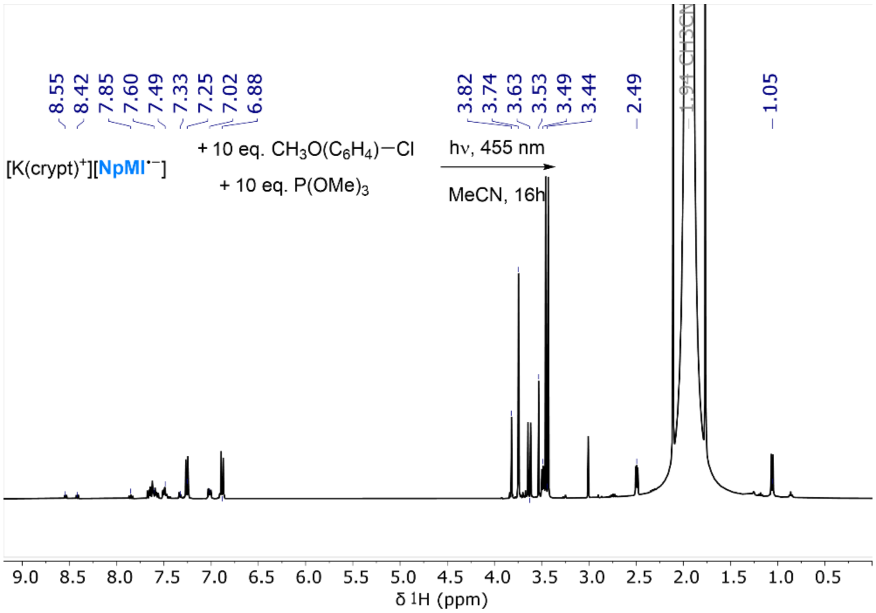

Figure S46. ^{1}H NMR spectrum of the products of the reaction of [K(crypt)$^+$][**NpMI**$^{•-}$] with MeO(C_6H_4)Cl in the presence of P(OMe)$_3$, under 455 nm irradiation for 16h. Assignments: **NpMI**, δ = 8.55, 8.42, 7.85, 1.05 ppm; [K(crypt)$^+$], δ = 3.53, 3.49, 2.49 ppm; MeO(C_6H_4)Cl, δ = 7.25, 6.88, 3.74 ppm; P(OMe)$_3$, δ = 3.44 ppm; Ph_3PO, δ = 7.60, 7.49 ppm; MeO(C_6H_4)PO(OMe)$_2$, δ = 7.02, 3.88, 3.66 ppm.

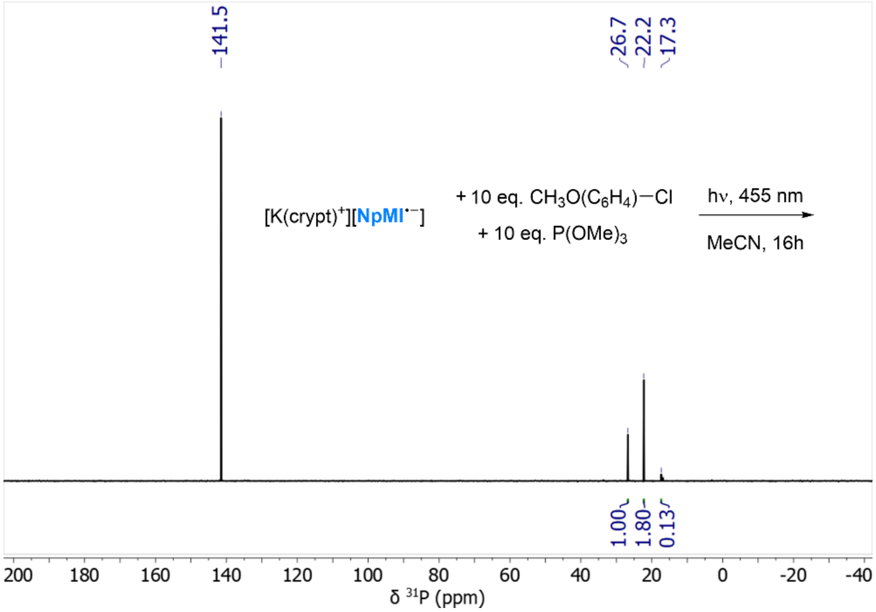

Figure S47. ^{1}H NMR spectrum of the products of the reaction of [K(crypt)$^+$][**NpMI**$^{•-}$] with MeO(C_6H_4)Cl in the presence of P(OMe)$_3$, under 455 nm irradiation for 16h. Assignments: P(OMe)$_3$, δ = 141.5 ppm; Ph_3PO, δ = 26.7 ppm; MeO(C_6H_4)PO(OMe)$_2$, δ = 22.2 ppm; 2nd unidentified product, δ = 17.3 ppm.

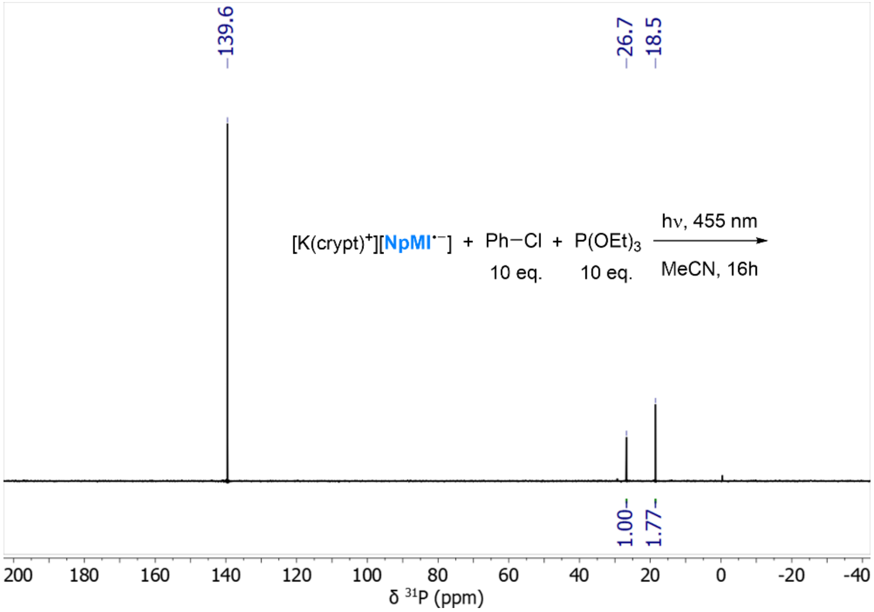

Figure S48. $^{31}P\{^{1}H\}$ NMR spectrum of the products of the reaction of [K(crypt)$^{+}$][**NpMI**$^{•–}$] with PhCl in the presence of $P(OEt)_3$, under 455 nm irradiation for 16h. Assignments: $P(OEt)_3$, δ = 139.6 ppm; Ph_3PO, δ = 26.7 ppm; $PhPO(OEt)_2$, δ = 18.5 ppm.

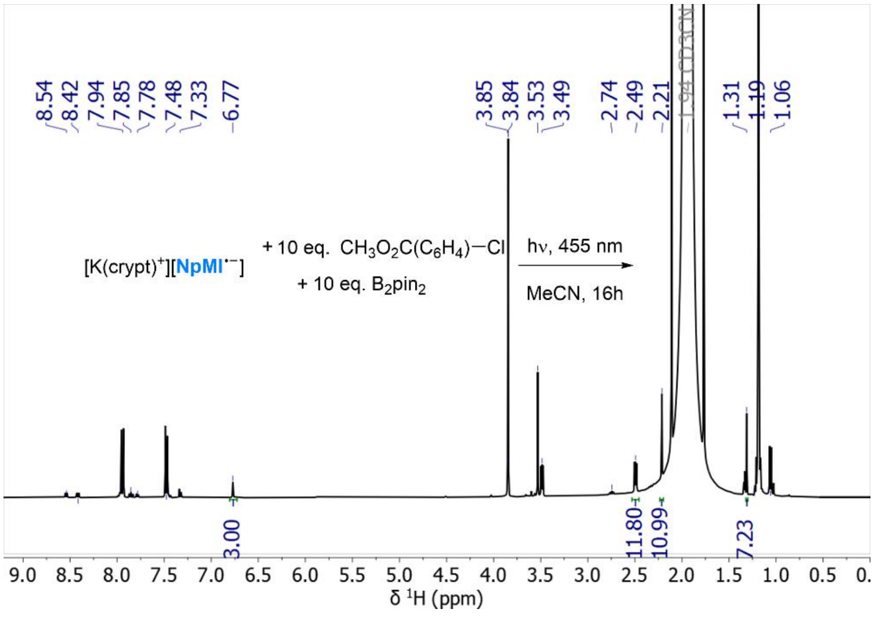

Figure S49. ^{1}H NMR spectrum of the products of the reaction of [K(crypt)$^{+}$][**NpMI**$^{•–}$] with $MeO_2C(C_6H_4)Cl$ in the presence of B_2pin_2, under 455 nm irradiation for 16h. Assignments: **NpMI**, δ = 8.54, 8.42, 7.85, 2.74, 1.06 ppm; [K(crypt)$^{+}$], δ = 3.53, 3.49, 2.49 ppm; $MeO_2C(C_6H_4)Cl$, δ = 7.94, 7.48, 3.84 ppm; B_2pin_2, δ = 1.19 ppm; Mesitylene, δ = 6.77, 2.21 ppm; $MeO_2C(C_6H_4)Bpin$, δ = 3.85, 1.31 (–Bpin, 12H) ppm.

3.4.4. Photoreactivity at lower [K(crypt)+][PC•−] concentration

To exclude the possibility that observed differences in photoreactivity at different wavelengths could be due to limited light penetration caused by the relatively high, wavelength-dependent optical density of the model reaction mixture (despite the use of narrow reaction vessels), several representative model reactions were repeated using a reduced concentration of [K(crypt)+][PC•−] (0.5 mM, i.e. 10% of 'standard' for this study), while keeping all other reaction parameters constant (including substrate concentration). The results of these reactions are shown in Table S3, below, and show the same pattern of wavelength-dependence as at higher concentrations (*c.f.* Tables 1, entries 1-3 and Table 2, entries 1-3).

Table S3. Photoreactivity of 0.5 mM [K(crypt)+][PC•−] with PhCl in presence of $P(OMe)_3$.

Photocatalyst	λ / nm	Conv. / %
[K(crypt)+][**DCA•−**]	455	920
[K(crypt)+][**DCA•−**]	530	0
[K(crypt)+][**DCA•−**]	630	0
[K(crypt)+][**NpMI•−**]	455	320
[K(crypt)+][**NpMI•−**]	530	310
[K(crypt)+][**NpMI•−**]	630	0

It is interesting to note the clearly higher turnover achieved (relative to [PC•−]) under these lower concentration conditions. One simple explanation for this observation could be that the desired Ph• radical-trapping pathway is better able to outcompete Ph• trapping by remaining PC•−, due to the reduced concentration of the latter (see section 4.2).

3.5 Quantification of remaining PC

To determine the fate of the PC moieties, ^1H NMR resonances assigned to neutral PC were integrated relative to an internal standard upon completion of several model reactions.

3.5.1 Quantification of remaining DCA^0

For [K(crypt)$^+$][**DCA**$^{\bullet-}$], the 16 h reaction between PhCl and $P(OMe)_3$ under 455 nm irradiation was used as a model reaction. Due to very low solubility of neutral **DCA** in MeCN, volatiles were removed from the final reaction mixture and $CDCl_3$ was added, along with 1 eq. of a mesitylene internal standard. ^1H NMR resonances consistent with **DCA** were detected; however, integration suggested only a low yield (25%) for its regeneration (Figure S50).

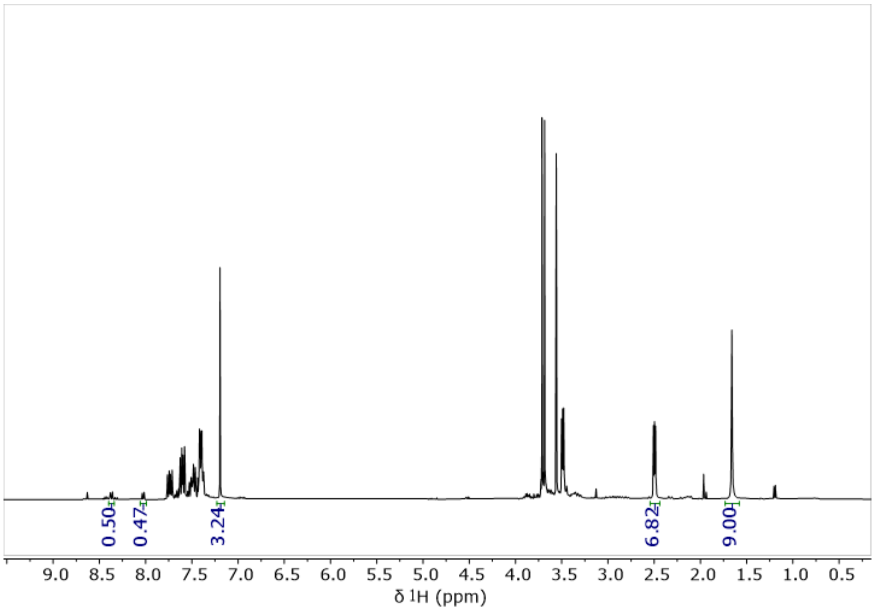

Figure S50. ^1H NMR spectrum of the products of the reaction of [K(crypt)$^+$][**DCA**$^{-\bullet}$] with PhCl and $POMe_3$, following 455 nm irradiation for 16 h. Resonances attributed to **DCA** and [K(crypt)$^+$] are integrated relative to 1 eq. mesitylene as an internal standard. Due to overlap of the PhCl and product resonances with the NMR solvent no accurate chemical shifts are given. The regenerated yield of DCA was 25%.

This is qualitatively consistent with observations made during irradiation of [K(crypt)$^+$][**DCA**$^{•−}$] in the presence of PhCl but the absence of a radical trap, where significant decomposition was observed (see Figure 3b). This decomposition is presumed to involve attack of initially formed Ph$^•$ on remaining **DCA**$^{•−}$, in line with established reactivity for related cyanoarenes (Scheme S1).[7] These results suggest that this pathway remains kinetically competitive, even in the presence of $P(OMe)_3$ (i.e. **DCA**$^{•−}$ and $P(OMe)_3$ are similarly effective traps for Ph$^•$).

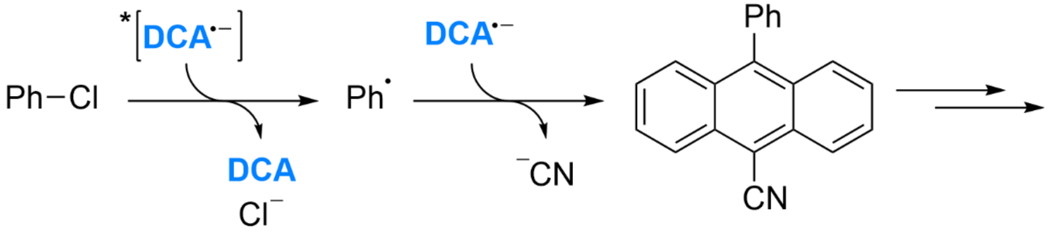

Scheme S1. Proposed, outline mechanism for degradation of the **DCA** backbone upon photoirradiation of [K(crypt)$^+$][**DCA**$^{•−}$] in the presence of PhCl.

It should be noted that based on their structures (e.g. 9-cyano-10-phenylanthracene) the **DCA** decomposition products indicated above could also be photochemically active, and it is therefore possible that their formation could have an impact on the overall reaction outcome. However, based on the above mechanistic rationale, their initial formation should itself still require reduction of PhCl *via* **DCA**$^{•−}$ photoexcitation (see section 4.2, below).

3.5.2 Quantification of remaining NpMI^0

For [K(crypt)$^+$][**NpMI$^{•-}$**] several model reactions were investigated, with examples at both 455 nm and 530 nm being chosen. As can be seen in Figures S51 and S52 below, in the reactions between $MeO_2C(C_6H_4)Cl$ and $P(OMe)_3$ at 455 nm and at 530 nm, reformation of neutral **NpMI** could be observed directly by ^1H NMR spectroscopy.

Quantification was achieved through integration relative to a subsequently added mesitylene internal standard and indicated significantly higher yields of recovery than for **DCA** (69% and *ca.* 100%, respectively). This is qualitatively consistent with observations made during irradiation of [K(crypt)$^+$][PC$^{•-}$] in the presence of PhCl but the absence of a radical trap, where significant decomposition was observed for **DCA** (*vide supra*) but not for **NpMI** (see Figures 3b and 4b). Nevertheless, regeneration of **NpMI** is clearly less than quantitative in some – though not all - cases.

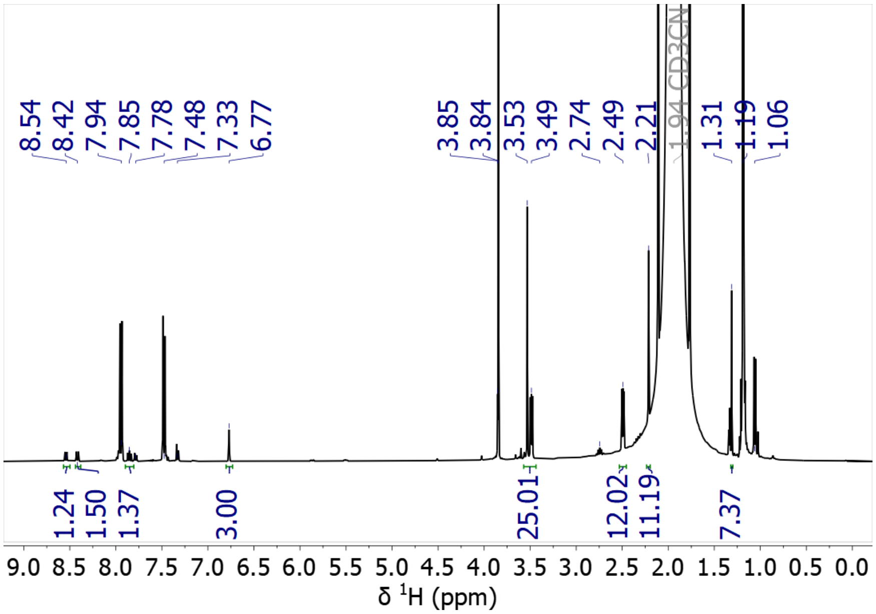

Figure S51. ^1H NMR spectrum of the products of the reaction of [K(crypt)$^+$][**NpMI$^{•-}$**] with $MeO_2C(C_6H_4)Cl$ and $P(OMe)_3$, following irradiation at 455 nm for 16 h. Resonances assigned to **NpMI** (δ = 8.54, 8.42, 7.86 and 1.06 ppm) are integrated relative to 1 eq. mesitylene as an internal standard and indicate good recovery of the neutral **NpMI** (69%).

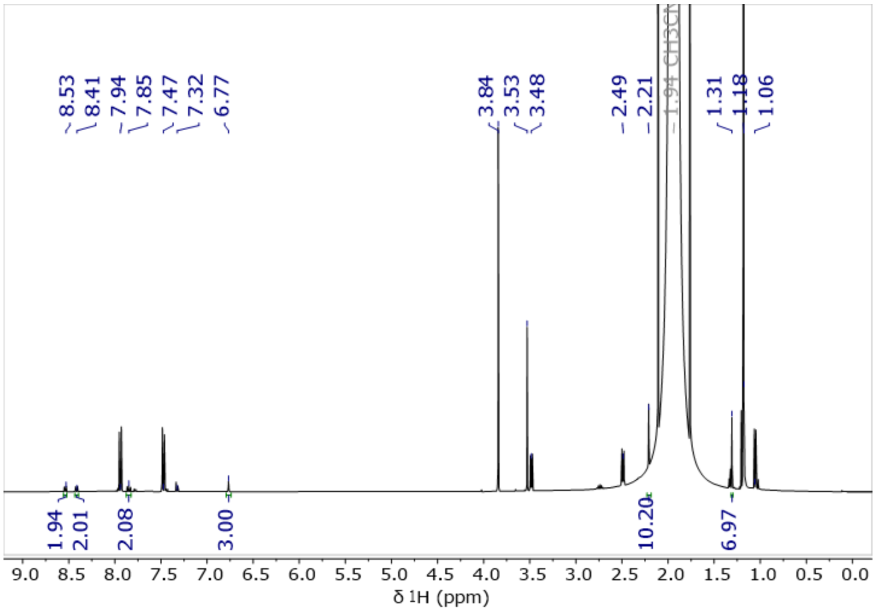

Figure S52. ^1H NMR spectrum of the products of the reaction of [K(crypt)$^+$][**NpMI**$^{\bullet-}$] with $MeO_2C(C_6H_4)Cl$ and B_2pin_2, following irradiation at 530 nm for 16 h. Resonances assigned to **NpMI** (8.53, 8.41, 7.85 and 1.06 ppm) are integrated relative to 1 eq. mesitylene as an internal standard and indicate excellent recovery of the neutral **NpMI** (*ca.* 100%).

Interestingly, in some model reactions the reformation of neutral **NpMI** could not be observed directly by ^1H NMR spectroscopic analysis of the final reaction mixture. For example, in the spectrum acquired for the reaction between PhCl and $P(OMe)_3$ at 530 nm, no aromatic resonances attributable to **NpMI** were detected (Figure S53). Similar results were obtained upon irradiation of [K(crypt)$^+$][**NpMI**$^{\bullet-}$] and PhCl for 16 h at 530 nm in the absence of a radical trap (Figure S54; *c.f.* Figure 4b).

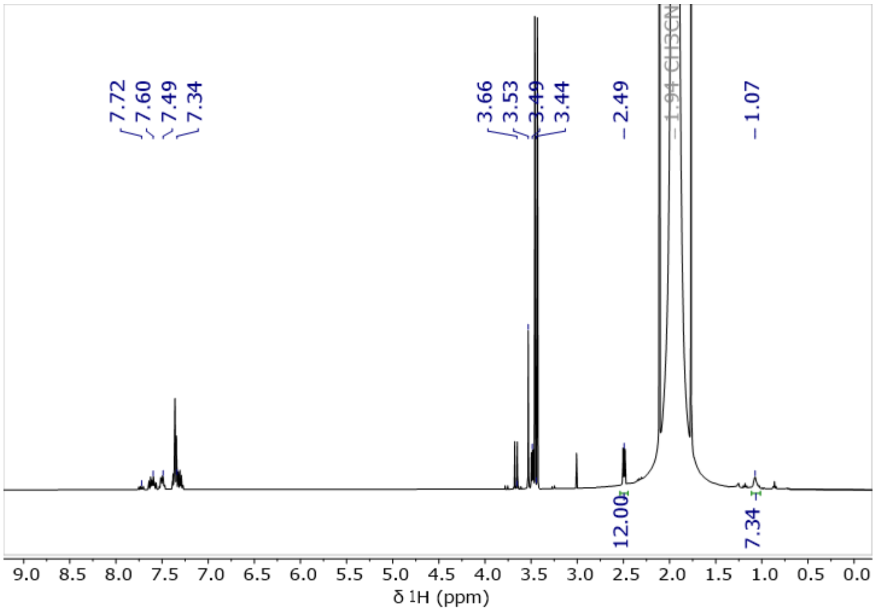

Figure S53. ^{1}H NMR spectrum of the products of the reaction of [K(crypt)$^+$][**NpMI**$^{\bullet-}$] with PhCl and P(OMe)$_3$, following irradiation at 530 nm for 16 h. No resonances assignable to **NpMI** are apparent.

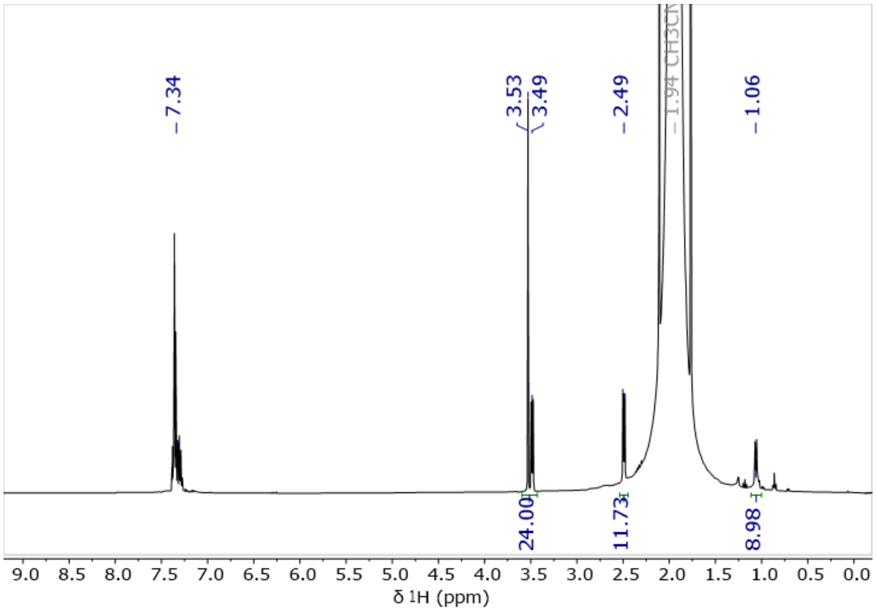

Figure S54. ^{1}H NMR spectrum for the reaction of [K(crypt)$^+$][**NpMI**$^{\bullet-}$] with PhCl following irradiation at 530 nm for 16 h. No resonances assignable to **NpMI** are apparent.

These initially surprising observations are attributed to the presence of sufficient quantities of residual, anionic **NpMI**$^{\bullet-}$ in these reaction mixtures. To support this conclusion an equimolar mixture of [K(crypt)$^{+}$][**NpMI**$^{\bullet-}$] and neutral **NpMI** was prepared in MeCN (5 mM each) and analysed by ^{1}H NMR spectroscopy. As expected, no sharp resonances attributable to **NpMI** were observed in the aromatic region (Figure S55).

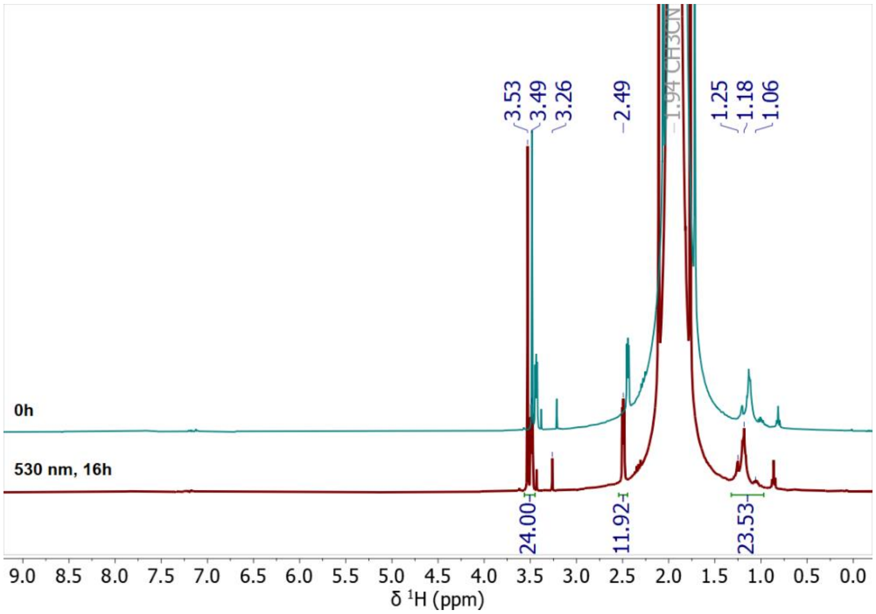

Figure S55. ^{1}H NMR spectra of NpMI^{0}/[K(crypt)$^{+}$][**NpMI**$^{\bullet-}$] mixture before and after irradiation with 530 nm light for 16 h. Assignments: **NpMI**^{0}/[**NpMI**$^{\bullet-}$], 1.06-1.25 ppm; [K(crypt)$^{+}$], 3.53, 3.49, 2.49 ppm. No sharp aromatic resonances attributable to neutral **NpMI** are assignable.

Clearly, there is an interaction between the neutral and anionic forms of **NpMI** that renders the former NMR-silent. In isolation, this observation could plausibly be consistent with stoichiometric formation of an EDA-type complex, or even with direct adduct formation. However, no change in the UV-vis absorption spectrum is apparent upon addition of **NpMI**^{0} to **NpMI**$^{\bullet-}$, which seems inconsistent with these interpretations (Figure S56). Instead, a simple, degenerate electron-transfer between **NpMI** and **NpMI**$^{\bullet-}$, occurring rapidly on the NMR timescale, is suggested as a preferred explanation.

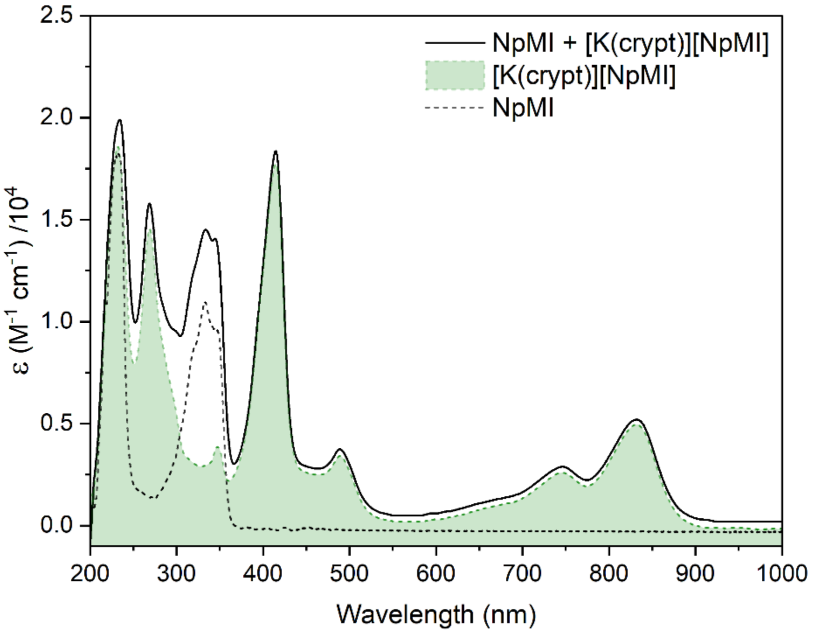

Figure S56. UV-visible absorption spectra of [K(crypt)$^+$][**NpMI**$^{•-}$] (1 mM), **NpMI**^{0} (1 mM) and a 1:1 mixture of [K(crypt)$^+$][**NpMI**$^{•-}$] and **NpMI**^{0} (1 mM each).

Further evidence for the above interpretation was provided by deliberately exposing the reaction mixture whose ^{1}H NMR spectrum is shown in Figure S53 to air, to allow oxidation of any residual **NpMI**$^{•-}$. Subsequent NMR re-analysis clearly showed the expected resonances for neutral **NpMI** (Figure S57).

Note that, in principle, neutral **NpMI**^{0} could also be (re)generated by exposure of [**NpMI**•H$^-$] to air.[5] However, this possibility is ruled out by the absence of resonances attributable to [**NpMI**•H$^-$] in the ^{1}H NMR spectrum prior to exposure (Figure S53).

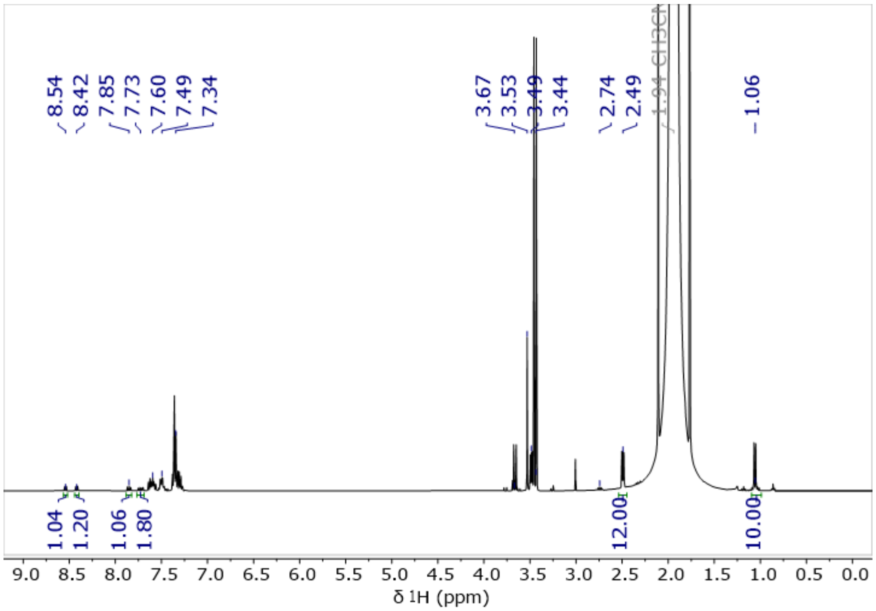

Figure S57. ^{1}H NMR spectrum of the products of the reaction of [K(crypt)$^{+}$][**NpMI**$^{•-}$] with PhCl and $P(OMe)_3$, following irradiation at 530 nm for 16 h and subsequent exposure to air. Resonances assignable to **NpMI** can be observed at 8.54, 8.42, 7.85 and 1.06 ppm.

4. Supplementary mechanistic discussion

As alluded to in the main manuscript, while this report provides significant experimental evidence consistent with the doublet pathway originally proposed for conPET and e-PRC reactivity, this alone does not afford definitive proof that other mechanistic pathways cannot also be available in such reactions. This is particularly true of reactions previously reported in the literature, which may feature more complex reaction mixtures and/or less rigorous exclusion of air and moisture than those described herein.

A truly comprehensive comparison of the doublet pathway with all possible alternatives is unfortunately beyond the scope of this report, especially since any competition between such mechanisms is likely to be highly reaction specific. Nevertheless, for the sake of context it is instructive to briefly consider a few of these alternative possibilities, and particularly whether they could be relevant to the reactivity observed during this study. Note that our own, more targeted investigations of these questions are ongoing and will be reported in due course.

4.1. Possible formation of [NpMI•H⁻] or other closed-shell species prior to substrate reduction

In light of the recent work of Nocera *et al.*,[5] highlighted in Scheme 4, perhaps the most obvious alternative mechanistic possibility is the involvement of [**NpMI**•H^-] as an intermediate, formed *in situ* from **NpMI**$^{\bullet-}$. As such, it is worth emphasising that we did not notice any evidence for formation of [**NpMI**•H^-] at any point during our study (for example by observation of the ^{1}H NMR resonances that would be expected at *ca.* 4.1-4.2 ppm). The intermediacy of [**NpMI**•H^-] specifically would also seem to be ruled out, at least under our conditions, by the observation of reactivity under 730 nm irradiation: a wavelength well beyond the window within which [**NpMI**•H^-] is reported to absorb significantly.[5]

More broadly, the intermediacy of either [**NpMI**•H^-] or other closed-shell species, formed from PC$^{\bullet-}$ as a *necessary* reaction step, *prior to substrate reduction*, seems unlikely due to the following observations, at least for the reactions reported herein:

1. As shown in section 3.1, the salts [K(crypt)$^+$][PC$^{\bullet-}$] (PC = **DCA**, **NpMI**) are photostable under LED irradiation under the conditions used in this study.* In other words, PC$^{\bullet-}$ does not undergo a photoreaction in the absence of substrate.**
2. As shown in section 3.3, the salts [K(crypt)$^+$][PC$^{\bullet-}$] (PC = **DCA**, **NpMI**) do not react with the aryl chloride substrates used in this study in the dark. In other words, PC$^{\bullet-}$ does not undergo a reaction with substrate in its electronic ground state.

3. Of the initial reaction components, only [K(crypt)$^+$][PC$^{•−}$] absorbs appreciably in the visible region of the UV-vis spectrum. In other words, only PC$^{•−}$ is susceptible to photoexcitation in the initial reaction mixture.
4. As shown in section 3.4, the salts [K(crypt)$^+$][PC$^{•−}$] (PC = **DCA**, **NpMI**) *do* react with the aryl chloride substrates used in this study under LED irradiation at visible wavelengths. Given that no other photoactive species are present at the start of the reaction, and that no reactivity is observed without photoexcitation, this observed reactivity *must* involve initial photoexcitation of PC$^{•−}$. The fact that photoexcitation of PC$^{•−}$ does not appear to induce reactivity towards any other species present at the start of the reaction suggests that the reaction can then only proceed further *via* reaction of photoexcited PC$^{•−}$ (i.e. *PC$^{•−}$) with the aryl chloride substrate.**

   In other words, these observations suggest that the originally proposed doublet conPET/e-PRC mechanism, in which the substrate is transformed through direct reaction with electronically excited PC$^{•−}$, must take place at the *start* of the reaction.

Note that as already stated, while these arguments apply to the reactions reported herein, they cannot necessarily be extrapolated to all reactions by other researchers reported previously in the literature. However, for a critical discussion of the possible role of [**NpMI**•H$^-$] in those processes, readers are referred to a recent review article by Mandigma, Kaur and Barham.[8]

* *(With the sole exception of K(crypt)$^+$][**NpMI**$^{•−}$] under 455 nm LEDs. As discussed in section 3.1.2, this means that results with this combination of PC$^{•−}$ and wavelength should be interpreted with somewhat more caution. However, since this limitation does not extend to other combinations, including those for which reactivity is also observed, the overall argument remains.)*

** *(An alternative possibility that cannot be formally excluded is that PC$^{•−}$ <u>does</u> undergo a transformation upon photoirradiation, but the resulting photoproduct is unstable and rapidly reverts to PC$^{•−}$ afterwards, as such a product would be difficult to detect by steady-state spectroscopic analysis.)*

4.2. Possible formation of other closed-shell species after initial substrate reduction

As already discussed in section 3.5.1, while the **DCA**$^{•−}$ radical anion is photostable on its own in solution, the **DCA** moiety decomposes substantially during typical reactions. This is attributed to attack of aryl radicals on remaining **DCA**$^{•−}$ following initial reduction of the substrate (see Scheme S1, above).[7] While the **NpMI** moiety appears to be substantially more robust, some degradation of this motif is also implied by the results shown in section 3.5.2, at least in certain cases.

Although they have not yet been fully identified, it is likely that many of the products of this PC/PC•− degradation will remain structurally – and hence electronically – similar to the initial PC/PC•− (e.g. Scheme S1). Hence, it is plausible that some of these products may themselves be photochemically active. This could potentially be relevant to the overall outcome of some of the reactions reported herein, if these new species are themselves capable of interacting with the reaction substrates in their excited states. However, it should be emphasised that any mechanistic involvement of these decomposition products *must occur downstream of an initial substrate reduction step that does not involve these decomposition products*, for which the arguments summarised in section 4.1, above, will apply. In other words, any transformation proceeding *via* such a pathway still requires initiation *via* photoexcitation of PC•− leading to Ar-Cl reduction (Scheme S2).

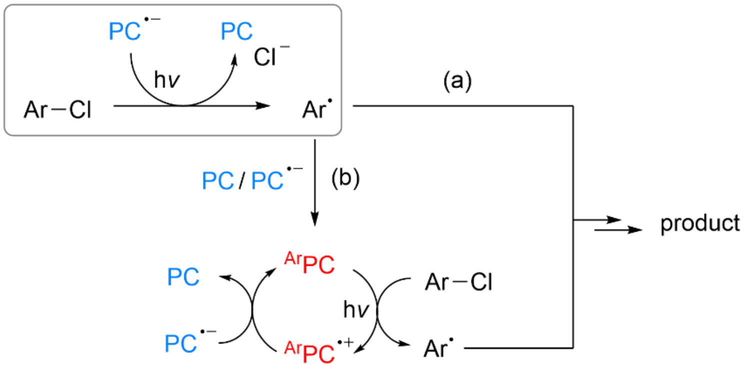

Scheme S2. Possible mechanisms for aryl chloride reduction either stoichiometrically mediated (a) or initiated (b) *via* photoexcitation of PC•−. Both mechanisms require that reduction of substrate *via* photoexcitation of PC•− be viable in order to be feasible.

Though not conclusive, some *qualitative* observations suggest that such an initiation pathway need not be significant. First, as shown in section 3.5.2 (e.g. Figure 52), some reactions using **NpMI•−** result in essentially quantitative regeneration of **NpMI⁰** by reaction's end, indicating that reactivity can be achieved without appreciable degradation of the backbone **NpMI** motif. Second, as shown in section 3.4.4, significantly higher reaction turnover with respect to PC•− is observed when the PC•− concentration is lowered. This can easily be rationalised if Ar• attack on PC/PC•− is in fact simply a catalyst deactivation pathway, as by reducing [PC•−] concentration this pathway should be kinetically disfavoured relative to the productive Ar• trapping pathway.

4.3. Possible oxidation of PC•− by O_2

While the radical anion salts [K(crypt)+][PC•−] (PC = **DCA**, **NpMI**) are stable under inert atmosphere, both as solids and in solution, they are highly sensitive to O_2 and show immediate colour changes upon exposure to

air. Previous *in situ* studies have shown that for **DCA**$^{\bullet-}$ such oxidation does not simply regenerate neutral **DCA**, but rather leads to products such as 10-cyanoanthrolate.[9] Notably, the König group have shown that related anthrolates can be active and highly reducing photocatalysts, albeit mostly using more electron-rich examples.[10] However, it should be noted that 10-cyanoanthrolate is not reported to absorb significantly in the UV-vis region above *ca*. 550 nm, and so could not easily account for reactivity observed at longer wavelengths (e.g. 630nm, 730 nm).[11]

The analogous oxidation of **NpMI**$^{\bullet-}$ has not been studied in detail to our knowledge, but again exposure of samples to air does not cleanly regenerate the neutral form, as illustrated by UV-vis spectroscopy (Figure S58), suggesting similar "structural" oxidation.

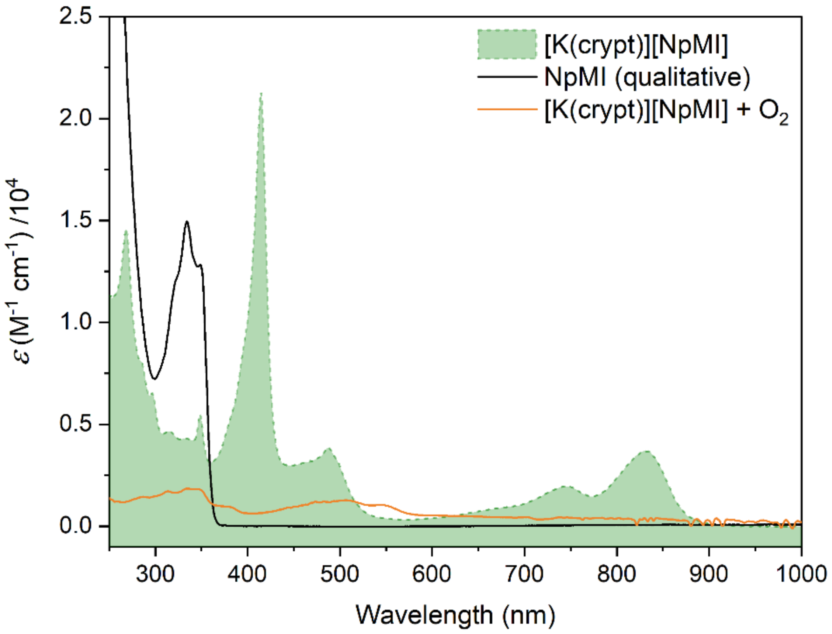

Figure S58. UV-vis absorption spectra of [K(crypt)$^+$][**NpMI**$^{\bullet-}$], recorded on 504 μM MeCN solutions before and after exposure to air. The spectra were recorded using a 1 mm path length cuvette.

To prevent *in situ* oxidation of PC$^{\bullet-}$ to other, potentially photoactive motifs, all experiments in this study were performed under a rigorous inert atmosphere, by using a glovebox (with < 0.1 ppm O_2, H_2O) to prepare samples in flame-dried NMR tubes with vacuum-tested J. Youngs taps, using thoroughly dried and degassed solvents and reagents and analytically pure PC$^{\bullet-}$ salts. However, it is notable that such extensive exclusion of air has not been a feature of many previously reported conPET and e-PRC reactions. In these cases, the mechanistic relevance of PC$^{\bullet-}$ oxidation products cannot necessarily be discounted.

4.4. Possible substrate/PC$^{\bullet-}$ pre-assembly

While the results described in this study suggest that electron transfer can occur between aryl chloride substrates and electronically excited PC$^{\bullet-}$, *how* this could occur remains an open question, particularly in light of the short lifetimes previously assigned to the relevant *PC$^{\bullet-}$ excited states. One relatively simple explanation, which has been invoked in the context of *oxidative* e-PRC reactions,[12] is that preassembly might occur between the substrate and PC$^{\bullet-}$, such that electron transfer following photoexcitation is not limited by diffusion.

Given the reactivity involved, such a pre-assembly could be imagined as taking the form of an electron donor-acceptor (EDA) interaction, albeit involving a relatively electron-rich acceptor. However, other, weaker noncovalent interactions could also be responsible. As an initial probe of this possibility, it has been found that the UV-vis spectra of [K(crypt)$^{+}$][PC$^{\bullet-}$] do not shift or otherwise change substantially in the presence of catalytically-relevant concentrations of the aryl chloride substrates (see section 3.2, above). These experiments therefore do not point towards strong EDA complex formation. Nevertheless, weaker – and hence harder to detect – preassembly remains a possibility and need not necessarily induce significant changes by UV-vis spectroscopy, as has been emphasised in a recent study by Hauer *et al.*[13] Ultimately, confirmation or falsification of preassemblies can only be confirmed by transient absorption spectroscopy, and studies in this direction are ongoing.

5. X-ray crystallographic details

Crystallographic data were collected on a SuperNova single-source diffractometer (Atlas) at 123 K ([K(crypt)$^+$][**DCA**$^{\bullet-}$]) or a Rigaku XtaLAB Synergy R diffractometer with a HyPix-Arc 150 detector ([K(crypt)$^+$][**NpMI**$^{\bullet-}$]) at the Universität Regensburg.

CCDC 2237812 ([K(crypt)$^+$][**DCA**$^{\bullet-}$]) and 2237813 ([K(crypt)$^+$][**NpMI**$^{\bullet-}$]•(THF)) contain the supplementary crystallographic data for this paper. These can be obtained free of charge from the Cambridge Crystallographic Data Centre.

5.1. XRD data for [K(crypt)$^+$][DCA$^{\bullet-}$]

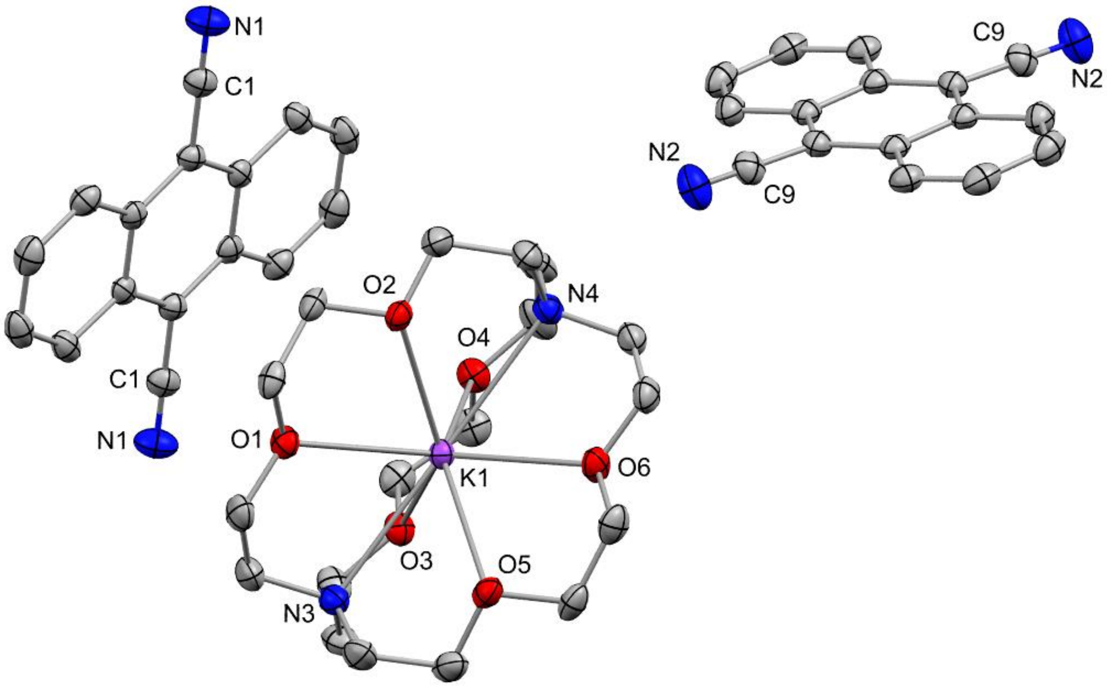

Figure S59. Solid-state molecular structure of [K(crypt)$^+$][**DCA**$^{\bullet-}$]. Purple plate-like crystals were grown from a THF/hexane solution at -35 °C. Thermal ellipsoids are drawn at 50% probability. H atoms are omitted for clarity. The asymmetric unit comprises 1x [K(crypt)$^+$] and 2x 0.5 [**DCA**$^{\bullet-}$], which are non-equivalent, and have each been expanded to show the full anions for clarity. Full bond distance information may be found in the crystallographic tables (*vide infra*). Atoms are coloured as follows: C, grey; N, blue; O, red; K, purple.

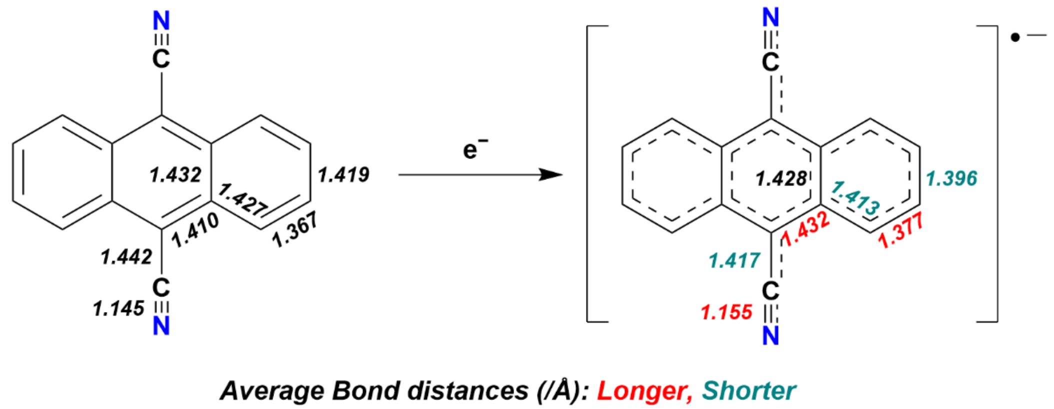

Figure S60. Average bond distances in **DCA**^{0} (LHS)[14] and [**DCA**$^{\bullet-}$] (RHS). The colours indicate whether the bond is longer (red) or shorter (cyan) in the radical anion compared to the neutral molecule, illustrating the pattern of lengthening/shortening expected from the nodal MO structure.

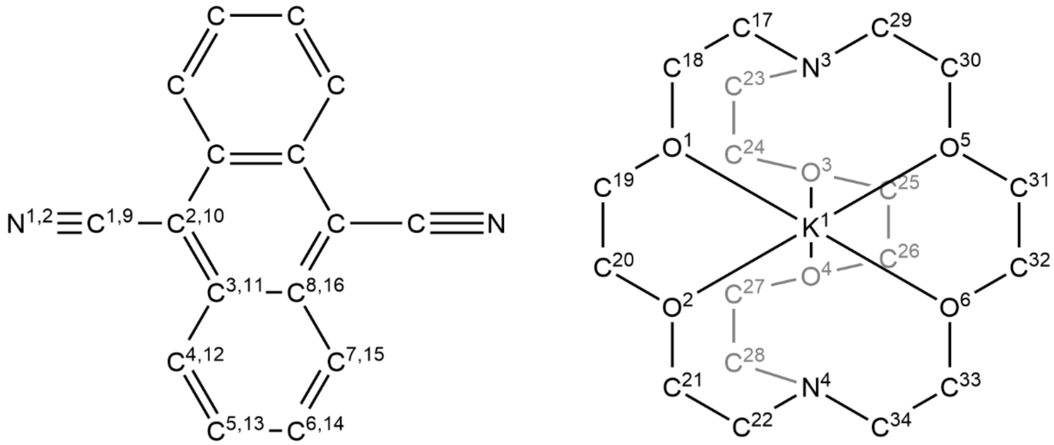

Figure S61. Atomic numbering in [K(crypt)$^+$][**DCA**$^{\bullet-}$]

Table S4. Crystallographic data and structure refinement details for [K(crypt)$^+$][**DCA**$^{\bullet-}$]

**Empirical formula**	**$C_{34}H_{44}N_4O_6K$**
Formula weight	643.83
Temperature/K	123.00(10)
Crystal system	monoclinic
Space group	$P2_1/c$
a/Å	15.9812(2)
b/Å	17.3429(2)
c/Å	12.4838(2)
α/°	90
β/°	105.1530(10)
γ/°	90
Volume/Å^3	3339.71(8)
Z	4
ρ_{calc}g/cm^3	1.280
μ/mm^{-1}	1.797
F(000)	1372.0
Crystal size/mm^3	0.728 × 0.232 × 0.081
Radiation	Cu Kα (λ = 1.54184)
2Θ range for data collection/°	7.67 to 147.236
Index ranges	-18 ≤ h ≤ 19, -20 ≤ k ≤ 21, -15 ≤ l ≤ 14
Reflections collected	25374
Independent reflections	6614 [R_{int} = 0.0270, R_{sigma} = 0.0194]
Data/restraints/parameters	6614/0/406
Goodness-of-fit on F^2	1.030
Final R indexes [I>=2σ (I)]	R_1 = 0.0286, wR_2 = 0.0761
Final R indexes [all data]	R_1 = 0.0298, wR_2 = 0.0772
Largest diff. peak/hole / e Å^{-3}	0.23/-0.21

Table S5. Tabulated bond distances in [K(crypt)+][**DCA•−**]

Bond		Length/Å	Bond		Length/Å	Bond		Length/Å
K1	O2	2.8418(8)	O5	C30	1.4256(14)	C11	C10	1.4333(14)
K1	O1	2.8137(7)	N3	C29	1.4707(15)	C12	C13	1.3775(16)
K1	O3	2.7904(8)	N3	C17	1.4670(14)	C10	C9	1.4171(16)
K1	O4	2.8612(8)	N3	C23	1.4689(15)	C4	C5	1.3757(16)
K1	O6	2.7551(7)	N4	C34	1.4718(14)	C13	C14	1.3965(19)
K1	O5	2.9029(8)	N4	C22	1.4680(14)	C20	C19	1.4945(16)
K1	N3	2.9866(9)	N4	C28	1.4696(15)	C7	C6	1.3771(17)
K1	N4	2.9731(9)	N1	C1	1.1557(15)	C5	C6	1.3951(18)
O2	C20	1.4257(12)	C3	C2	1.4316(14)	C34	C33	1.5063(17)
O2	C21	1.4219(14)	C3	C8	1.4286(14)	C14	C15	1.3784(17)
O1	C19	1.4214(13)	C3	C4	1.4156(14)	C31	C32	1.4929(18)
O1	C18	1.4178(13)	C16	C11	1.4283(15)	C22	C21	1.5103(16)
O3	C24	1.4199(14)	C16	C10	1.4301(15)	C18	C17	1.5070(17)
O3	C25	1.4204(14)	C16	C15	1.4120(15)	C30	C29	1.5049(17)
O4	C26	1.4187(14)	C2	C8	1.4334(14)	C24	C23	1.5070(18)
O4	C27	1.4153(14)	C2	C1	1.4170(14)	C25	C26	1.4924(17)
O6	C33	1.4260(14)	C8	C7	1.4127(15)	C28	C27	1.5038(18)
O6	C32	1.4215(13)	N2	C9	1.1548(16)			
O5	C31	1.4234(13)	C11	C12	1.4115(15)			

Table S6. Tabulated bond angles in [K(crypt)+][**DCA•−**]

Atoms			Angle/°	Atoms			Angle/°
O2	K1	O4	98.64(2)	C34	N4	K1	106.42(6)
O2	K1	O5	113.30(2)	C22	N4	K1	108.95(6)
O2	K1	N3	119.02(2)	C22	N4	C34	110.97(9)
O2	K1	N4	61.06(2)	C22	N4	C28	109.71(9)
O1	K1	O2	60.38(2)	C28	N4	K1	110.65(6)
O1	K1	O4	118.36(2)	C28	N4	C34	110.07(9)
O1	K1	O5	94.49(2)	C8	C3	C2	119.05(9)
O1	K1	N3	60.02(2)	C4	C3	C2	122.62(9)
O1	K1	N4	119.93(2)	C4	C3	C8	118.33(9)
O3	K1	O2	139.24(2)	C11	C16	C101	119.09(9)
O3	K1	O1	97.78(2)	C15	C16	C11	118.24(10)
O3	K1	O4	59.67(2)	C15	C16	C101	122.67(10)
O3	K1	O5	101.62(2)	C3	C2	C82	121.83(9)
O3	K1	N3	61.23(2)	C1	C2	C3	118.93(9)
O3	K1	N4	118.31(2)	C1	C2	C82	119.24(9)
O4	K1	O5	142.88(2)	C3	C8	C22	119.12(9)

O4	K1	N3	119.77(2)	C7	C8	C3	118.42(9)
O4	K1	N4	59.71(2)	C7	C8	C22	122.46(9)
O6	K1	O2	94.37(2)	C16	C11	C10	118.75(9)
O6	K1	O1	135.03(2)	C12	C11	C16	118.74(9)
O6	K1	O3	121.93(2)	C12	C11	C10	122.50(10)
O6	K1	O4	100.76(2)	C13	C12	C11	121.36(11)
O6	K1	O5	59.93(2)	N1	C1	C2	179.79(14)
O6	K1	N3	119.35(3)	C161	C10	C11	122.16(10)
O6	K1	N4	61.11(2)	C9	C10	C161	119.94(10)
O5	K1	N3	60.61(2)	C9	C10	C11	117.76(10)
O5	K1	N4	119.91(2)	C5	C4	C3	121.69(10)
N4	K1	N3	179.44(3)	C12	C13	C14	120.06(10)
C20	O2	K1	111.53(6)	O2	C20	C19	110.43(9)
C21	O2	K1	116.57(6)	C6	C7	C8	121.49(11)
C21	O2	C20	109.77(8)	O1	C19	C20	108.88(8)
C19	O1	K1	116.65(6)	C4	C5	C6	119.82(10)
C18	O1	K1	120.40(6)	N4	C34	C33	114.60(9)
C18	O1	C19	111.77(8)	N2	C9	C10	177.69(12)
C24	O3	K1	119.07(6)	C15	C14	C13	119.97(10)
C24	O3	C25	110.74(9)	C14	C15	C16	121.61(11)
C25	O3	K1	116.99(6)	O5	C31	C32	110.53(9)
C26	O4	K1	114.43(6)	O6	C33	C34	109.07(9)
C27	O4	K1	119.50(6)	C7	C6	C5	120.24(10)
C27	O4	C26	111.20(9)	N4	C22	C21	113.54(9)
C33	O6	K1	121.76(6)	O1	C18	C17	109.15(9)
C32	O6	K1	119.50(6)	O5	C30	C29	109.27(9)
C32	O6	C33	111.82(8)	N3	C29	C30	114.09(9)
C31	O5	K1	110.68(6)	O3	C24	C23	109.57(9)
C31	O5	C30	109.76(8)	O6	C32	C31	109.32(9)
C30	O5	K1	114.00(6)	O3	C25	C26	110.05(9)
C29	N3	K1	109.99(7)	N3	C17	C18	113.84(9)
C17	N3	K1	109.38(6)	O2	C21	C22	109.88(9)
C17	N3	C29	110.24(9)	O4	C26	C25	109.34(9)
C17	N3	C23	109.72(9)	N3	C23	C24	114.72(10)
C23	N3	K1	107.48(6)	N4	C28	C27	114.18(10)
C23	N3	C29	109.99(9)	O4	C27	C28	109.24(10)

5.2. XRD data for [K(crypt)+][NpMI•−]•(THF)

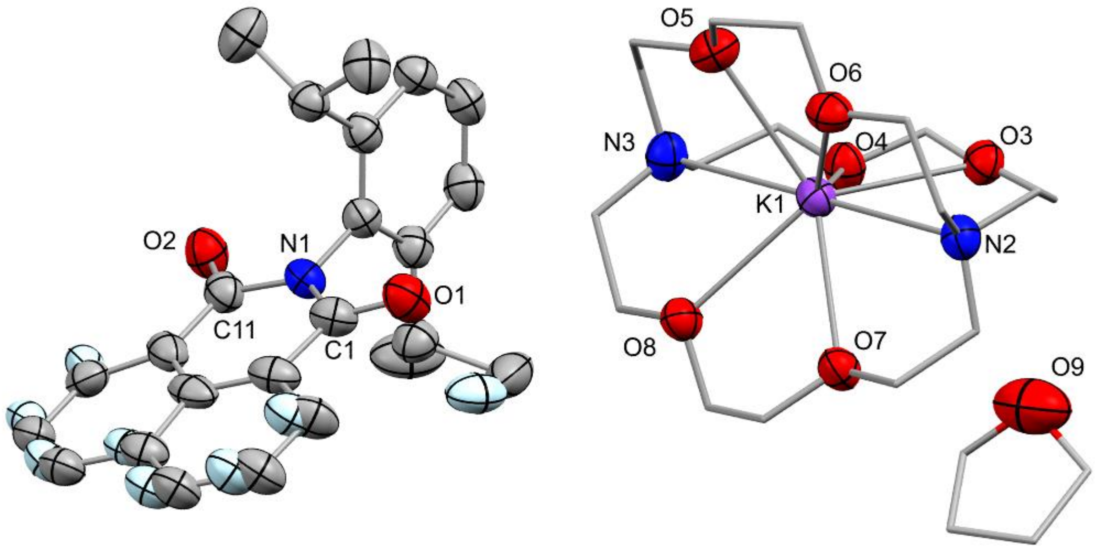

Figure S62. Solid-state molecular structure of [K(crypt)+][**NpMI•−**]•(THF). Clear brown plate-like crystals were grown from a THF/Et_2O solution at -35 °C. Thermal ellipsoids are drawn at 50% probability. C atoms of [K(crypt)+] and THF molecule are drawn in wireframe and all H atoms are omitted for clarity. The naphthalene moiety and one isopropyl group of [**NpMI•−**] exhibit some disorder with C atoms over two sites with 0.57 (light blue) and 0.43 (grey) occupancy. Full bond distance information can be found in the crystallographic tables (*vide infra*). Atoms are coloured as follows: C, grey (disorder: light blue); N, blue; O, red; K, purple.

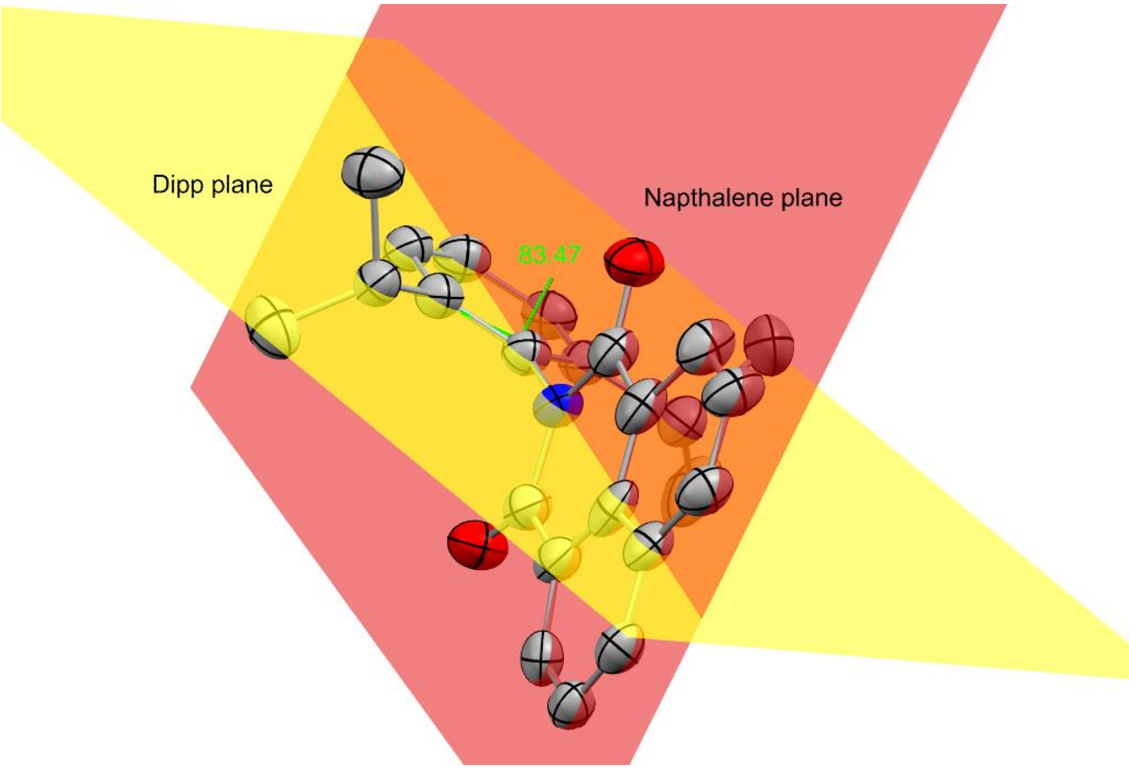

Figure S63. Illustration showing the intersection of the planes described by the Dipp ring (yellow) and the naphthalene moiety (red). The angle of intersection was determined to be 83.47°. The Dipp plane was calculated from all six aromatic carbons of the Dipp group, while the naphthalene plane was calculated from the six atoms of the N-heterocyclic ring.

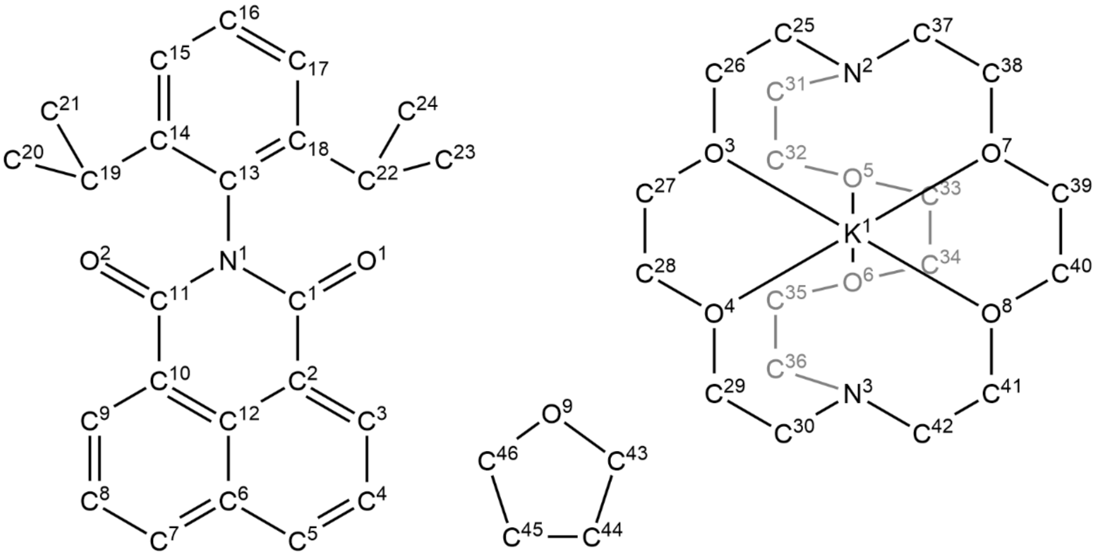

Figure S64. Atomic numbering in [K(crypt)$^{+}$][**NpMI$^{\bullet-}$**]•(THF)

Table S7. Crystallographic data and structure refinement details for [K(crypt)$^{+}$][**NpMI**$^{\bullet-}$]•(THF)

**Empirical formula**	$C_{46}H_{67}KN_3O_9$
Formula weight	845.12
Temperature/K	123.00(10)
Crystal system	monoclinic
Space group	P21/c
a/Å	14.6620(2)
b/Å	22.6711(4)
c/Å	15.7634(2)
α/°	90
β/°	91.9340(10)
γ/°	90
Volume/Å^3	5236.83(13)
Z	4
ρ_{calc}g/cm^3	1.072
μ/mm^{-1}	1.286
F(000)	1820.0
Crystal size/mm^3	0.283 × 0.176 × 0.037
Radiation	Cu Kα (λ = 1.54184)
2Θ range for data collection/°	6.032 to 143.204
Index ranges	-12 ≤ h ≤ 17, -27 ≤ k ≤ 25, -19 ≤ l ≤ 19
Reflections collected	49068
Independent reflections	9893 [R_{int} = 0.0338, R_{sigma} = 0.0232]
Data/restraints/parameters	9893/204/611
Goodness-of-fit on F^2	1.103
Final R indexes [I>=2σ (I)]	R_1 = 0.0699, wR_2 = 0.1999
Final R indexes [all data]	R_1 = 0.0797, wR_2 = 0.2093
Largest diff. peak/hole / e Å^{-3}	0.37/-0.80

Table S8. Tabulated bond distances in [K(crypt)+][**NpMI**•−]•(THF)

Bond		Length/Å	Bond		Length/Å	Bond		Length/Å
K1	O7	2.822(2)	N2	C36	1.468(4)	C22	C24B	1.485(12)
K1	O8	2.8393(19)	N3	C31	1.471(4)	C26	C25	1.513(4)
K1	O3	2.8034(19)	N3	C42	1.472(4)	C31	C32	1.507(5)
K1	O6	2.841(2)	N3	C30	1.474(4)	C37	C38	1.499(5)
K1	O4	2.823(2)	C18	C13	1.402(4)	C36	C35	1.504(4)
K1	O5	2.829(2)	C18	C17	1.390(4)	C27	C28	1.487(4)
K1	N2	2.945(2)	C18	C22	1.520(4)	C39	C40	1.483(5)
K1	N3	2.976(2)	C19	C14	1.520(4)	C6B	C7B	1.411(16)
O7	C34	1.416(3)	C19	C20	1.523(4)	C6B	C5B	1.407(17)
O7	C35	1.426(3)	C19	C21	1.517(4)	C42	C41	1.505(5)
O8	C33	1.423(3)	C13	C14	1.390(4)	C29	C30	1.507(5)
O8	C32	1.425(3)	C11	C10	1.435(4)	C9B	C8B	1.40(2)
O3	C26	1.425(3)	C14	C15	1.397(4)	C7B	C8B	1.374(13)
O3	C27	1.428(3)	C17	C16	1.377(4)	C3B	C4B	1.35(3)
O6	C38	1.430(4)	C1	C2	1.443(4)	C5B	C4B	1.390(14)
O6	C39	1.420(4)	C10	C12	1.413(4)	O9	C46	1.390(6)
O4	C28	1.415(4)	C10	C9B	1.37(3)	O9	C43	1.399(7)
O4	C29	1.426(4)	C10	C9A	1.471(18)	C46	C45	1.521(7)
O2	C11	1.240(3)	C15	C16	1.377(4)	C44	C45	1.497(8)
O5	C40	1.426(4)	C12	C2	1.411(4)	C44	C43	1.475(8)
O5	C41	1.421(4)	C12	C6B	1.45(2)	C4A	C5A	1.404(10)
O1	C1	1.247(3)	C12	C6A	1.444(17)	C4A	C3A	1.411(19)
N1	C13	1.453(3)	C2	C3B	1.49(3)	C5A	C6A	1.394(13)
N1	C11	1.414(3)	C2	C3A	1.36(2)	C6A	C7A	1.424(11)
N1	C1	1.402(3)	C34	C33	1.488(4)	C7A	C8A	1.379(10)
N2	C25	1.468(4)	C22	C23	1.494(4)	C8A	C9A	1.375(14)
N2	C37	1.473(4)	C22	C24A	1.567(9)			

Table S9. Tabulated bond angles in [K(crypt)+][**NpMI•−**]•(THF)

Atoms			Angle/°	Atoms			Angle/°
O7	K1	O8	59.70(5)	N1	C11	C10	116.2(2)
O7	K1	O6	99.42(6)	C13	C14	C19	122.5(2)
O7	K1	O4	115.36(6)	C13	C14	C15	117.6(2)
O7	K1	O5	140.66(6)	C15	C14	C19	119.9(2)
O7	K1	N2	61.20(6)	C16	C17	C18	121.2(3)
O7	K1	N3	118.56(6)	O1	C1	N1	118.9(2)
O8	K1	O6	117.42(6)	O1	C1	C2	124.6(3)
O8	K1	N2	119.12(6)	N1	C1	C2	116.4(2)
O8	K1	N3	60.30(6)	C11	C10	C9A	114.0(6)
O3	K1	O7	99.05(6)	C12	C10	C11	120.4(2)
O3	K1	O8	141.08(6)	C12	C10	C9A	125.6(6)
O3	K1	O6	96.92(6)	C9B	C10	C11	128.0(8)
O3	K1	O4	60.97(6)	C9B	C10	C12	111.5(8)
O3	K1	O5	115.22(6)	C16	C15	C14	121.2(3)
O3	K1	N2	61.53(6)	C17	C16	C15	120.1(3)
O3	K1	N3	119.84(6)	C10	C12	C6B	128.6(7)
O6	K1	N2	60.25(6)	C10	C12	C6A	112.8(6)
O6	K1	N3	118.63(7)	C2	C12	C10	121.2(2)
O4	K1	O8	96.91(6)	C2	C12	C6B	110.2(7)
O4	K1	O6	140.47(6)	C2	C12	C6A	126.0(6)
O4	K1	O5	98.55(6)	C1	C2	C3B	113.4(11)
O4	K1	N2	120.53(6)	C12	C2	C1	120.2(3)
O4	K1	N3	60.93(6)	C12	C2	C3B	126.3(11)
O5	K1	O8	98.61(6)	C3A	C2	C1	124.4(8)
O5	K1	O6	59.46(6)	C3A	C2	C12	115.3(8)
O5	K1	N2	118.41(7)	O7	C34	C33	109.9(2)
O5	K1	N3	60.73(7)	C18	C22	C24A	107.7(4)
N2	K1	N3	178.53(7)	C23	C22	C18	114.0(3)
C34	O7	K1	116.18(15)	C23	C22	C24A	100.0(8)
C34	O7	C35	110.9(2)	C24B	C22	C18	112.8(5)
C35	O7	K1	117.70(17)	C24B	C22	C23	120.6(7)
C33	O8	K1	114.67(15)	O3	C26	C25	109.6(2)
C33	O8	C32	110.6(2)	N2	C25	C26	114.4(2)
C32	O8	K1	119.89(17)	O8	C33	C34	109.8(2)
C26	O3	K1	118.33(16)	N3	C31	C32	114.7(2)
C26	O3	C27	110.1(2)	N2	C37	C38	113.7(3)
C27	O3	K1	113.71(16)	N2	C36	C35	113.9(2)
C38	O6	K1	119.58(16)	O3	C27	C28	110.2(2)
C39	O6	K1	114.59(16)	O6	C38	C37	110.1(2)
C39	O6	C38	111.0(2)	O6	C39	C40	110.1(3)
C28	O4	K1	114.25(17)	O7	C35	C36	109.7(2)

C28	O4	C29	111.2(2)	O8	C32	C31	109.5(3)
C29	O4	K1	118.17(17)	O4	C28	C27	111.0(2)
C40	O5	K1	117.08(17)	O5	C40	C39	110.2(3)
C41	O5	K1	118.76(19)	C7B	C6B	C12	111.5(12)
C41	O5	C40	111.7(2)	C5B	C6B	C12	124.8(12)
C11	N1	C13	116.2(2)	C5B	C6B	C7B	123.6(15)
C1	N1	C13	118.2(2)	N3	C42	C41	114.6(3)
C1	N1	C11	125.6(2)	O4	C29	C30	109.6(3)
C25	N2	K1	108.63(17)	C10	C9B	C8B	125.9(12)
C25	N2	C37	109.3(2)	C8B	C7B	C6B	123.8(13)
C37	N2	K1	110.55(16)	C4B	C3B	C2	116.8(15)
C36	N2	K1	109.04(16)	N3	C30	C29	114.2(3)
C36	N2	C25	110.2(2)	O5	C41	C42	109.6(3)
C36	N2	C37	109.1(2)	C7B	C8B	C9B	118.4(12)
C31	N3	K1	109.69(16)	C4B	C5B	C6B	120.2(13)
C31	N3	C42	109.7(2)	C3B	C4B	C5B	121.4(13)
C31	N3	C30	109.8(3)	C46	O9	C43	102.1(4)
C42	N3	K1	108.96(18)	O9	C46	C45	107.8(4)
C42	N3	C30	109.6(3)	C43	C44	C45	105.3(5)
C30	N3	K1	109.07(18)	C44	C45	C46	100.6(4)
C13	C18	C22	121.9(2)	O9	C43	C44	107.7(5)
C17	C18	C13	117.7(3)	C5A	C4A	C3A	119.5(10)
C17	C18	C22	120.4(2)	C6A	C5A	C4A	122.2(10)
C14	C19	C20	110.6(2)	C5A	C6A	C12	114.0(9)
C21	C19	C14	111.5(2)	C5A	C6A	C7A	123.2(10)
C21	C19	C20	111.5(3)	C7A	C6A	C12	122.7(9)
C18	C13	N1	119.0(2)	C8A	C7A	C6A	120.4(9)
C14	C13	N1	118.7(2)	C9A	C8A	C7A	122.2(9)
C14	C13	C18	122.3(2)	C8A	C9A	C10	116.2(8)
O2	C11	N1	118.7(2)	C2	C3A	C4A	122.8(12)
O2	C11	C10	125.0(2)				

6. References for supporting information

[1] Cowper, N. G. W.; Chernowsky, C. P.; Williams, O. P.; Wickens, Z. K. Potent Reductants via Electron-Primed Photoredox Catalysis: Unlocking Aryl Chlorides for Radical Coupling. *J. Am. Chem. Soc.* **2020**, *142*, 2093-2099.

[2] Weitz, I. S.; Rabinovitz, M. The application of C_8K for organic synthesis: reduction of substituted naphthalenes. *J. Chem. Soc. Perkin Trans. 1* **1993**, 117-120.

[3] University of Bath, Material and Chemical Characterisation Facility (MC^2), DOI: 10.15125/mx6j-3r54

[4] Lennert, U.; Arockiam, P. B.; Streitferdt, V.; Scott, D. J.; Rödl, C.; Gschwind, R. M.; Wolf, R. Direct catalytic transformation of white phosphorus into arylphosphines and phosphonium salts. *Nature Catal.* **2019**, *2*, 1101-1106.

[5] Reith, A. J.; Gonzalez, M. I.; Kudisch, B.; Nava, M.; Nocera, D. G. How Radical Are "Radical" Photocatalysts? A Closed-Shell Meisenheimer Complex Is Identified as a Super-Reducing Photoreagent. *J. Am. Chem. Soc.* **2021**, *143*, 14352-14359.

[6] Jiao, X-Y.; Bentrude, W. G. A Facile Route to Vinyl- and Arylphosphonates by Vinyl and Aryl Radical Trapping with $(MeO)_3P$. *J. Org. Chem.* **2003**, *68*, 3303-3306.

[7] Magnion, D.; Arnold, D. R. Photochemical Nucleophile–Olefin Combination, Aromatic Substitution Reaction. Its Synthetic Development and Mechanistic Exploration. *Acc. Chem. Res.* **2002**, *35*, 297-304.

[8] Mandigma, M. J. P.; Kaur, J.; Barham, J. P. Organophotocatalytic Mechanisms: Simplicity or Naïvety? Diverting Reactive Pathways by Modifications of Catalyst Structure, Redox States and Substrate Preassemblies. *ChemCatChem* **2023**, doi: 10.1002/cctc.202201542.

[9] Breslin, D. T.; Fox, M. A. Excited-State Behavior of Thermally Stable Radical Ions. *J. Phys. Chem.* **1994**, *98*, 408-411.

[10] (a) Schmalzbauer, M.; Ghosh, I.; König, B. Utilising excited state organic anions for photoredox catalysis: activation of (hetero)aryl chlorides by visible light-absorbing 9-anthrolate anions. *Faraday Discuss.* **2019**, *215*, 364-378. (b) Schmalzbauer, M.; Svejstrup, T. D.; Fricke, F.; Brandt, P.;

Johansson, M. J.; König, B. Redox-Neutral Photocatalytic C−H Carboxylation of Arenes and Styrenes with CO_2. *Chem* **2020**, *6*, 2658-2672.

[11] Breslin, D. T.; Fox, M. A. A strongly chemiluminescent dioxetanimine dianion fragmentation: reaction of the dicyanoanthracene radical anion with superoxide ion. *J. Am. Chem. Soc.* **1993**, *115*, 11716-11721.

[12] Wu, S.; Žurauskas, J.; Domański, M.; Hitzfeld, P. S.; Butera, V.; Scott, D. J.; Rehbein, J.; Kumar, A.; Thyrhaug, E.; Hauer, J.; Barham, J. P. Hole-mediated photoredox catalysis: tris(*p*-substituted)biarylaminium radical cations as tunable, precomplexing and potent photooxidants. *Org. Chem. Front.* **2021**, *8*, 1132-1142.

[13] Kumar, A.; Malevich, P.; Mewes, L.; Wu, S.; Barham, J. P.; Hauer, J. Transient absorption spectroscopy based on uncompressed hollow core fiber whitelight proves pre-association between a radical ion photocatalyst and substrate. *J. Chem. Phys.* **2023**, *158*, 144201.

[14] Xiao, J.; Yin, Z.; Yang, B.; Liu, Y.; Ji, L.; Guo, J.; Huang, L.; Liu, X.; Yan, Q.; Zhang H.; Zhang, Q. Preparation, characterization, physical properties, and photoconducting behaviour of anthracene derivative nanowires. *Nanoscale* **2011**, *11*, 4720-4723.
